# Molecular detection of hrHPV-induced high-grade squamous intraepithelial lesions of the cervix through a targeted RNA next generation sequencing assay

**DOI:** 10.1186/s10020-025-01238-x

**Published:** 2025-05-30

**Authors:** Julia Faillace Thiesen, Elise Jacquemet, Pascal Campagne, Denis Chatelain, Etienne Brochot, Yves-Edouard Herpe, Nolwenn M. Dheilly, Fabrice Bouilloux, Bénédicte Rognon, Alexandre Douablin, Guillaume Leboucher, Florent Percher, Marc Eloit, Philippe Pérot

**Affiliations:** 1 Institut Pasteur, Université Paris Cité, Pathogen Discovery Laboratory, 25-28 Rue du Dr. Roux, 75015 Paris, France; 2 Institut Pasteur, Université Paris Cité, Bioinformatics and Biostatistics Hub, 25-28 Rue du Dr. Roux, 75015 Paris, France; 3 Amiens Picardie University Hospital, Service d’anatomie Et Cytologie Pathologiques, 80000 Amiens, France; 4 Amiens Picardie University Hospital, Research Department, Biobanque de Picardie (BRIF N BB-0033-00017), 80000 Amiens, France; 5 Biomnigene SA, 25000 Besançon, France; 6 Labtoo, 17000 La Rochelle, France; 7 National Veterinary School of Alfort, Paris-Est University, 94700 Maisons-Alfort, France

**Keywords:** Human Papillomavirus (HPV), Molecular test, Screening, Precancerous lesions, Transcriptome, Next-Generation Sequencing (NGS)

## Abstract

**Background:**

Cervical cancer screening programs are increasingly relying on sensitive molecular approaches as primary tests to detect high-risk human papillomaviruses (hrHPV), the causative agents of cervix cancer. Although hrHPV infection is a pre-requisite for the development of most precancerous lesions, the mere detection of viral nucleic acids, also present in transient infections, is not specific of the underlying cellular state, resulting in poor positive predictive values (PPV) regarding lesional states. There is a need to increase the specificity of molecular tests for better stratifying individuals at risk of cancer and to adapt follow-up strategies.

**Methods:**

HPV-RNA-SEQ, a targeted RNA next generation sequencing assay allowing the detection of up to 16 hrHPV splice events and key human transcripts, has previously shown encouraging PPV for the detection of precancerous lesions. Herein, on 302 patients with normal cytology (NILM, *n* = 118), low-grade (LSIL, *n* = 104) or high-grade squamous intraepithelial lesions (HSIL, *n* = 80), machine learning-based model improvement was applied to reach 2-classes (NILM vs HSIL) or 3-classes (NILM, LSIL, HSIL) predictive models.

**Results:**

Linear (elastic net) and nonlinear (random forest) approaches resulted in five 2-class models that detect HSIL vs NILM in a validation set with specificity up to 0.87, well within the range of PPV of other competing RNA-based tests in a screening population.

**Conclusions:**

HPV-RNA-SEQ improves the detection of HSIL lesions and has the potential to complete and eventually replace current molecular approaches as a first-line test. Further performance evaluation remains to be done on larger and prospective cohorts.

**Supplementary Information:**

The online version contains supplementary material available at 10.1186/s10020-025-01238-x.

## Background

Cervical cancer is currently the fourth cause of cancer in women globally, being responsible for approximately 604,000 new cases and 340,000 deaths in 2020 (Sung et al. 2020). Nearly all cervical cancer cases are caused by sexually transmitted high-risk Human papillomaviruses (hrHPV). The International Agency for Research on Cancer recognized 17 HPV genotypes to be causal to invasive cervical cancer, with huge differences in their carcinogenic strength (Wei et al. 2024). As of today, 12 hrHPV types are considered highly relevant and have been indicated in the Target Product Profile recommendation by WHO (IARC. 2022; World Health Organization (WHO) 2024). hrHPVs are etiological factors of several other cancers, including vulvar, penile, neck and head cancers (Schiffman et al. 2016), and are overall responsible for 5% of all human cancers (Estêvão et al. 2019).

The 8 kb-sized genome of hrHPV encodes five early proteins (E2, E4, E5, E6, E7) and 2 capsid proteins referred as late genes (L1, L2) (Schiffman et al. 2016; McBride 2022). Viral infection initiates through microlesions in the cervix allowing hrHPV to enter the dividing basal layer of stratified squamous epithelia. Under the main control of the viral E2 protein, genes transcription is tightly regulated and varies over the course of tissue differentiation. The expression of the early genes E1, E2, E4, E5, E6 and E7 ensures viral genome replication and maintenance at relatively low levels (McBride 2022). Upon cell differentiation, the capsid proteins L1 and L2 are expressed in the mid and upper layers of the epithelium, assuring virions assembly and release of mature infectious virions (Schiffman et al. 2016; Woodman et al. 2007).

More than 90% of HPV infections are transient and are cleared out by the host immune system within one year (Schiffman et al. 2016). Oncogenesis is associated with persistent infection by hrHPV. Low grade Squamous Intraepithelial Lesions (LSIL, equivalent to Cervical Intraepithelial Neoplasia or CIN1) have high spontaneous regression rates (89.7% in Bruno et al*. *(2022), 88.5% in Ciavattini et al*. *(2017), while High grade Squamous Intraepithelial Lesions (HSIL, equivalent to CIN2 and CIN3) have lower regression rates (*e.g.* 47% in Ehret et al*. *(Ehret et al. 2023)) and can progress to cancer. The percentage of HSIL in screening populations remains low, typically below 1% (Cuzick et al. 2013), and the risk of precancerous lesions corresponding to CIN3 or worse (CIN3 +) in the general population is less than 0.15% over 5 years following a negative HPV test result (Perkins et al. 2023). The transition from a persistent and productive HPV infection to an oncogenic infection requires years to decades (Hu and Ma 2018). Thanks to this prolonged timeframe, the early detection and treatment of precancerous lesions has a strong medical benefit (Schiffman et al. 2016; Schlecht et al. 2003). However, early detection must show good positive predictive value (PPV) to avoid unnecessary interventions.

Cervical cancer screening programs vary among countries but often consist first in detecting hrHPVs using molecular tests, and then, in case of positivity, in cytological examination aiming at detecting abnormal cervical cells (the Papanicolaou test) (Perkins et al. 2023). DNA-based molecular tests are very sensitive and specific in detecting the presence of hrHPVs genomes. Yet, viral genome detection cannot distinguish between transient and clinically relevant infections and remains a poor predictor of underlying precancerous lesions. On the other hand, cytological examinations detect and classify lesions with good specificity. However, cytology lacks sensitivity (Haute Autorité de Santé 2019) and reproducibility because of variations in reader subjectivity. Thus, the visual confirmation of the presence of lesions in the cervix by a clinician through colposcopy remains necessary, followed by histopathology conducted on biopsy in case of detectable lesion (Schiffman et al. 2016). Colposcopies only confirm one third of the precancerous or cancerous cases suspected by hrHPV detection and cytology (Ogilvie et al. 2018), illustrating the risk of overdiagnosis from screening programs, and associated follow up procedures, patient anxiety and screening costs (Hu and Ma 2018). There is therefore a need for more effective triage tools to better stratify individuals at risk and reduce the number of unnecessary follow-up referrals.

RNA tests targeting E6/E7 transcripts have been proposed as an alternative to DNA tests (Arbyn et al. 2022). Indeed, the persistent expression of E6 and E7 genes is one of the primary factors for cervical cancer progression (Estêvão et al. 2019; Moody and Laimins 2010). Among not fully understood other mechanisms, viral integration into the host genome is a frequent event which disrupts E1 and E2 transcription units, leading to an uncontrolled overexpression of E6 and E7 (McBride and Warburton 2017; Kamal et al. 2021). These two protein products are associated with several hallmarks of cancer (Schiffman et al. 2016; Estêvão et al. 2019) such as maintaining a continuous proliferative state (*e.g.* via the downregulation of tumor suppressor gene TP53), overcoming cell cycle checkpoints (*e.g.* via the downregulation of tumor suppressor gene pRB, a major G1 checkpoint regulator), evading the host immunity, escaping cell death (*e.g.*via the upregulation of the human telomerase hTERT and downregulation of TP53) and promoting genomic instability (by the upregulation of APOBEC3 that leads to high mutation rate (Estêvão et al. 2019)).

There are currently numerous hrHPVs mRNA tests measuring the expression of E6/E7 available in the market, but there is still no consensus on whether these mRNA tests have a better PPV than a combination of DNA testing and cytology (Arbyn et al. 2022; Cook et al. 2017; Virtanen et al. 2017). The performances of mRNA tests for CIN2 + detection vary greatly and have been the subject of several studies. In a meta-analysis, Derbie et al*. *(2020) compared the diagnostic performance for CIN2 + of three of the most common mRNA tests, namely PreTect Proofer (PreTect AS, Norway), Aptima (Hologic, USA) and Quantivirus (DiaCarta, USA) in populations referred for histology with high CIN2 + prevalence, using histology as a gold standard. They reported a median sensitivity ranging from 83.0% to 91.4% and a median specificity ranging from 46.2% to 73.0%. As a result, the mRNA tests have estimated PPVs ranging from 34.3% (Aptima) to 70.0% (PreTect Proofer). Arbyn et al*. *(2022) compared the accuracy of hrHPV DNA tests and mRNA tests for the detection of CIN2 + and CIN3 + during primary cervical cancer screening. They found that Aptima had similar cross-sectional sensitivity (relative sensitivity 0.98 [95% CI 0.95–1.01]) and slightly higher specificity (1.03 [1.02–1.04]) for CIN2 + and CIN3 + than DNA tests**.**Also, Aptima showed a long-term safety, defined both as the sensitivity for CIN3 + detection and the relative detection of CIN3 + among women who screened negative, comparable to that of DNA tests (Strang et al. 2021; Iftner et al. 2019). The choice of using mRNA tests in screening programs is therefore promising, albeit limited given the small increase in specificity of current mRNA tests compared to DNA tests (Dombrowski et al. 2022). Moreover, even though it is accepted that the upregulated expression of E6/E7 is associated with disease progression (Duvlis et al. 2015; Choi et al. 2023), E6 and E7 proteins are also expressed in productive yet transient infections during which the amount of E6/E7 mRNA may correlate with viral load, which makes the definition of a detection threshold complicated. In addition, the low prevalence of CIN2 + lesions in a screening population negatively affects the PPV. There is therefore a need for a novel generation of molecular diagnostic tests capable of distinguishing between transient and transforming infections with a better specificity. Without such a test, a high number of unnecessary colposcopies will continue to be conducted.

We (Pérot et al. 2019) and others (Andralojc et al. 2022) have previously explored the principle of a molecular test capable of encompassing a more exhaustive view of the HPV transcriptome, with or without the addition of human transcripts (including oncogenes, tumor suppression genes, direct or indirect downstream effectors of HPV oncoproteins such as AKT1, BCL2, BRAF, CDH1, CDKN2A, CDKN2B, ERBB2, FOS, HRAS, KRAS, MET, MKI67, MYC, NOTCH1, PCNA, PTEN, RB1, STAT1, TERT, TOP2A, TP53, and WNT1 (Pérot et al 2019)). The underlying assumption is that a balance of expression integrating all early and late HPV transcripts, whose ratio varies according to the state of cellular differentiation, with or without considering the variation in human transcripts, could help distinguish more finely between transient and transforming infections. In practical terms, our targeted RNA next generation sequencing (NGS) assay, named HPV-RNA-SEQ, uses standard liquid-based cytology samples as input to detect the 12 high-risk HPVs as per the current WHO recommendation (HPVs 16, 18, 31, 33, 35, 39, 45, 51, 52, 56, 58 and 59) plus 4 HPVs of lower prevalence (66, 68, 73, and 82) and quantify a wide set of HPV transcripts on splice regions. This approach showed encouraging PPV for the detection of HSIL in a previous cohort of 55 patients (Pérot et al 2019).

This work aimed to develop HPV-RNA-SEQ further with machine learning models that take HPV and human transcripts expression as input, to classify squamous intraepithelial lesions. To achieve this goal, we used a cohort of 302 patients aged between 25 and 65 years, with normal cytology (NILM, *n* = 118), low-grade (LSIL, *n* = 104), and high-grade squamous intraepithelial lesions (HSIL, *n* = 80). We first built 3-classes (NILM, LSIL, HSIL) and 2-classes (NILM vs HSIL) machine learning models based on transcriptomic information from hrHPV and human transcripts on a training set of data from 220 patients, and then validated the performance of the models on an independent validation set of 82 patients.

## Materials and methods

The Materials and Methods section is provided in the Supplementary Data.

## Results

### Lesion prediction with HPV-RNA-SEQ

**Fig. 1:**
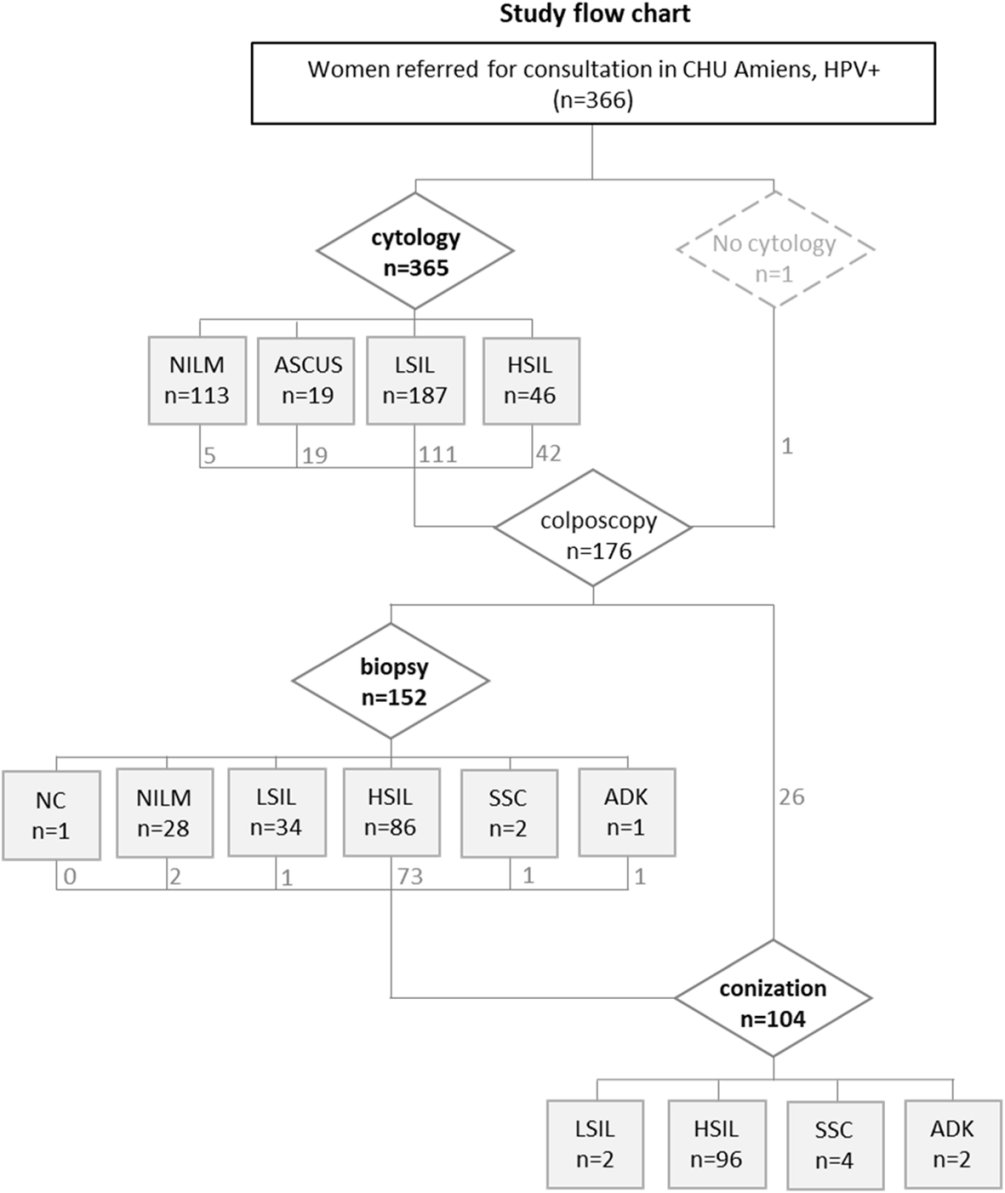
Study flow chart. Available clinical results from study cohort. Distribution of clinical outcome according to (1) cytology results, (2) histological results from a biopsy, and (3) histological results based on a conization procedure. Legend: NILM: Negative for Intraepithelial Lesion or Malignancy; ASC-US: Atypical Squamous Cells of Undetermined Significance; LSIL: Low Grade Squamous Intraepithelial Lesion; HSIL: High Grade Squamous Intraepithelial Lesion; SCC: Squamous Cell Carcinoma (Invasive or Microinvasive); ADK: Adenocarcinoma (glandular cell); NC = non-contributive

HPV-RNA-SEQ data from 302 hrHPV DNA-positive cervical swabs samples (Fig. 1) were analyzed to investigate the HPV transcriptome and a set of human transcripts as precancerous lesion predictors. We employed linear (elastic net) and nonlinear (random forest) methods to assess different variable combinations: (S) “spliced”, included specific HPV splice events found on HPV trancripts; (uS) “Unspliced”, included specific HPV splice donor or acceptor sites in the absence of splice event; (H) “Human”, consisted of twenty-two human transcripts, including oncogenes. Their combined use was also explored: (S + uS) included HPV spliced and unspliced transcripts; (S + H) included HPV spliced transcripts and human transcripts; (uS + H) included unspliced HPV transcripts and human transcripts; (S + uS + H) included HPV spliced and unspliced transcripts plus human transcripts. These set of variables were tested to distinguish between NILM, LSIL and HSIL (3-class models with random forest only), or to distinguish HSIL from NILM (2-class models with both elastic net and random forest), using a mixed reference combining histology and cytology results (see methods in the Supplementary Data). We compared the transcripts-based models to two control models based on the presence/absence of HPV (P) and the total number of HPV reads (T). Altogether, 27 models were explored, described in Table 1 (see Supplementary Table 5 for more detailed information). Contingency tables and sample scores for each model are available in Supplementary Data 3.

**Table 1:** Performances of HPV-RNA-SEQ models and controls

Model	# Predicted classes	Predictors set	Statistical method	Accuracy %	Kappa	HSIL Se%	HSIL Sp%	LSIL Se%	LSIL Sp%	NILM Se%	NILM Sp%
1	2-class	S	random forest	80.0 (66.3–90.0)	0.58	70.0 (45.7–88.1)	86.7 (69.3–96.2)	-	-	-	-
2	2-class	uS + H	elastic net	78.0 (64.0–88.5)	0.56	85.0 (62.1–96.8)	73.3 (54.1–87.7)	-	-	-	-
3	2-class	S + uS + H	elastic net	78.0 (64.0–88.5)	0.55	80.0 (56.3–94.3)	76.7 (57.7–90.1)	-	-	-	-
4	2-class	S + uS	random forest	76.0 (61.8–86.9)	0.52	80.0 (56.3–94.3)	73.3 (54.4–87.7)	-	-	-	-
5	2-class	S + H	elastic net	76.0 (61.8–86.9)	0.5	70.0 (45.7–88.1)	80.0 (61.4–92.3)	-	-	-	-
6	2-class	S + uS + H	random forest	72.0 (57.5–83.8)	0.44	80.0 (56.3–94.3)	66.7 (47.2–82.7)	-	-	-	-
7	2-class	S + uS	elastic net	72.0 (57.5–83.8)	0.39	50.0 (27.2–72.8)	86.7 (69.3–96.2)	-	-	-	-
8	2-class	uS	random forest	70.0 (55.4–82.1)	0.37	60.0 (36.1–80.9)	76.7 (57.7–90.1)	-	-	-	-
9	2-class	S	elastic net	70.0 (55.4–82.1)	0.32	40.0 (19.1–63.9)	90.0 (73.5–97.9)	-	-	-	-
10	2-class	uS	elastic net	68.0 (53.3–80.5)	0.31	50.0 (27.2–72.8)	80.0 (61.4–92.3)	-	-	-	-
11	2-class	uS + H	random forest	64.0 (49.2–77.1)	0.25	55.0 (31.5–76.9)	70.0 (50.6–85.3)	-	-	-	-
12	2-class	P	random forest	62.0 (47.2–75.3)	0.10	15.0 (3.2–37.9)	93.3 (77.9–99.2)	-	-	-	-
13	2-class	S + H	random forest	60.0 (45.2–73.6)	0.24	80.0 (56.3–94.3)	46.7 (28.3–65.7)	-	-	-	-
14	2-class	P	elastic net	54.0 (39.3–68.2)	0.15	80.0 (56.3–94.3)	36.7 (19.9–56.1)	-	-	-	-
15	2-class	T	elastic net	52.0 (37.4–66.3)	0.08	65.0 (40.8–84.6)	43.3 (25.5–62.6)	-	-	-	-
16	2-class	H	elastic net	50.0 (35.5–64.5)	0.10	85.0 (62.1–96.8)	26.7 (12.3–45.9)	-	-	-	-
17	2-class	H	random forest	46.0 (31.8–60.7)	0.01	75.0 (50.9–91.3)	26.7 (12.3–45.9)	-	-	-	-
18	2-class	T	random forest	42.0 (28.2–56.8)	−0.12	50.0 (27.2–72.8)	36.7 (19.9–56.1)	-	-	-	-
19	3-class	S	random forest	51.2(39.9–62.4)	0.24	35	97	34	74	80	52
20	3-class	S + uS	random forest	47.6 (36.4–58.9)	0.20	55	87	19	82	73	50
21	3-class	S + uS + H	random forest	46.3 (35.3–57.7)	0.21	65	66	12	96	70	60
22	3-class	uS	random forest	39.0 (28.4–50.4)	0.06	30	87	19	76	67	42
23	3-class	uS + H	random forest	39.0 (28.4–50.4)	0.11	50	58	9	98	63	56
24	3-class	S + H	random forest	39.0 (28.4–50.4)	0.08	50	74	34	68	37	65
25	3-class	P	random forest	36.6 (26.2–48.0)	0.01	0	93	9	90	90	17
26	3-class	T	random forest	35.4 (25.1–46.7)	0.02	30	74	37	66	37	61
27	3-class	H	random forest	31.7 (21.9–42.9)	−0.01	55	61	37	60	10	77

### Poor classification by control models

**Fig. 2:**
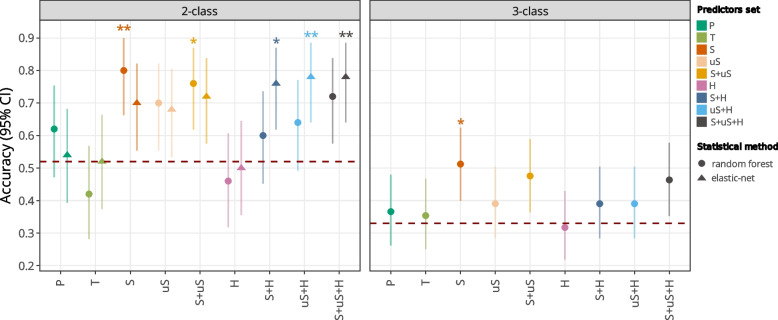
Models overall performances on validation set. 3-class and 2-class models' accuracy are presented for all models trained. Accuracy was computed along with 95% Confidence interval according to prediction on the validation set. Red dotted line represents the average accuracy for a random classifier, computed through simulation (1000 random shuffle of predictions for the validation set). Models for which the accuracy is significantly higher than the no information rate are identified with their significance level: * for *p*value < 0.05; ** for *p*value < 0.01. Set of variables: S: “Spliced”, uS: “Unspliced”, H: “Human”, S + uS: “Spliced + Unspliced”, S + H: “Spliced + Human”, uS + H: “Unspliced + Human”, S + uS + H: “Spliced + Unspliced + Human”, P: “Presence of HPVs”, T:”Total HPV sequence count”

**Fig. 3:**
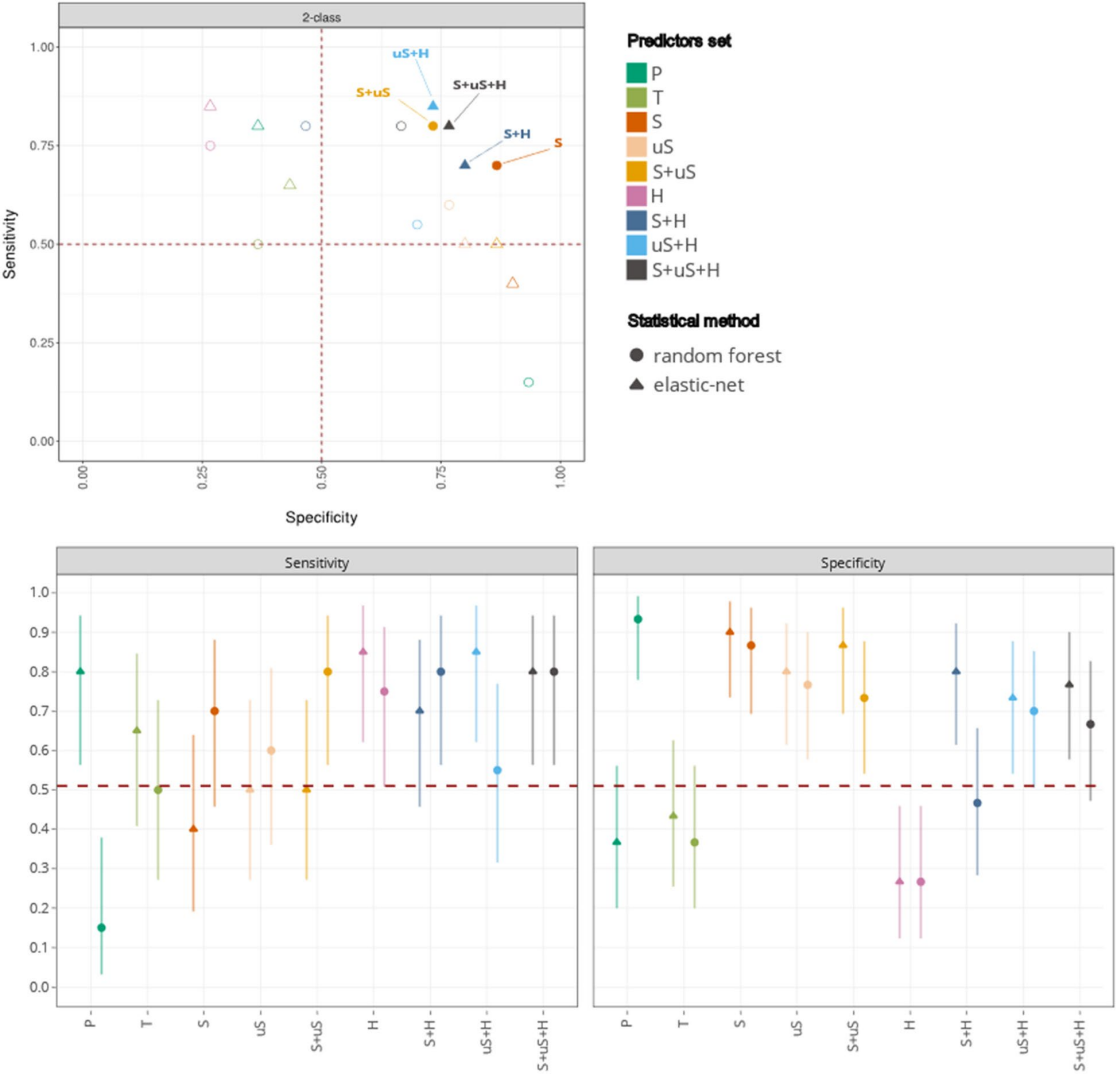
Specificity and sensitivity of classifications models. Sensitivity and Specificity were computed on validation set for all 2-class models, along with 95% confidence interval. Thresholds at 0.5 for both metrics are shown in red, and a focus is made on the 5 models that show best performance and compromise between Sp and Se (full dots). Set of variables: S: “Spliced”, uS: “Unspliced”, H: “Human”, S + uS: “Spliced + Unspliced”, S + H: “Spliced + Human”, uS + H: “Unspliced + Human”, S + uS + H: “Spliced + Unspliced + Human”, P: “Presence of HPVs”, T:”Total HPV sequence count”

Control models based on the presence/absence of the different HPV genotypes (P model) and the proportion of total HPV reads in the sample (T model) generated poor performances (Table 1, Fig. 2). Indeed, the nonlinear P model yielded high specificity (93.3%) but very low sensitivity (15.0%), whereas the linear P model yielded high sensitivity (80.0%) but low specificity (36.7%) (Table 1, Fig. 3). Based on the T predictor, both methods resulted in low sensitivity (50.0–65.0%) and low specificity (36.7–43.3%), with accuracies no higher than a random classifier (Fig. 2).

### Poor classification by three-class models (NILM, LSIL and HSIL)

The accuracy of 3-class models ranged from 30.0% to 51.0%, and only the S model had an accuracy superior to that of a random classifier (95% CI, or *P* < 0.05) (Table 1, Fig. 2). The S + uS + H model had the highest sensitivity for HSIL (65.0%) and retained a high sensitivity for NILM (70.0%), with moderate specificity in both cases (66.0% and 60.0% respectively) but showed a low sensitivity for LSIL detection (12.5%) (Table 2). Model prediction based on spliced junctions (S model) yielded the highest sensitivity for NILM (80.0%) and the highest, but still low sensitivity for LSIL samples (34.4%), with a sensitivity for HSIL samples of only 35.0%. In fact, the S model grouped LSIL samples mostly within the NILM class. Over all models, the LSIL samples were distributed across the entire score range, demonstrating the heterogeneity within this class, and precluding the efficiency of three-class models.

**Table 2:** Contingency tables and performances of HPV-RNA-SEQ models for the prediction of HSIL, LSIL and NILM

Model	Reference			HSIL Pe, %		LSIL Pe, %		NILM Pe, %	
S + uS + H	HSIL	LSIL	NILM	Se	65	Se	12.5	Se	70
HSIL	13	12	9	Sp	66.1	Sp	96	Sp	59.6
LSIL	2	4	0	PPV	38.2	PPV	66.7	PPV	50
NILM	5	16	21	NPV	85.4	NPV	85.4	NPV	77.5
S	HSIL	LSIL	NILM	Se	35	Se	34.4	Se	80
HSIL	7	1	1	Sp	96.8	Sp	74	Sp	51.9
LSIL	8	11	5	PPV	77.8	PPV	45.8	PPV	49
NILM	5	20	24	NPV	82.2	NPV	63.8	NPV	81.8
S + uS	HSIL	LSIL	NILM	Se	55	Se	18.8	Se	85.7
HSIL	11	5	3	Sp	87.1	Sp	82	Sp	59.6
LSIL	4	6	5	PPV	57.9	PPV	40	PPV	50
NILM	5	21	22	NPV	85.7	NPV	61.2	NPV	77.5

### Performances of two-class models (NILM vs HSIL) using exclusively HPV transcripts predictors

Linear and nonlinear models were generated using the S and uS predictors. The linear and nonlinear S models yielded an overall high accuracy (70.0% to 80.0%, see Table 1, Fig. 2) associated with high specificity (86.7% to 90.0% see Table 1, Fig. 3). However, the linear S model presented low sensitivity (40.0%). Interestingly, linear, and nonlinear uS models had high specificity (76.7% to 80.0%) and moderate sensitivity (50.0% to 60.0%), resulting in moderate overall accuracy (68.0% to 70.0%) (Table 1, Fig. 3). By combining the S + uS predictors, linear and nonlinear approaches resulted in models with high specificity (73.3% to 86.7%).

As previously, the linear approach had lower sensitivity (50.0%) than the nonlinear approach (80.0%). Compared to the previously identified high performance nonlinear S model (Se 70.0%, Sp 86.7%) based on spliced variants, the nonlinear S + uS model had comparable high performances, although with a tendency, albeit non-significant, for higher sensitivity (80.0%) and lower specificity (73.3%).

Both models (nonlinear S and S + uS) had accuracies significantly higher than a random classifier (Fig. 2). They are here considered as equally promising models due to their similar performance levels, as demonstrated by performances values falling within each other's confidence intervals (e.g. see accuracy in Fig. 2). Thus, the most promising 2-class models based exclusively on HPV transcripts were the nonlinear S (accuracy = 80.0%, Se = 70.0%, Sp = 86.7%) and S + uS (accuracy = 76.0%, Se = 80%, Sp = 73.3%) models (Fig. 3).

### Performances of two-class models (NILM vs HSIL) with both HPV and human transcripts predictors

Next, we investigated how human transcripts alone (H model), or in combination with HPV transcripts (S + H, uS + H, S + uS + H) could classify NILM and HSIL. The use of human transcripts alone was associated with high sensitivity (75.0–85.0%) but low specificity (26.7%) for both linear and nonlinear models (Table 1, Fig. 3), resulting in poor performances with PPV and accuracy no greater than 50.0% (Table 1, Fig. 2), making this approach unattractive. Combining human transcripts with either spliced transcripts (S + H) or unspliced transcripts (uS + H) resulted in higher accuracy for linear models (76.0–78.0%) but moderate accuracy for nonlinear models (60.0–64.0%) (Fig. 2).

The most performant nonlinear model using human transcripts as predictors was obtained in combination with viral transcripts (S + us + H) and resulted in high sensitivity (80.0%) and moderate specificity (66.7%) (Table 1, Fig. 3). This result should be analyzed in regard with the previously described nonlinear S + uS model that presented higher specificity (73%) while maintaining similar sensitivity (80%). The linear approach produced high performance S + uS + H model with a sensitivity of 80.0% and a specificity of 76.7% (Table 1, Fig. 3).

Models including human transcripts predictors but excluding one of the HPV transcript predictors (S or uS) had nearly equivalent performances as the S + uS + H model (performance values being within their confidence intervals, see Figs. 2 and 3). Although not statistically significant, the uS + H predictor set produced a model demonstrating a tendency towards higher sensitivity (Se = 85.0%, Sp = 73.3%), while the S + H combination resulted in a model leaning towards greater specificity (Sp = 80.0%, Se = 70.0%) (Table 1, Fig. 3).

### Selecting the most performant models: five models outperform a random classifier

Among the 27 tested models, five models exhibited high overall performances, with prediction accuracy surpassing those of a random classifier (95% CI, or *P* < 0.05) (see Fig. 2). The S and S + uS nonlinear models, and the S + H, uS + H and S + uS + H linear models (shown in full dots in Fig. 3) were considered as equally promising, given that their accuracy levels align within each other's confidence intervals (e.g. see accuracy in Fig. 2). The contingency tables for the five selected models are shown in Table 3. The S random forest model showed the highest specificity (Sp 86.7%) and PPV (PPV 77.8%) across all models. The uS + H elastic net model had the highest sensitivity (Se 85.0%), with an overall PPV of 68.0%. Prediction scores for validation samples through the five best models can be found in Supplementary Figs. 5 and 6. Of interest, the same individual samples tend to be misclassified by the five best models (Supplementary Fig. 5). Most specifically, within the validation set only, five NILM samples and four HSIL were classified by at least four models as HSIL and NILM, respectively.

**Table 3:** Contigency tables and performances of HPV-RNA-SEQ models for the prediction of high-grade cytology

	Reference		HSIL Pe, %	
S + uS + H,			Se	80.0 (56.3–94.3)
elastic net	HSIL	NILM	Sp	76.7 (57.7–90.1)
HSIL	16	7	PPV	69.6 (47.1–86.8)
NILM	4	23	NPV	85.2 (66.3–95.8)
uS + H,			Se	85.0 (62.1–96.8)
elastic net	HSIL	NILM	Sp	73.3 (54.1–87.7)
HSIL	17	8	PPV	68.0 (46.5–85.1)
NILM	3	22	NPV	88.0 (68.8–97.5)
S + uS			Se	80.0 (56.3–94.3)
random forest	HSIL	NILM	Sp	73.3 (54.1–87.7)
HSIL	16	8	PPV	66.7 (44.7–84.4)
NILM	4	22	NPV	84.6 (65.1–95.6)
S,			Se	70.0 (45.7–88.1)
random forest	HSIL	NILM	Sp	86.7 (69.3–96.2)
HSIL	14	4	PPV	77.8 (52.4–93.6)
NILM	6	26	NPV	81.3 (63.6–92.8)
S + H,			Se	70.0 (45.7–88.1)
elastic net	HSIL	NILM	Sp	80.0 (61.4–92.3)
HSIL	14	6	PPV	70.0 (45.7–88.1)
NILM	6	24	NPV	80.0 (61.4–92.3)

### A subset of variable predictors of importance

**Fig. 4:**
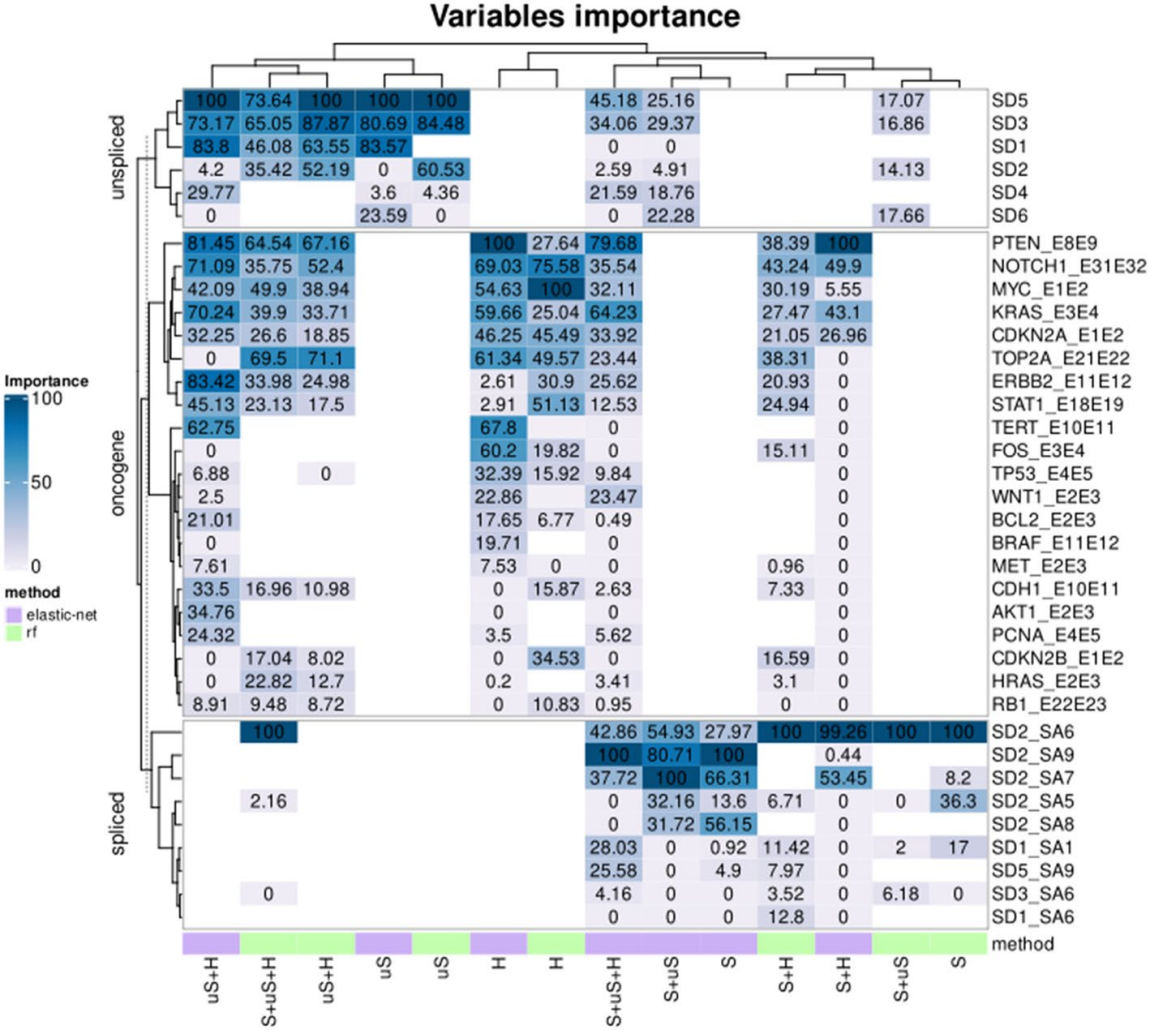
Features predictive value through all 2-class models. Heatmap representing the importance (%) of each feature (rows) used for training models. Three sets (unspliced, spliced and oncogenes) were evaluated through the different trained models: highest importances in prediction are represented in dark blue whereas features that were less decisive in predicting model outcome are shown in off-white. White features were either excluded in the set or removed during feature selection. Methods (columns) are ordered by Hierarchical clustering method, according to Ward D2 criterion. Elastic net method is represented in violet and random forest in green. Set of variables: S: “Spliced”, uS: “Unspliced”, H: “Human”, S + uS: “Spliced + Unspliced”, S + H: “Spliced + Human”, uS + H: “Unspliced + Human”, S + uS + H: “Spliced + Unspliced + Human”

The importance of variable predictors contributing to at least one of the five best models is shown in Fig. 4 and Table 4. The Table 4 also shows elastic net coefficients for linear models. A total of nineteen human transcripts (19/22), seven HPV spliced regions (7/25) and six HPV unspliced transcripts (6/15) were informative for at least one of the five selected models. Among the spliced transcripts, SD2-SA6 was the most informative for three over four models that included S, except for the S + uS + H elastic net model that put more weight in the information carried by the SD2-SA9 spliced transcript (Fig. 4). Among unspliced transcripts, the SD5 was one of the most important variables for all best models including uS, followed closely with SD3. Finally, among the human transcripts, all three elastic-net models put more importance in expression data of PTEN, KRAS and NOTCH1 for the classification. The uS + H model also assigned significant importance in the ERBB2 expression (Fig. 4). The overexpression of specific HPV or human transcripts, such as spliced SD2-SA9, SD2-SA6, SD2-SA7, unspliced SD5 and SD3, and human NOTCH1 and CDKN2A were associated with high grade lesions (see coefficient values for elastic net models in Table 4 and Supplementary Fig. 7). Other transcripts were associated with the absence of lesion (NILM), like PTEN, KRAS, TOP2A, SD1_SA1 (HPV early) and SD5_SA9 (HPV late). Supplementary Table 6 and Supplementary Data 4 include complete information on importance and elastic net coefficients across all explored models. Heatmap figures on variable importance and elastic-net coefficients for models based on HPV presence/absence can be found in Supplementary Fig. 8 and 9, respectively.

**Table 4:** Elastic net coefficients, variable importance for elastic net and random forest models

	S + uS + H (en)		uS + H (en)		S + H (en)		S (rf)	S + uS (rf)
Predictor	Coef	Imp	Coef	Imp	Coef	Imp	Imp	Imp
PTEN_E8E9	−0.890	79.676	−1.446	81.451	−0.689	100.000	nd	nd
KRAS_E3E4	−0.718	64.229	−1.247	70.238	−0.297	43.099	nd	nd
SD1_SA1	−0.313	28.033	nd	nd	0	0	16.997	2.005
SD5_SA9	−0.286	25.579	nd	nd	0	0	ns	ns
TOP2A_E21E22	−0.262	23.443	0	0	0	0	nd	nd
SD4	−0.241	21.585	−0.528	29.770	nd	nd	nd	ns
STAT1_E18E19	−0.140	12.534	−0.801	45.135	0	0	nd	nd
SD3_SA6	−0.046	4.160	nd	nd	0	0	0	6.182
CDH1_E10E11	−0.029	2.632	−0.595	33.504	0	0	nd	nd
BCL2_E2E3	−0.006	0.493	−0.373	21.008	0	0	nd	nd
SD2_SA5	0	0	nd	nd	0	0	36.304	0
SD1	0	0	−1.487	83.796	nd	nd	nd	ns
AKT1_E2E3	0	0	−0.617	34.758	0	0	nd	nd
MET_E2E3	0	0	−0.135	7.614	0	0	nd	nd
SD6	0	0	0	0	nd	nd	nd	17.657
MKI67_E6E7	0	0	0.144	8.101	0	0	nd	nd
TERT_E10E11	0	0	1.114	62.751	0	0	nd	nd
RB1_E22E23	0.011	0.953	0.158	8.909	0	0	nd	nd
SD2	0.029	2.588	0.074	4.197	nd	nd	nd	14.132
HRAS_E2E3	0.038	3.415	0	0	0	0	nd	nd
PCNA_E4E5	0.063	5.615	0.432	24.317	0	0	nd	nd
TP53_E4E5	0.110	9.839	0.122	6.884	0	0	nd	nd
WNT1_E2E3	0.262	23.465	0.044	2.498	0	0	nd	nd
ERBB2_E11E12	0.286	25.621	1.481	83.423	0	0	nd	nd
MYC_E1E2	0.359	32.112	0.747	42.088	0.038	5.545	nd	nd
CDKN2A_E1E2	0.379	33.924	0.572	32.252	0.186	26.963	nd	nd
SD3	0.381	34.062	1.299	73.171	nd	nd	nd	16.859
NOTCH1_E31E32	0.397	35.540	1.262	71.095	0.344	49.902	nd	nd
SD2_SA7	0.421	37.719	nd	nd	0.368	53.447	8.196	ns
SD2_SA6	0.479	42.859	nd	nd	0.684	99.265	100.000	100.000
SD5	0.505	45.178	1.775	100.000	nd	nd	nd	17.070
SD2_SA9	1.117	100.000	nd	nd	0.003	0.440	ns	ns

### Distribution of LSIL samples classification by the five best two-class models

LSIL samples, which were not used for training and validation of 2-class models, were classified into NILM or HSIL category by each of the five best models. Sample scores were distributed along the entire scoring range (Supplementary Figs. 10 & 11) with an average of 63.2% of LSIL samples classified as NILM and an average of 36.8% of LSIL samples classified as HSIL. In fact, the S model grouped LSIL samples mostly within the NILM class. Over all models, the LSIL samples were distributed across the entire score range, demonstrating the heterogeneity within this class, and precluding the efficiency of three-class models.

### Predicted positive predictive value of RNA-based HPV molecular tests in a population with different prevalence rates

**Fig. 5:**
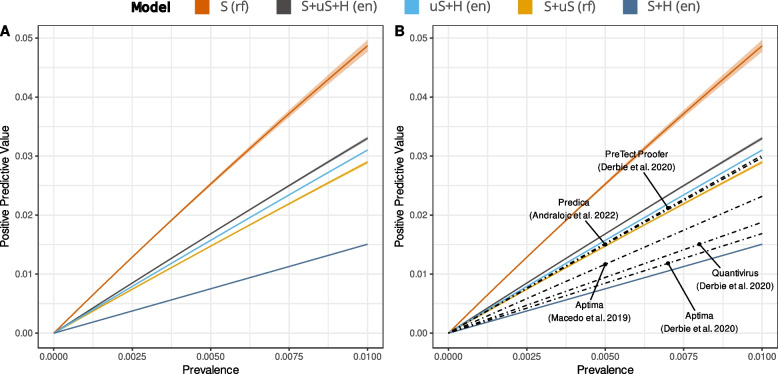
Positive predictive value estimates function of HSIL prevalence: Positive predictive value for the five best models is represented along with some other references from literature. PPV was computed as a function of assumed HSIL prevalence in the population (x-axis). In addition, uncertainty bound to the ratio of LSIL relative to HSIL was considered (bands around the line) and this ratio was assumed to be lying between 1 and 4 (Supplementary Data 5). Set of variables: S: “Spliced”, S + uS: “Spliced + Unspliced”, S + H: “Spliced + Human”, uS + H: “Unspliced + Human”, S + uS + H: “Spliced + Unspliced + Human”. Statistical methods: rf: “random forest”, en: elastic net

The calculated PPVs for HSIL detection in a screening population composed of an unknown, variable percentage, but less than 1% of HSIL lesions (Cuzick et al. 2013) are presented in Fig. 5 for the five selected models, along with the calculated PPVs of the commercially available RT-PCR-based molecular tests Aptima (Derbie et al. 2020; Macedo et al. 2019), PreTect Proofer (Derbie et al. 2020), Quantivirus (Derbie et al. 2020) and the targeted RNA NGS-based test under development from Predica (Andralojc et al. 2022). PPV were calculated from the pooled or median Se and Sp values reported in the referenced studies. Results showed that the S + H model had inferior PPV than commercial tests, while the S + uS model had PPV superior to that of Aptima (Derbie et al. 2020; Macedo et al. 2019) and Quantivirus (Derbie et al. 2020) (Fig. 5). The S, S + uS + H and uS + H models showed superior PPV to all five listed HPV tests. Notably, the S model had the higher PPV, suggesting that this model could compete with existing commercial tests, such as Aptima which is currently the most widely used RNA test on the market.

## Discussion

HPV-RNA-SEQ is a targeted RNA sequencing approach with the capacity to detect and quantify specific spliced, but also unspliced hrHPV RNA, as well as some key human transcripts (Pérot et al. 2019). In contrast to other RNA tests focusing on one or two specific mRNA, the novelty of HPV-RNA-SEQ lies in its ability to embrace a broad vision of the transcriptome through the early and late transcripts, making possible to compute a balance of expression into a predictive score. As an example, for HPV16, HPV-RNA-SEQ can detect 14 unique splice junctions, plus 11 unspliced events (Supplementary Table 1). A more in-depth description of the design has been published previously (Pérot et al. 2019). The high level of multiplexing allowed by NGS extended the detection principle to 16 hrHPV into a single experiment, which makes HPV-RNA-SEQ unique.

Machine learning-based predictions integrating several combinations of HPV and human transcripts were undertaken to reach 3-classes (NILM, LSIL, HSIL) and 2-classes (NILM vs HSIL) models, using either linear (elastic net) or nonlinear (and random forest) approaches, starting from 302 samples. Despite expectations, and significant differences in expression profiles between the three classes (Supplementary Data 2 and the Supplementary Appendix 1), it was not possible to reach a performant classification with 3-classes models (Table 1 and Fig. 2). In contrast, both linear and nonlinear methods produced promising 2-classes models, leading to a selection of five top models with high PPV. These models relied on different predictor variables combinations: random forest models performed better when only HPV predictors were used, while elastic net models were more performant at combining HPV and human transcripts through the S + H, uS + H, S + uS + H predictors (Table 1, Figs. 2 and 3).

### Predicting HSIL using only HPV transcripts

The two nonlinear random forest models that performed well for the detection of high-grade lesions both gave significant weight to the SD2-SA6 predictor (Imp. = 100 in Table 4 and Fig. 4). This predictor had a significant increase in expression from NILM to HSIL (Supplementary Data 2). Chen et al. (2014) described the HPV16 transcriptome from cervical clinical samples and described six different transcript species presenting the SD2-SA6 signature, namely: A, B and C with coding capacity for E6 and E7, Q corresponding to E1^E4, and the late R and S forms corresponding to E1^E4(L1), E1^E4(L2). Equivalent transcripts have been demonstrated experimentally for HPV18 (Wang et al. 2011) and predictions of equivalent positions can be made for other hrHPVs (Pérot et al. 2019). Regarding the A, B and C transcripts with coding capacity for E6 and E7, Chen et al. reported an increase in the number of fragments per kilobase of transcript per million fragments mapped (FPKM) from CIN2 [0.0–29.3 FKPM], CIN3 [2.8–39.7 FPKM] to SCC [21.4–345.9 FPKM] Chen et al. (2014), which appears in line with prior knowledge on the overexpression of E6 and E7 in the cellular transformation process (McBride and Warburton 2017; Kamal et al. 2021). The alternative transcript E1^E4 was reported to be the most abundant mRNA expressed during productive infection and is considered as a marker of basal-to-epithelial infected cells differentiation (Doorbar et al. 2005). Its quantitative variation, evaluated in the work of Chen et al., appears more balanced from CIN2 (974 FKPM), CIN3 (49 FPKM) to SCC (238 FPKM). Finally, the expression of L1 or L2 as a side product of SD2-SA6 (variants R and S) were found in CIN2 [3–63 FPKM] but not in CIN3 and SCC Chen et al. (2014). Although it is difficult to state with certainty which of these 6 mRNA variants are present or not in the samples based solely on the SD2-SA6 predictor, it seems likely that the strong increase in expression of SD2-SA6 among categories, and the identification of SD2-SA6 as the most important predictor for the two best random forest models, reflects the involvement of E6 and E7 in transforming infection.

The splice signature SD1-SA1 showed an increased expression from NILM to HSIL (Supplementary Data 2), and was also informative, albeit to a much lesser extent, for each of the two best random forest models (Imp. = 16.9 and 2.0 in Table 4 and Fig. 4). This splice event is located within the E6 Open Reading Frame (ORF), resulting in truncated versions of E6 mRNA transcript species, termed E6*, and producing E6 shortened products (Andralojc et al. 2022; Chen et al. 2014; Zheng et al. 2006). This signature is present in 3 early transcripts (B, G, L) and one late transcript with coding potential for E7(L1) (Chen et al. 2014). Due to an increased distance between E6* stop codon and E7 start codon, the E6* isoforms are thought to favor E7 translation (Chen et al. 2014). Although the association with cancer progression is still controversial, the proportion of E6* isoforms comparing with full length E6 have been reported to be higher in higher grade precancerous lesions and tumor samples compared to NILM and LSIL, suggesting that E6* isoforms might play a role in cancer development (Cerasuolo et al 2020). Of note, Andralojc et al. (2022) found E6* isoforms to be expressed in 13%, 30%, and 50–60% in NILM, LSIL and HSIL samples, respectively, which is reminiscent of our observations (Supplementary Data 2). Although they observed a tendency, the sensitivity for HSIL detection based uniquely on E6* expression was very low (Se 52%) (Andralojc et al. 2022) and they needed more data to confirm if E6* expression is related to disease progression.

Finally, the SD2-SA5 signature, which increased in expression from NILM to HSIL (Supplementary Data 2), has coding capacity for E6 or E7 (Chen et al. 2014) and was informative only in the S random forest model (Imp. = 36.3 in Table 4 and Fig. 4). The SD3-SA6 signature, which shows an increase in HSIL in the training set only (Supplementary Data 2), was informative only in the S + uS random forest model (Imp. = 6.1 in Table 4 and Fig. 4). The SD3-SA6 predictor constitutes a complex marker present in late L1 transcripts species (Chen et al. 2014; Zheng et al. 2006) but also associated with coding potential for E2C and E5 proteins. Chen et al*. *(2014) reported equivalent levels of E5 proteins in NILM and HSIL, while L1 was mostly expressed in lower grades and normal samples, and very rare in CIN3 and cancer. Taken together, these observations contribute to better delineate a fundamental understanding of the models.

### Predicting HSIL using a combination of HPV and human transcripts

Three linear elastic net models gave encouraging performances for the detection of precancerous lesions (Table 1, Figs. 2, 3). Interestingly, all three used a combination of viral and human transcriptomic signals. The S + uS + H model yielded a sensitivity of 80.0% and a specificity of 76.7%, while the uS + H model attained a sensitivity of 85.0% and a specificity of 73.3%. Predicting only with viral spliced and human transcripts (S + H) led to Se = 70.0% and Sp = 80.0% (Table 1). Andralojc et al. (2022) recently explored RNA-seq data from 15 hrHPVs (E2, E6/E7 and E6*) and 429 human genes to generate a nonlinear predictive model distinguishing NILM (no CIN) from HSIL + (CIN2 +) samples. In agreement with our findings, they found that the combined use of HPV and human data is advantageous for HSIL detection, reporting a sensitivity of 85 +−8% and specificity of 72 +—13%. In our 2-classes linear models detecting HSIL, a significant weight, shown by positive coefficients in Table 4, was given to viral markers SD2-SA6 (Imp = 99.2 in S + H) whose presumed importance in transformation has been discussed before, but also to SD2-SA9 (Imp. = 100.0 in S + uS + H) and SD2-SA7 (Imp. = 100.0 in uS + H and 53.4 in S + H) (Table 4 and Fig. 4). SD2-SA9 and SD2-SA7 transcripts had low prevalence and appear poorly correlated with other human or HPV transcripts, which indicates singular expression profiles (Supplementary Data 2). SD2-SA9 is specific to a single HPV transcript species, responsible for encoding the L1 capsid protein (Chen et al. 2014). The expression of L1 transcripts was found in higher quantities in CIN2 [82.9 FPKM] than in CIN3 and cancer samples [1.3–5.5 FPKM] in Chen et al. (2014), which is consistent with the expected reduction in the average expression level of this late gene during transformation. Nevertheless, the detection of a sporadic, possibly basal level of L1 expression in advanced lesions, which may simply reflect cell heterogeneity, translates into a significant weighting of this atypical predictor in our best elastic net models. SD2-SA7 was found only in LSIL and HSIL, but rarely in our dataset, which impedes any further discussion of its expression profile (Supplementary Data 3). On the other hand, HPV transcripts and splice markers that contributed the most to the NILM linear signature (marked by negative coefficients in Table4) included markers of both productive (SD5-SA9 Imp. = 25.5 in S + uS + H; SD3-SA6 Imp = 4.6 in S + uS + H) and transforming (SD1-SA1 Imp. = 28.0 in S + uS + H) infection. The involvement of SD1-SA1, whose expression level increases from NILM to HSIL (Supplementary Data 2), may appear to be contradictory. This, however, can be tempered by the fact that, unlike the S + uS + H, the S + H linear model did not retain the SD1-SA1 marker, as opposed to the mechanistically relevant SD2-SA6 marker which was used by both the S + uS + H and the S + H models for HSIL detection. Together with the involvement of SD3-SA6 shown previously to be a complex marker, and the non-homogeneous expression profiles observed in the training and validation sets for SD5-SA9 (Supplementary Data 2), this may point toward a lower robustness of the S + uS + H model over the S + H model.

Regarding human transcripts, features associated to HSIL included the pro-tumor genes MYC (Imp. = [5.5–42.0]), ERBB2 (Imp. = [25.6–83.4]), WNT1 (Imp. = [2.4–23.4]), HRAS (Imp. = 3.4 in S + uS + H) and TERT (Imp. = 62.7 in uS + H), the proliferation marker PCNA (Imp. = [5.6–24.3]), NOTCH1 (Imp. = [35.5–71.0]) which is involved in cell fate determination and differentiation, but also the tumor suppressor genes CDKN2A (Imp. = [26.9–33.9]), TP53 (Imp. = [6.8–9.8]), RB1 (Imp. = [0.9–8.9]) (Fig. 4). Of note, CDKN2A encodes for two tumor suppressor proteins, p16INK4A and p14ARF (Ivanov et al. 2021) and that P16 protein is already used as a progression marker in pre-cancerous lesions detection (Arip et al. 2022). Here we found CDKN2A to be associated to HSIL, similarly to authors who reported it to be upregulated in HSIL + samples (Choi et al. 2018). In addition, (Ivanov et al*.* 2021) generated predictive linear classifiers for the detection of HSIL lesions using a combination of CDKN2A gene and miRNAs, with reported sensitivity of 89% and specificity of 84%. The human regions contributing the most to the NILM molecular signature included genes known for their tumor suppressor effects (PTEN Imp. = [79.6–100]; CDH1 Imp. = [2.6–33.5]), but also pro-tumor related genes (*e.g.* cellular proliferation markers: KRAS (Imp. = [43.0–70.2]), TOP2A (Imp. = 23.4 in S + uS + H), MK167 (Imp. = 8.1 in uS + H), AKT1 (Imp. = 34.7 in uS + H), MET (Imp. = 7.6 in uS + H); and BCL2 (Imp. = [0.4–21.0]), a gene involved in the inhibition of apoptosis (Table 4, Fig. 4). Additionally, STAT1 (Imp. = [12.5–45.1]), a gene associated with immune response and cellular proliferation, also contributed to the NILM signature. Such patterns reinforce the idea of a high heterogeneity and complexity of cellular responses and a probable mix of cells harboring differentiate states of viral infection, within the same polyclonal cell sample.

### LSIL, a cytological class displaying high molecular heterogeneity

It was not possible to distinguish properly all three cytological classes (HSIL, LSIL and NILM) through a 3-classes model, due to the difficulty of predicting the intermediate LSIL category (Table 1). A posteriori, we sought to apply our two-class models (NILM vs HSIL) to LSIL samples with the underlying hypothesis that they will present a continuum of scores between NILM and HSIL. The results presented in Supplementary Figs. 10 & 11 and the Supplementary Appendix 2 showed a continuum of scores, going from zero to nearly 1, which in turn suggests a high molecular heterogeneity of LSIL samples. Several studies showed intermediary levels of various progression markers in LSIL samples (between NILM and HSIL samples), *e.g.* E6/E7 expression levels gradually higher in higher grades (*e.g.* 15% NILM, 40% ASCUS, 45% LSIL, 80% HSIL in Duvlis et al. 2015), similar levels of E6* isoforms expression in “no CIN” and LSIL samples then progressively higher in HSIL and cancer (Andralojc et al. 2022), and the productive infection marker L1 (immunoreactivity) higher in NILM and progressively lower in LSIL, HSIL and cancer (Choi et al. 2023). This limitation in defining a homogeneous LSIL category at the molecular level would therefore invite revisiting the classification criteria for low-grade lesions, currently established on morphological observations at cytological examination.

### The added value of HPV-RNA-SEQ

HPV-RNA-SEQ can be implemented using a liquid-based cytology medium, requiring no significant changes in gynecological sampling practices. We showed in a previous work that RNA remained stable in PreservCyt (Hologic) solution at room temperature up to three weeks post-sampling (Pérot et al. 2019). This time frame is compatible with grouping and carrying up a set of samples to a technical platform for NGS sequencing. In areas with limited medical or molecular biology infrastructures, this timing could also be compatible with sending self-collected samples at room temperature, provided the analytic performances of the test is not affected under these sampling conditions, which remains to be tested. While it is true that NGS-based procedures are technically more challenging than PCR-based tests, with an end-to-end turnaround time from sample processing to result taking typically between 2 and 4 days, the main benefit of HPV-RNA-SEQ lies on its potential to increase specificity, and thus the PPV for detecting precancerous lesions (Fig. 5, Supplementary Data 5) over other HPV molecular tests. Currently, when primary screening leads to the identification of hrHPV in the general population, a test with high specificity, such as cytology, is needed for the triage of women at risk of transforming infection, before colposcopy (Cuzick et al. 2013; Schneider et al. 2000). HPV-RNA-SEQ can overcome this limitation by providing information on the presence of hrHPV and classifying the risk of transformation in a single molecular procedure. Two major limitations of our study, however, are that we were not able to make a direct comparison of HPV-RNA-SEQ with conventional HPV RNA or DNA tests, and that all samples were selected to be positive for HPV DNA. These limits can be in a second step addressed as part of a clinical study dedicated to comparing performance between several tests in standard screening programs, in which HPV-negative samples, and possibly tumor samples as well, will be included. More generally, the prognostic value of the HPV-RNA-SEQ test should be considered and assessed using a prospective cohort for which the evolution towards lesions and cancer, or regression and elimination of HPV is tracked over time. If studies on larger cohorts confirm that the PPV of HPV-RNA-SEQ surpasses that of HPV RNA tests, as suggested by this work, HPV-RNA-SEQ could be recommended as a triage test and potentially replace cytology, or even serve as the primary molecular test.

## Supplementary Information

Supplementary Material 1: SuppData 1.

Supplementary Material 2: SuppData 2.

Supplementary Material 3: SuppData 3.

Supplementary Material 4: SuppData 4.

Supplementary Material 5: SuppData 5.

Supplementary Material 6: SuppTable 1.

Supplementary Material 7: SuppTable 2.

Supplementary Material 8: SuppTable 3.

Supplementary Material 9: SuppTable 4.

Supplementary Material 10: SuppTable 5.

Supplementary Material 11: SuppTable 6.

Supplementary Material 12: SupplementaryData.

## Data Availability

The datasets used and/or analyzed during the current study are available from the corresponding author on reasonable request.

## Abbreviations

HPV: Human Papillomavirus
hrHPV: High-risk Human Papillomavirus
CIN: Cervical intraepithelial neoplasia
NILM: Negative for intraepithelial lesion or malignancy
LSIL: Low-grade squamous intraepithelial lesion
HSIL: High-grade squamous intraepithelial lesion
ASCUS: Atypical squamous cells of undetermined significance
SD: Splice donor
SA: Splice acceptor
Se: Sensitivity
Sp: Specificity
PPV: Positive Predictive Value
NPV: Negative Predictive Value
