# Supplementary material for: Molecular detection of hrHPV-induced high-grade squamous intraepithelial lesions of the cervix through a targeted RNA next generation sequencing assay
Source: Mol Med. 2025 May 30;31:215. doi: 10.1186/s10020-025-01238-x (PMC12125924; doi:10.1186/s10020-025-01238-x)
Supplement: Supplementary file 1 — Supplementary Material 1: SuppData 1. [file 10020_2025_1238_MOESM1_ESM.zip › SuppData1/HPV_detection.html]

HPV RNA-Seq - HPV detection threshold


Code 

- Show All Code
- Hide All Code

# HPV RNA-Seq - HPV detection threshold

#### Analysis generated on 28/08/2023 at 16:00

- Loading data
  - Getting clinical data
  - Getting pipeline output
  - Merging
- Normalisations
  - Raw counts
  - Normalized counts
  - Counts per milion
  - Fragments per milion
- Exploring possible thresholds
  - Counts per milion
  - Fragments per milion

---

# Loading data

## Getting clinical data

## Getting pipeline output

## Merging

Some samples are missing (couldn’t find wich fastq file was associated) :

Some fastq files don’t have clinical info (couldn’t find wich sample name was associated) :

---

# Normalisations

## Raw counts

## Normalized counts

## Counts per milion

Counts / total uniquely mapped reads x 10^6

## Fragments per milion

counts / total reads (mapped + unmapped) x 10^6

---

# Exploring possible thresholds


## Counts per milion

### Classification (match exact / partiel / no match)

### Indice de dissimilarité - Jaccard

1 - number of concordant obs / (concordant obs + missing in Gynie + missing in Genotyping tests)

### ROC

## Fragments per milion

### Classification (match exact / partiel / no match)

### Indice de dissimilarité - Jaccard

1 - number of concordant obs / (concordant obs + missing in Gynie + missing in Genotyping tests)

### ROC
