# Supplementary figures and images for: Molecular detection of hrHPV-induced high-grade squamous intraepithelial lesions of the cervix through a targeted RNA next generation sequencing assay
Source: Mol Med. 2025 May 30;31:215. doi: 10.1186/s10020-025-01238-x (PMC12125924; doi:10.1186/s10020-025-01238-x)

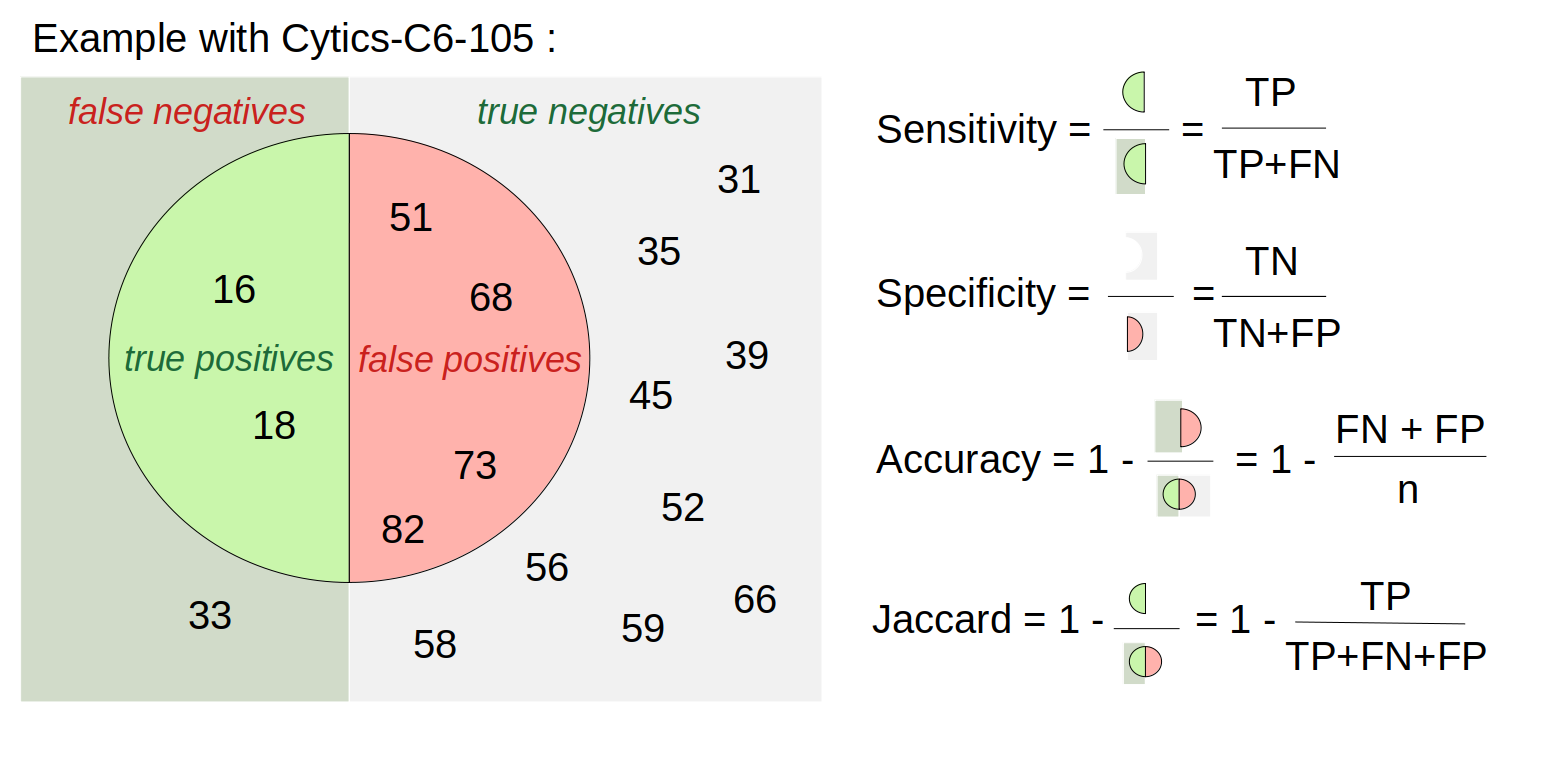

Supplement: Supplementary file 1 — Supplementary Material 1: SuppData 1. [file 10020_2025_1238_MOESM1_ESM.zip › SuppData1/Sensitivity_and_Specificity.png]

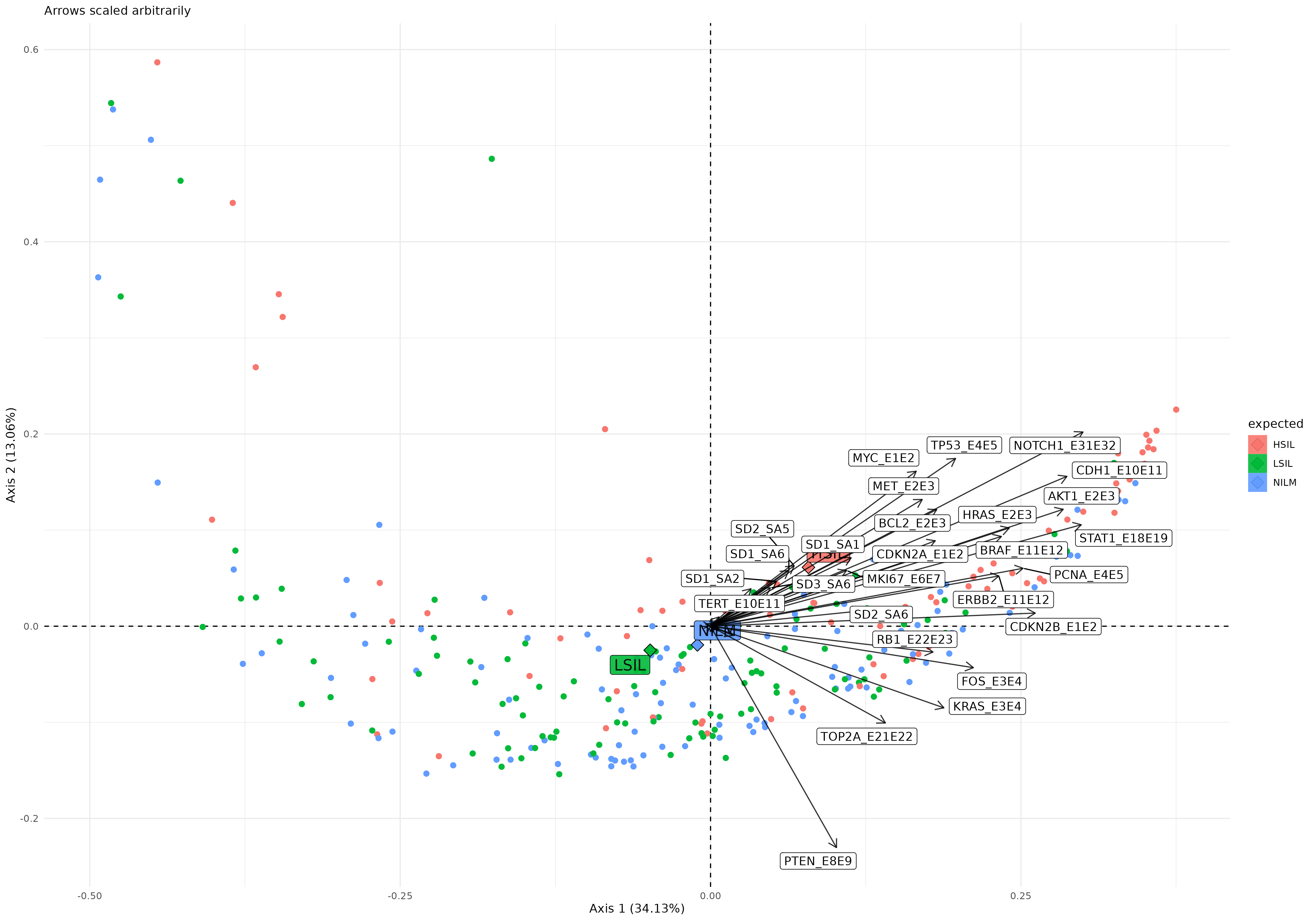

Supplement: Supplementary file 2 — Supplementary Material 2: SuppData 2. [file 10020_2025_1238_MOESM2_ESM.zip › SuppData2/PCOAs/All_PCOA_SH_elasticnet.png]

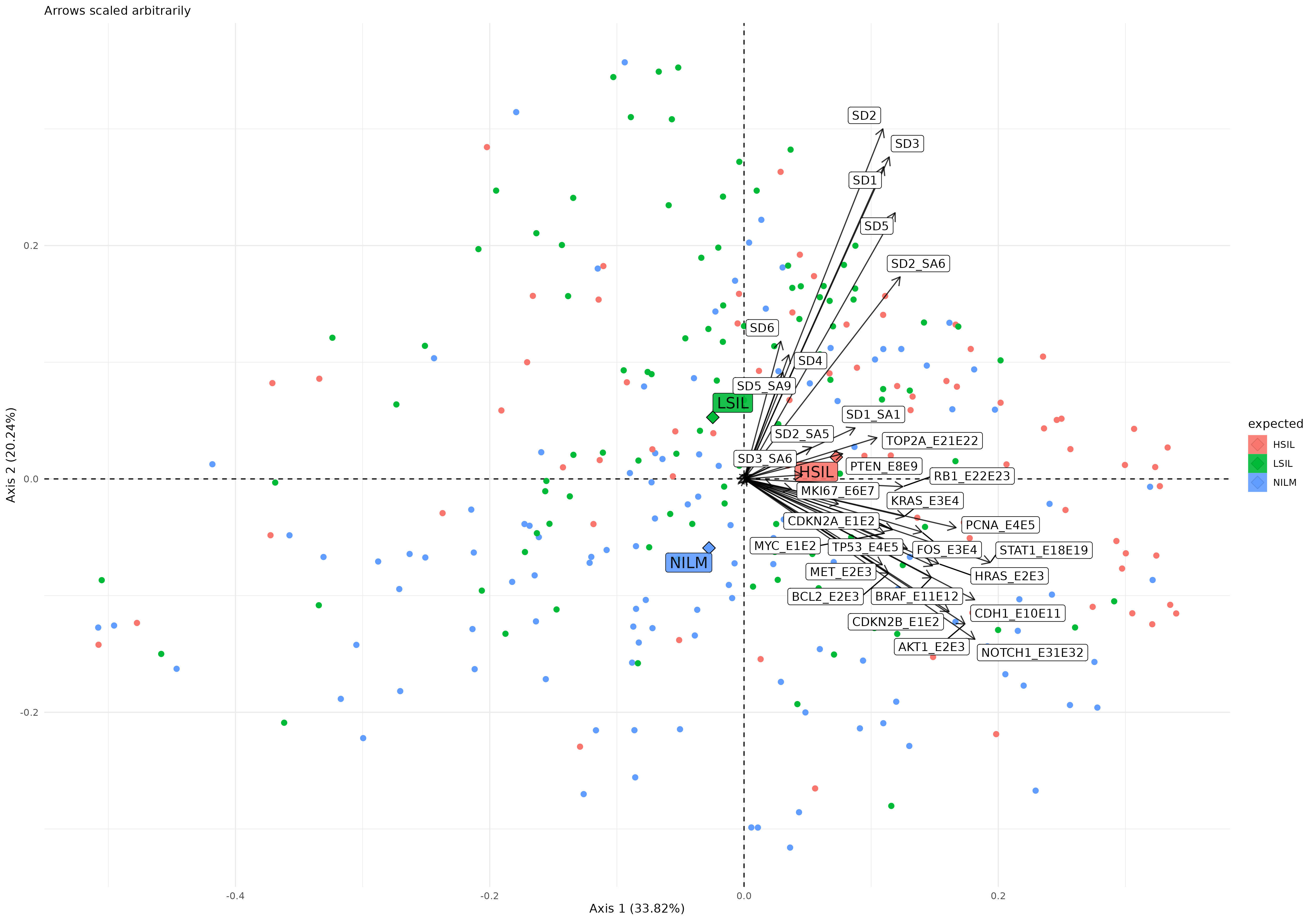

Supplement: Supplementary file 2 — Supplementary Material 2: SuppData 2. [file 10020_2025_1238_MOESM2_ESM.zip › SuppData2/PCOAs/All_PCOA_SuSH_elasticnet.png]

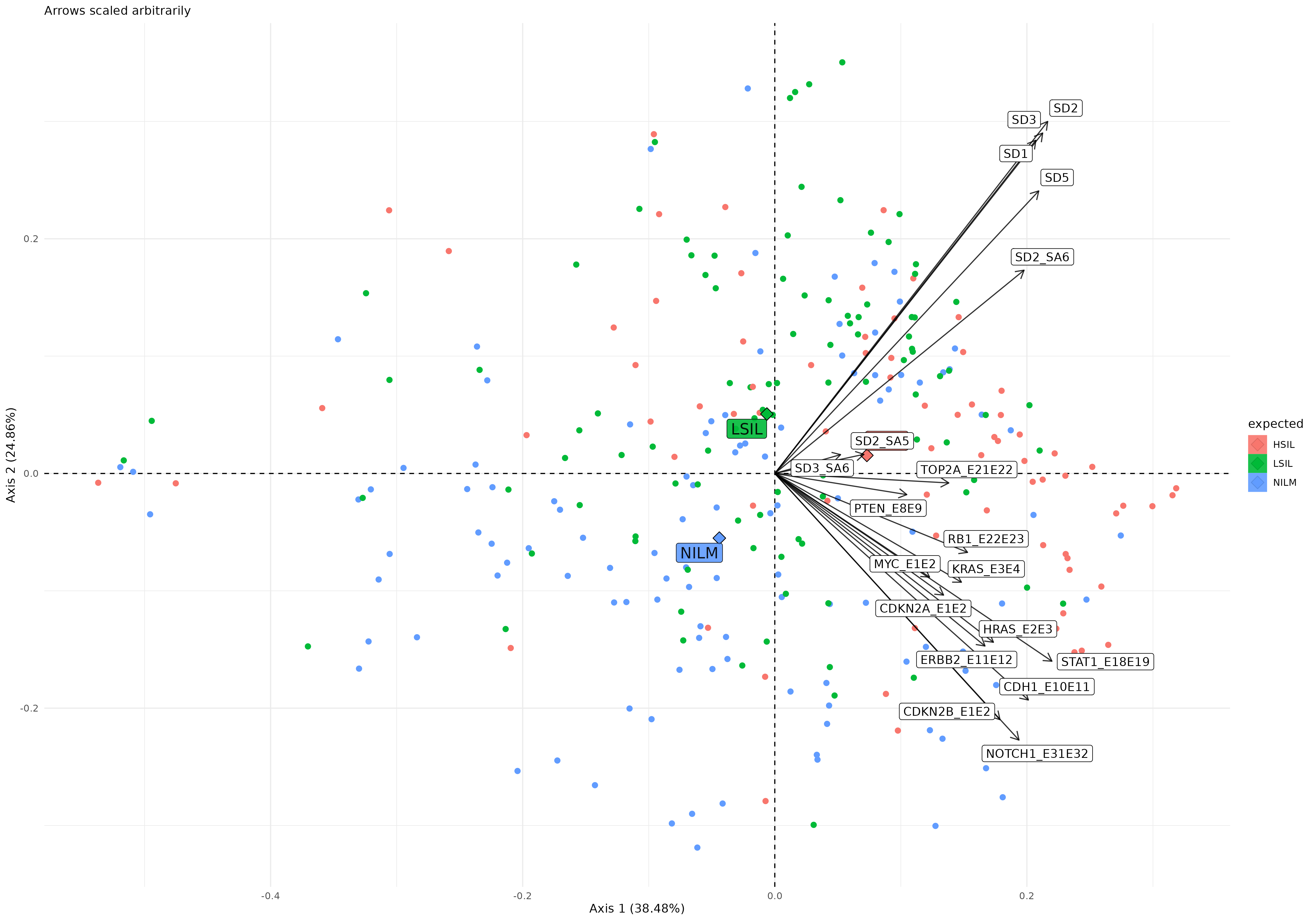

Supplement: Supplementary file 2 — Supplementary Material 2: SuppData 2. [file 10020_2025_1238_MOESM2_ESM.zip › SuppData2/PCOAs/All_PCOA_SuSH_rf.png]

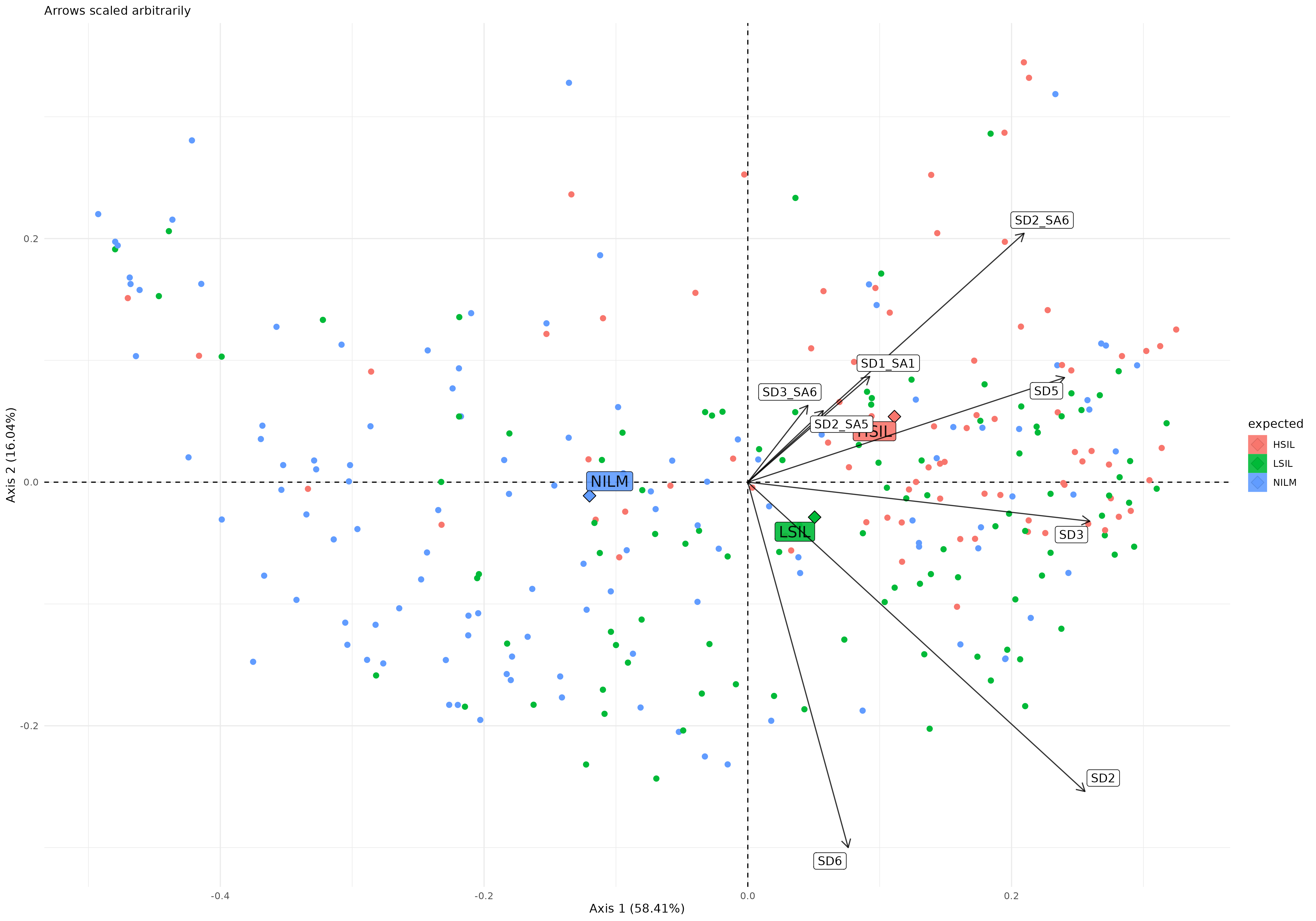

Supplement: Supplementary file 2 — Supplementary Material 2: SuppData 2. [file 10020_2025_1238_MOESM2_ESM.zip › SuppData2/PCOAs/All_PCOA_SuS_rf.png]

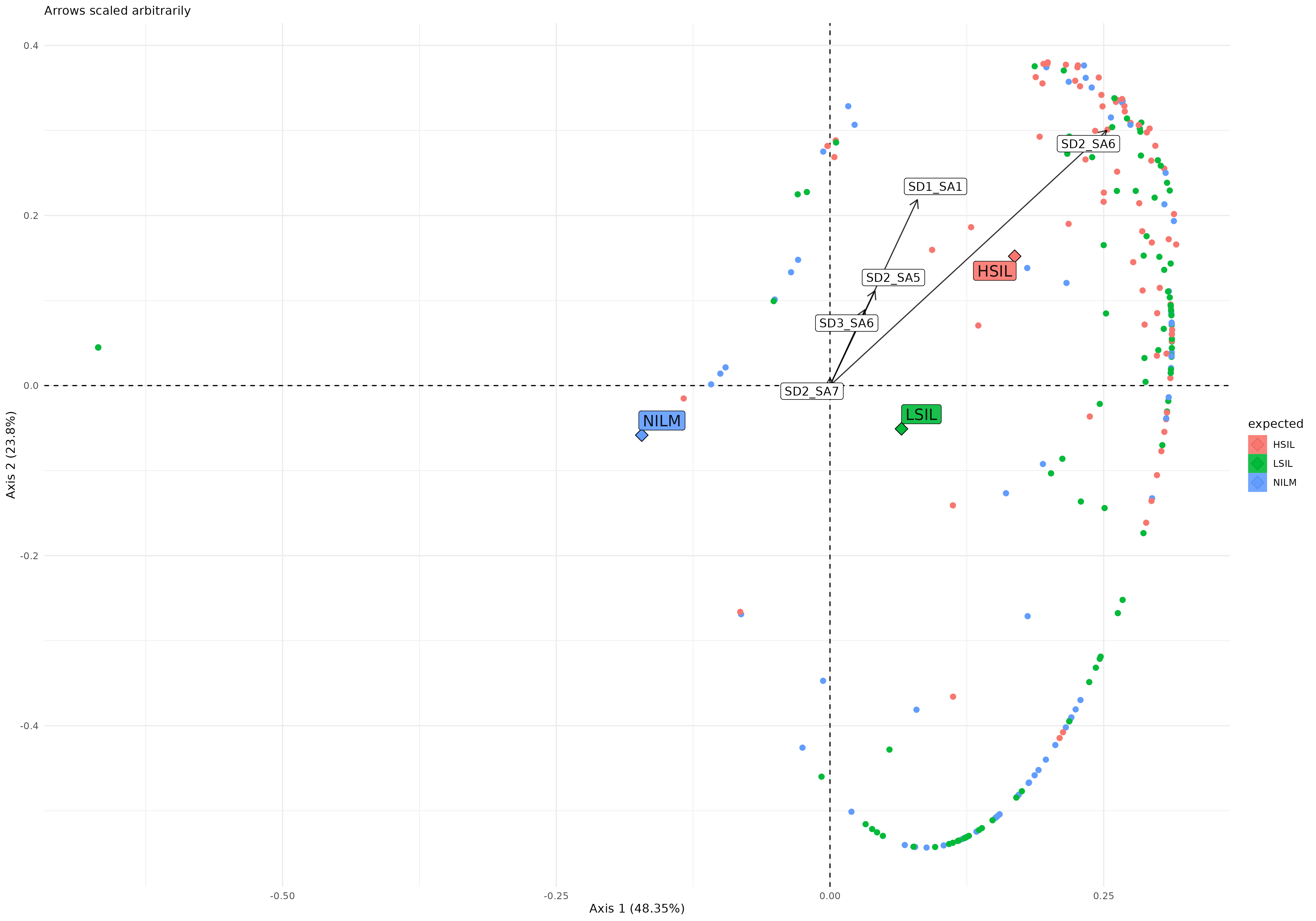

Supplement: Supplementary file 2 — Supplementary Material 2: SuppData 2. [file 10020_2025_1238_MOESM2_ESM.zip › SuppData2/PCOAs/All_PCOA_S_rf.png]

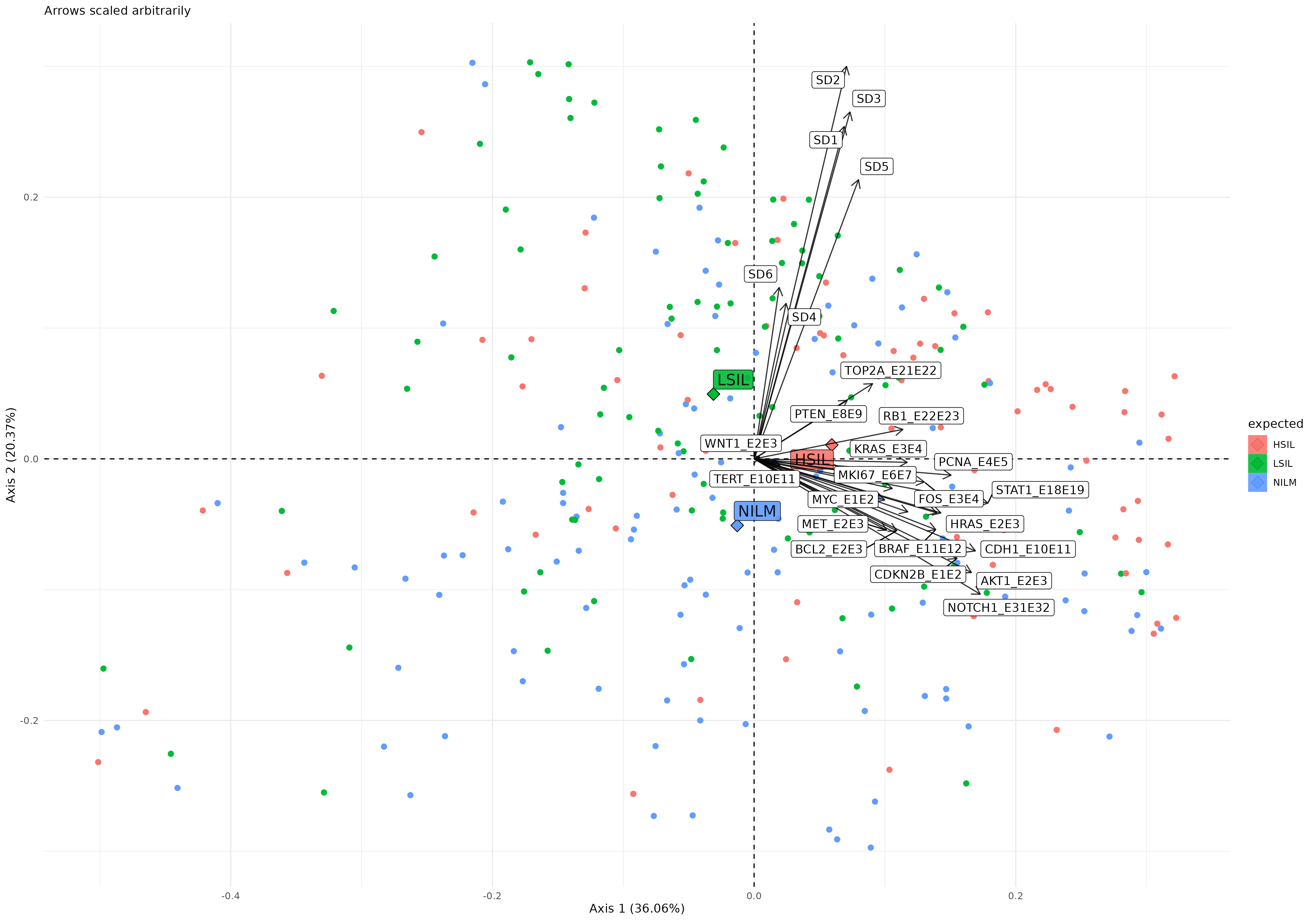

Supplement: Supplementary file 2 — Supplementary Material 2: SuppData 2. [file 10020_2025_1238_MOESM2_ESM.zip › SuppData2/PCOAs/All_PCOA_uSH_elasticnet.png]

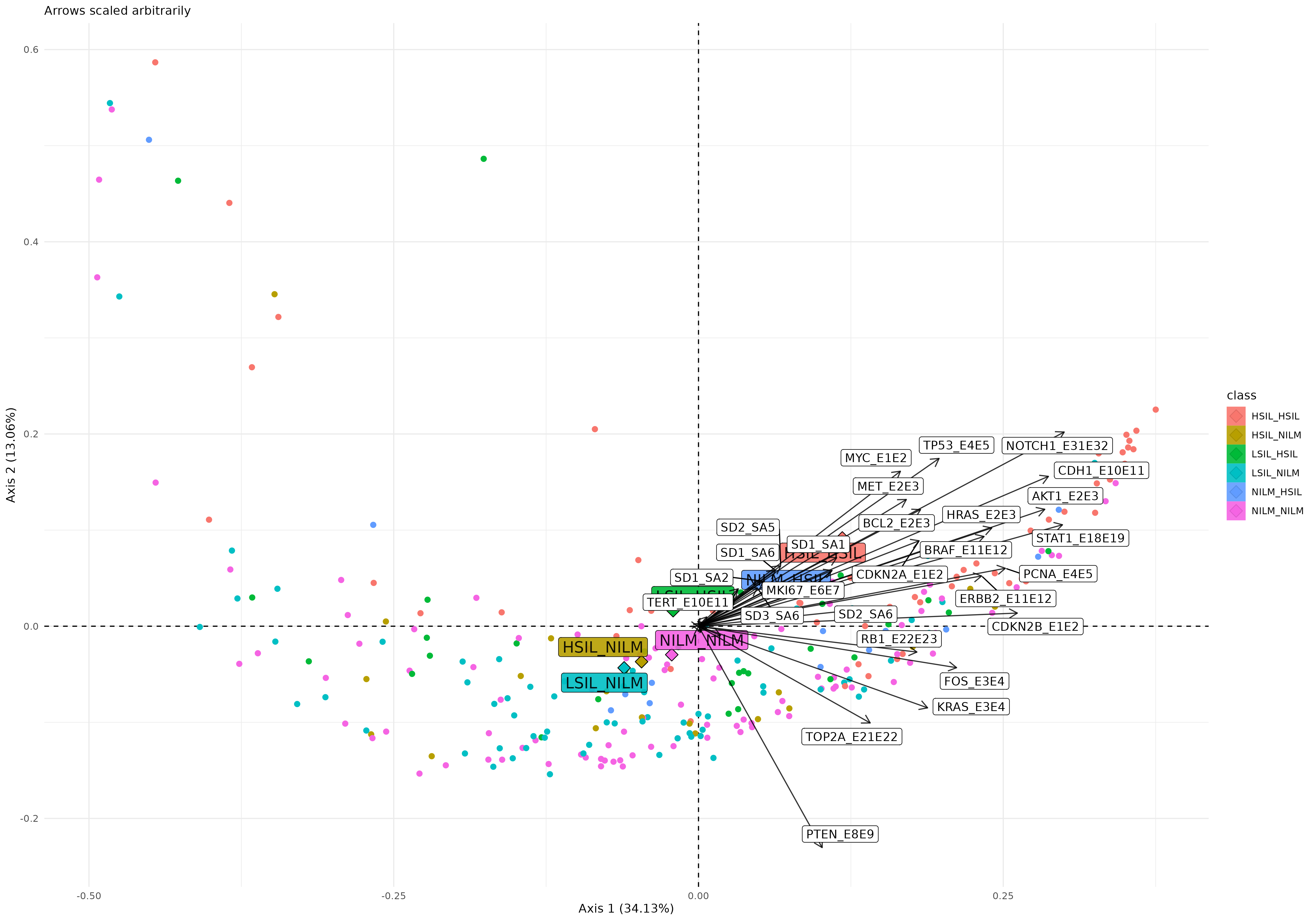

Supplement: Supplementary file 2 — Supplementary Material 2: SuppData 2. [file 10020_2025_1238_MOESM2_ESM.zip › SuppData2/PCOAs/best models/SH_en/prediction_PCOA_SH_elasticnet.png]

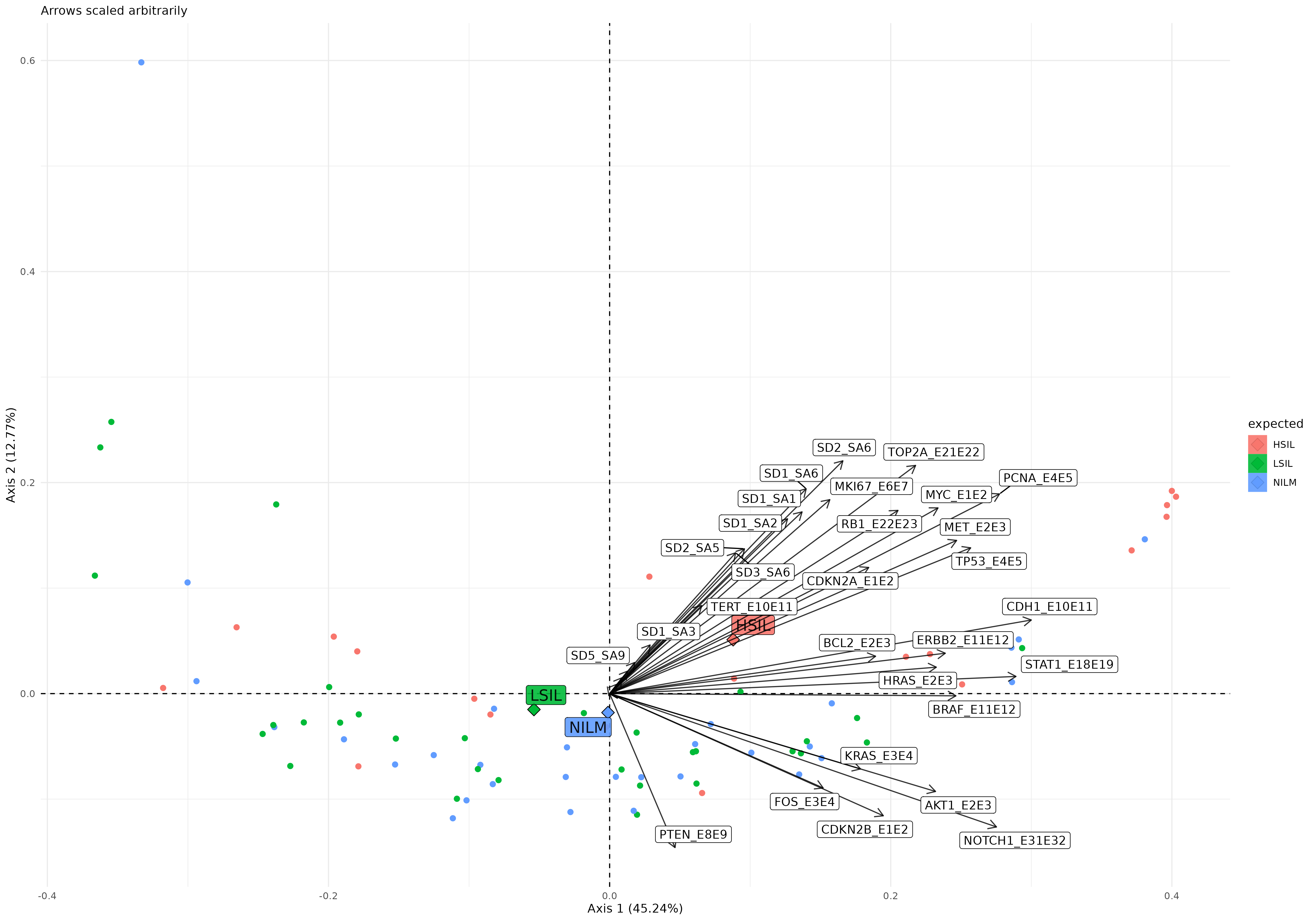

Supplement: Supplementary file 2 — Supplementary Material 2: SuppData 2. [file 10020_2025_1238_MOESM2_ESM.zip › SuppData2/PCOAs/best models/SH_en/Validation_PCOA_SH_elasticnet.png]

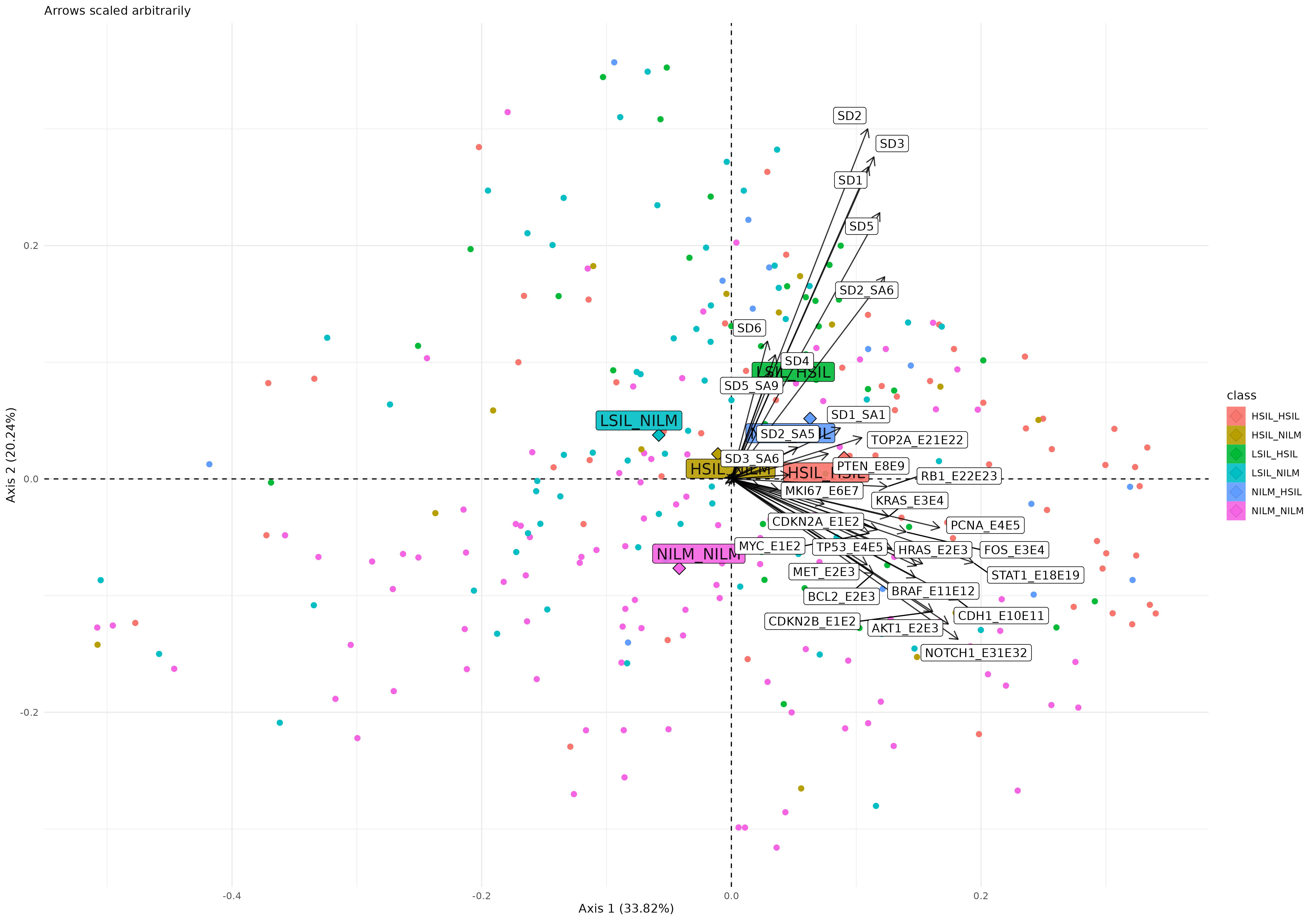

Supplement: Supplementary file 2 — Supplementary Material 2: SuppData 2. [file 10020_2025_1238_MOESM2_ESM.zip › SuppData2/PCOAs/best models/SuSH_en/prediction_PCOA_SuSH_elasticnet.png]

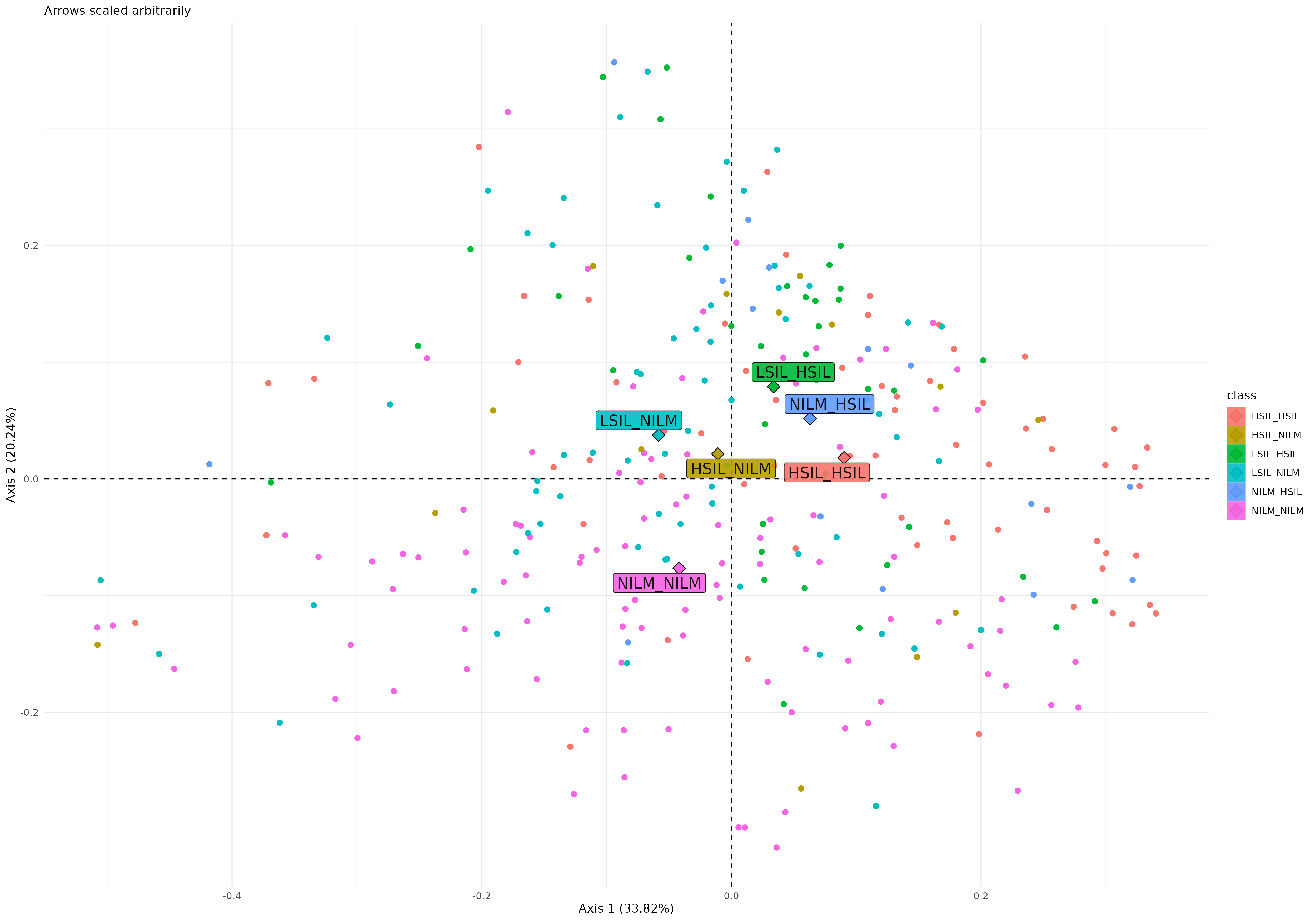

Supplement: Supplementary file 2 — Supplementary Material 2: SuppData 2. [file 10020_2025_1238_MOESM2_ESM.zip › SuppData2/PCOAs/best models/SuSH_en/sans_label_PCOA_SuSH_elasticnet.png]

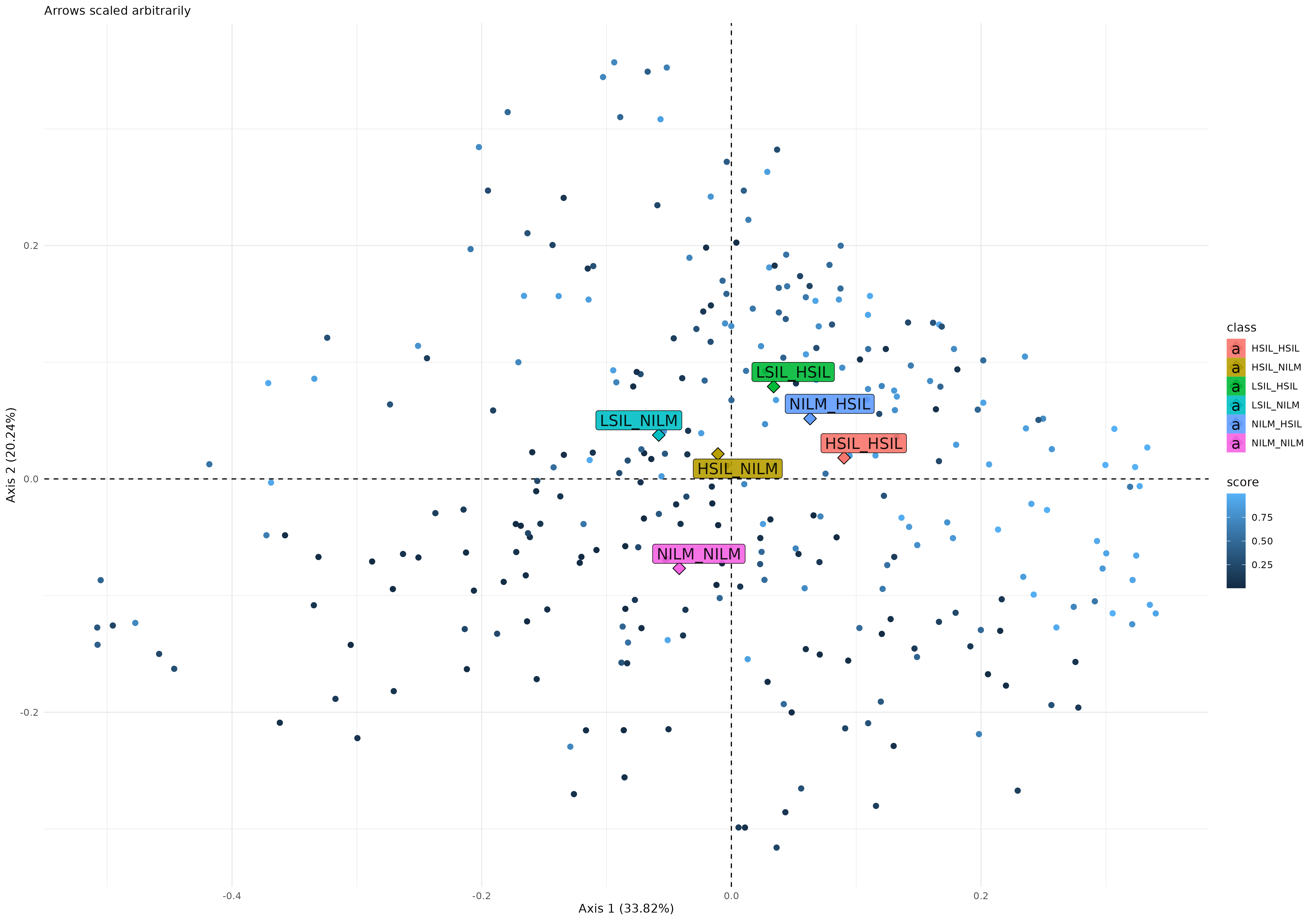

Supplement: Supplementary file 2 — Supplementary Material 2: SuppData 2. [file 10020_2025_1238_MOESM2_ESM.zip › SuppData2/PCOAs/best models/SuSH_en/score_PCOA_SuSH_elasticnet.png]

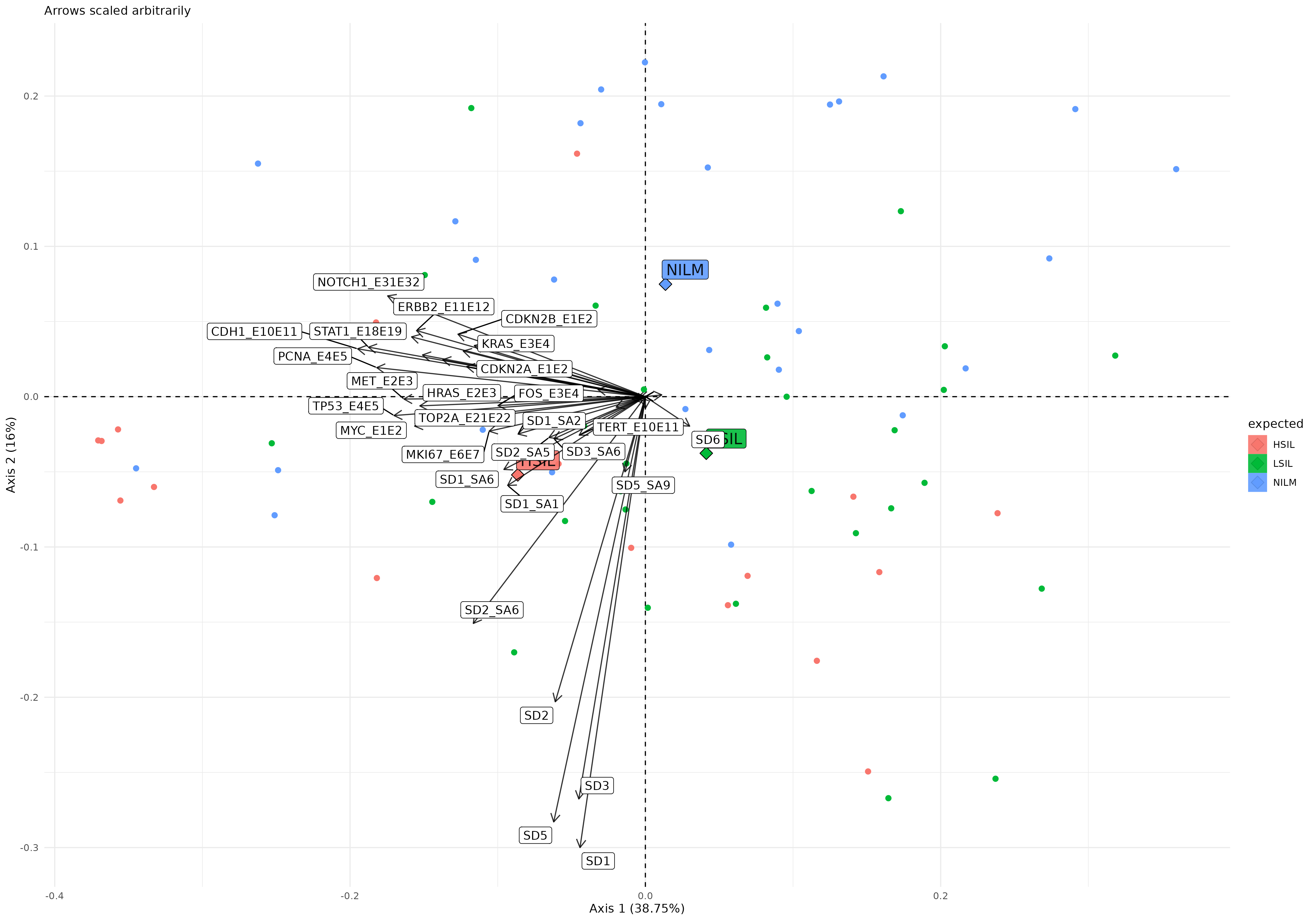

Supplement: Supplementary file 2 — Supplementary Material 2: SuppData 2. [file 10020_2025_1238_MOESM2_ESM.zip › SuppData2/PCOAs/best models/SuSH_en/Validation_PCOA_SuSH_elasticnet.png]

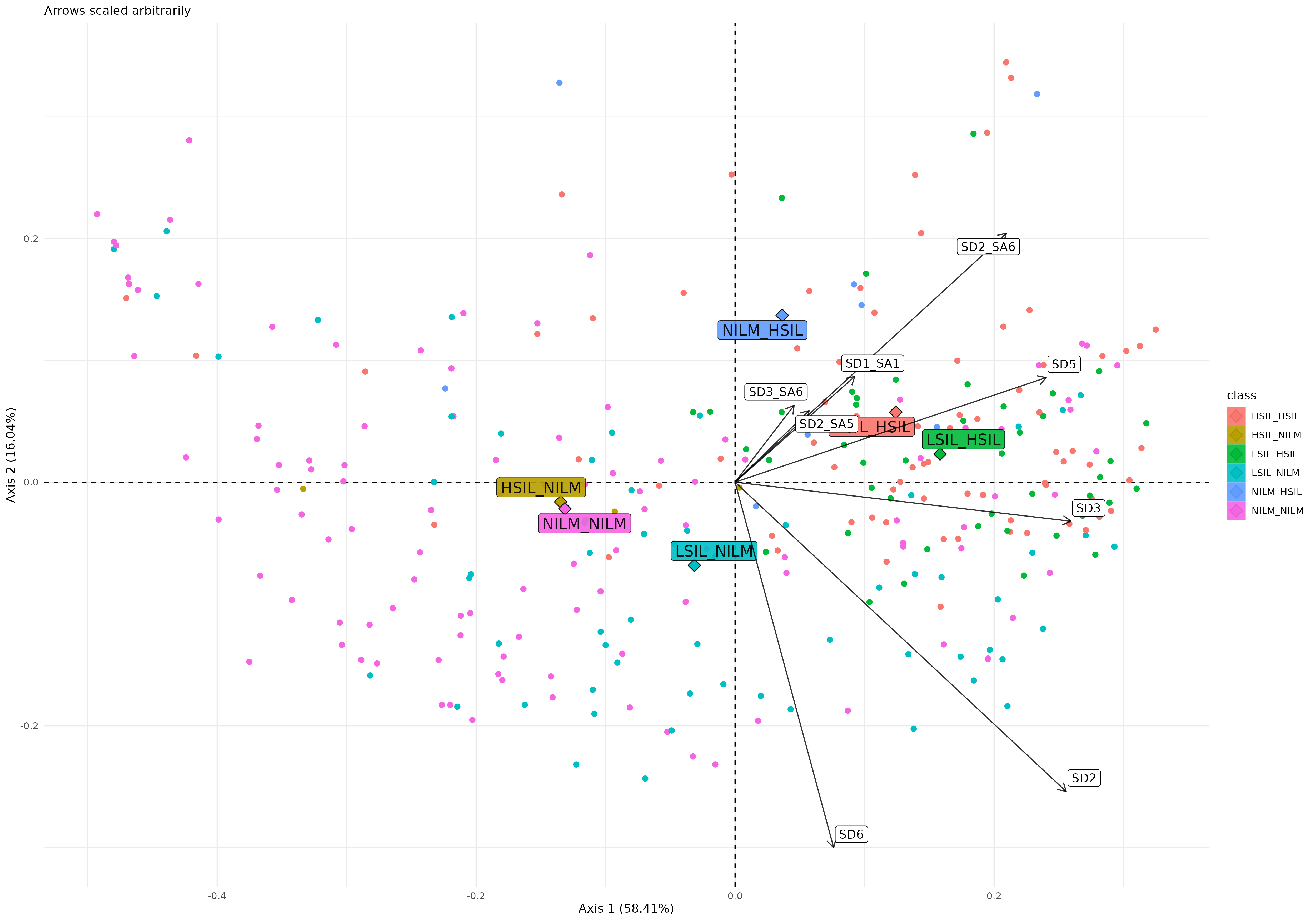

Supplement: Supplementary file 2 — Supplementary Material 2: SuppData 2. [file 10020_2025_1238_MOESM2_ESM.zip › SuppData2/PCOAs/best models/SuS_rf/prediction_PCOA_SuS_rf.png]

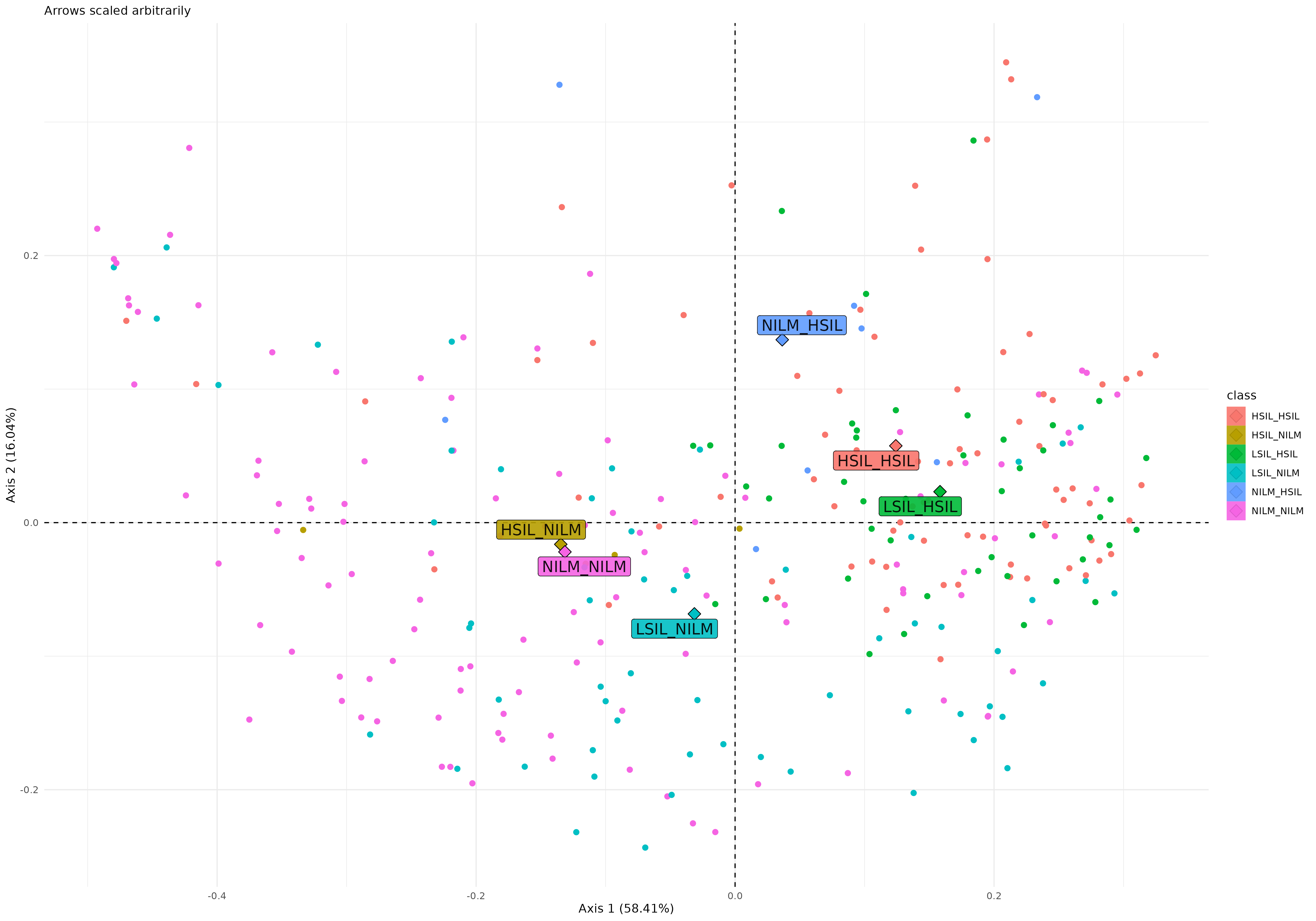

Supplement: Supplementary file 2 — Supplementary Material 2: SuppData 2. [file 10020_2025_1238_MOESM2_ESM.zip › SuppData2/PCOAs/best models/SuS_rf/sans_label_PCOA_SuS_rf.png]

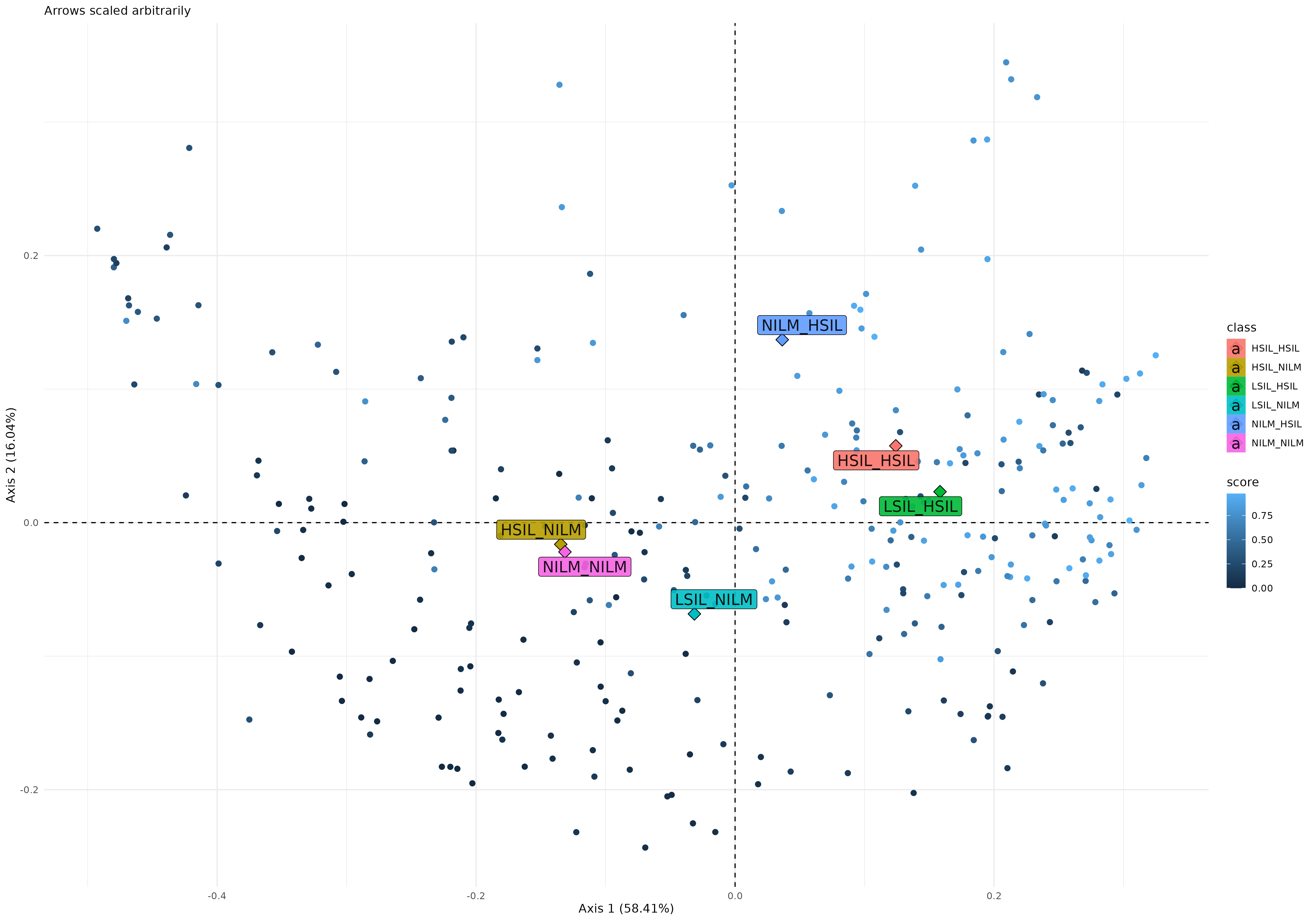

Supplement: Supplementary file 2 — Supplementary Material 2: SuppData 2. [file 10020_2025_1238_MOESM2_ESM.zip › SuppData2/PCOAs/best models/SuS_rf/score_PCOA_SuS_rf.png]

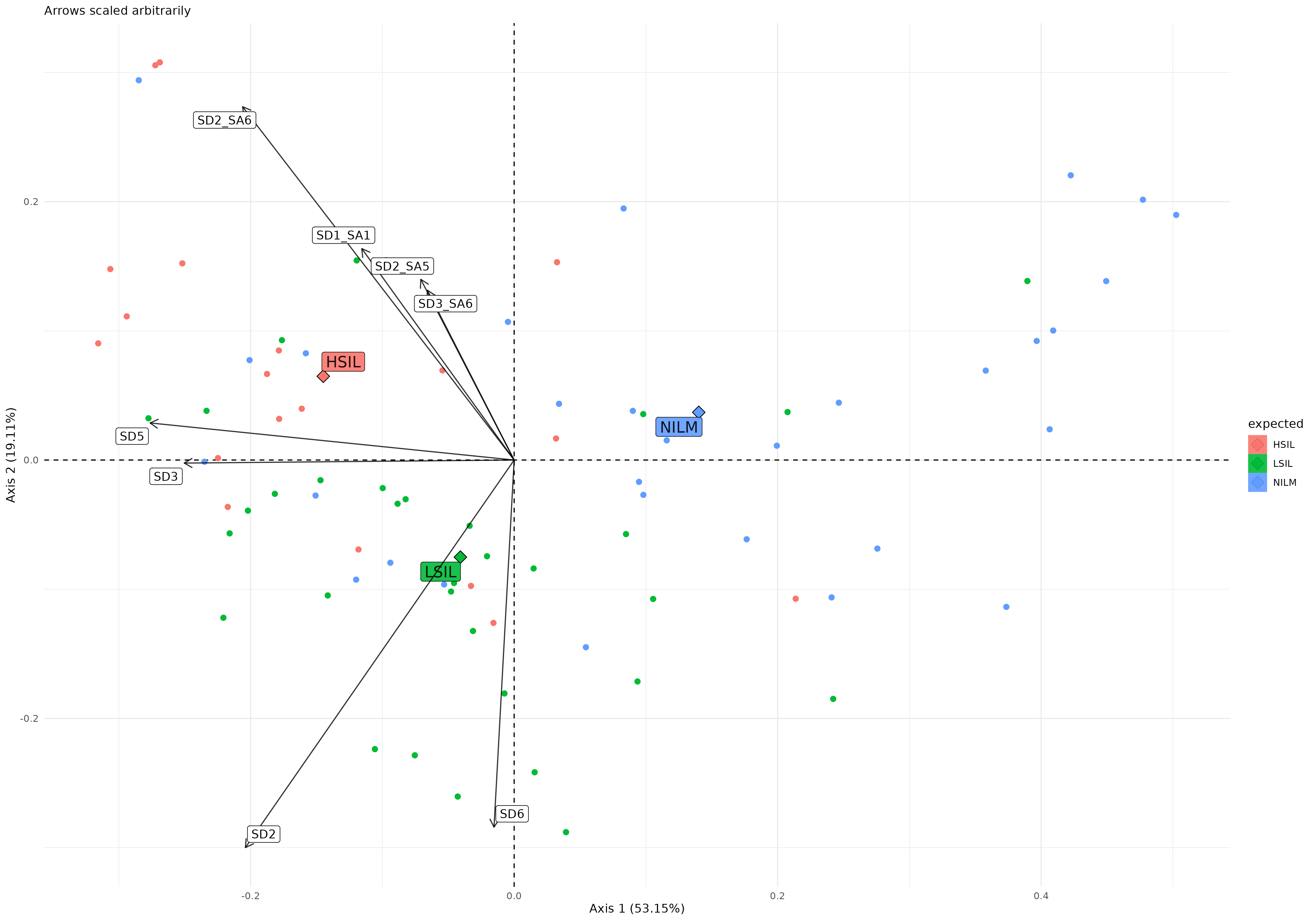

Supplement: Supplementary file 2 — Supplementary Material 2: SuppData 2. [file 10020_2025_1238_MOESM2_ESM.zip › SuppData2/PCOAs/best models/SuS_rf/Validation_PCOA_SuS_rf.png]

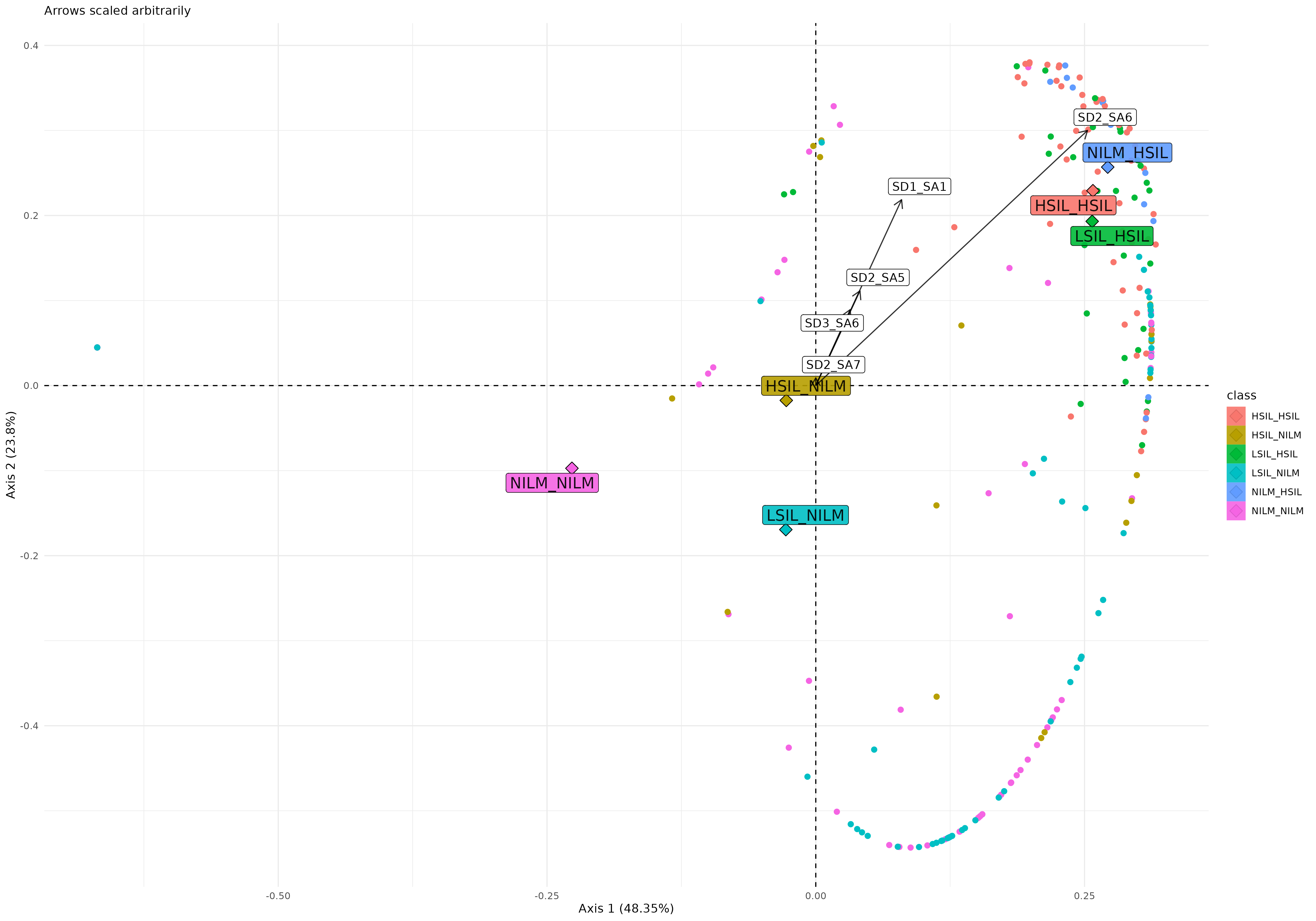

Supplement: Supplementary file 2 — Supplementary Material 2: SuppData 2. [file 10020_2025_1238_MOESM2_ESM.zip › SuppData2/PCOAs/best models/S_rf/prediction_PCOA_S_rf.png]

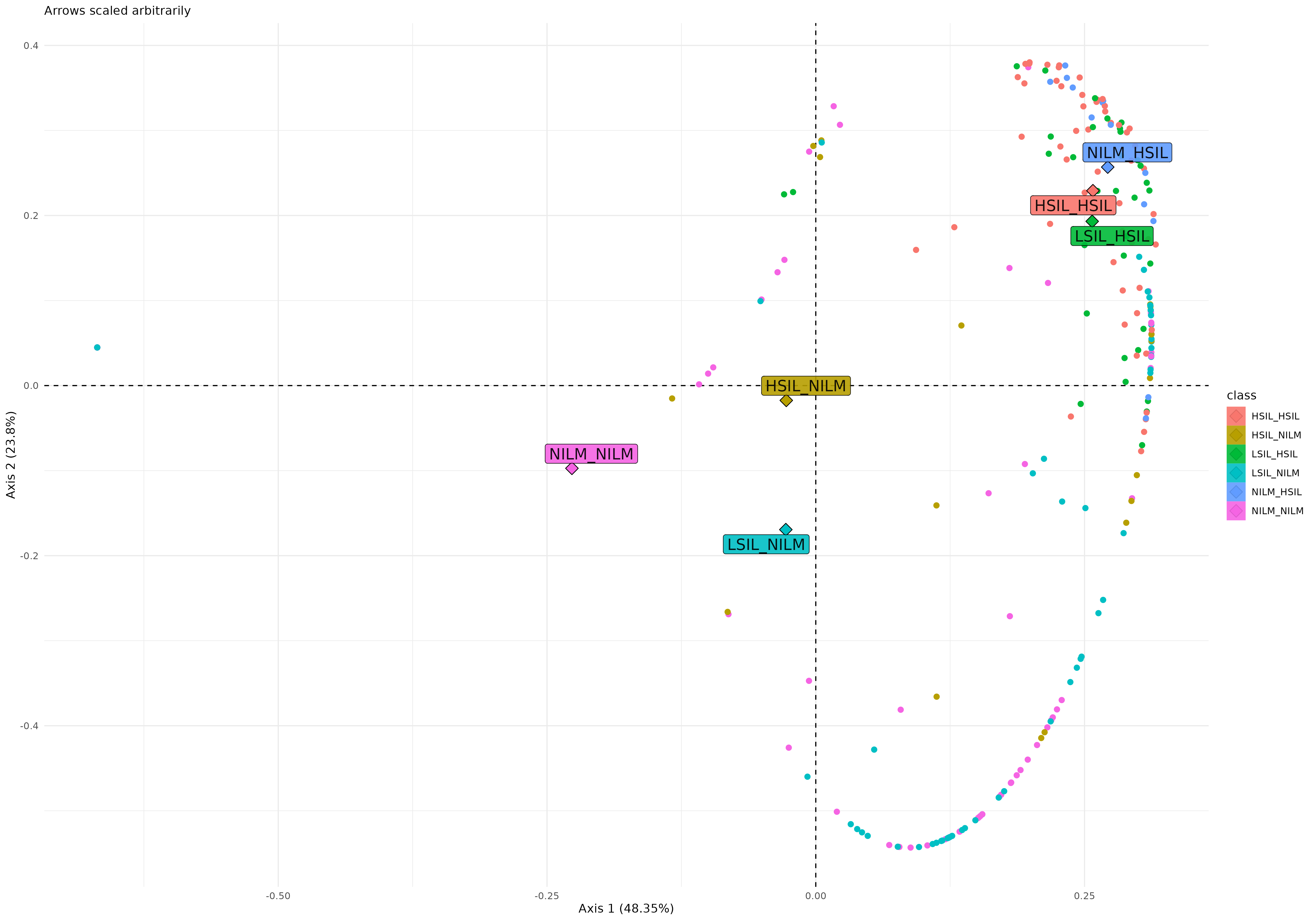

Supplement: Supplementary file 2 — Supplementary Material 2: SuppData 2. [file 10020_2025_1238_MOESM2_ESM.zip › SuppData2/PCOAs/best models/S_rf/sans_label_PCOA_S_rf.png]

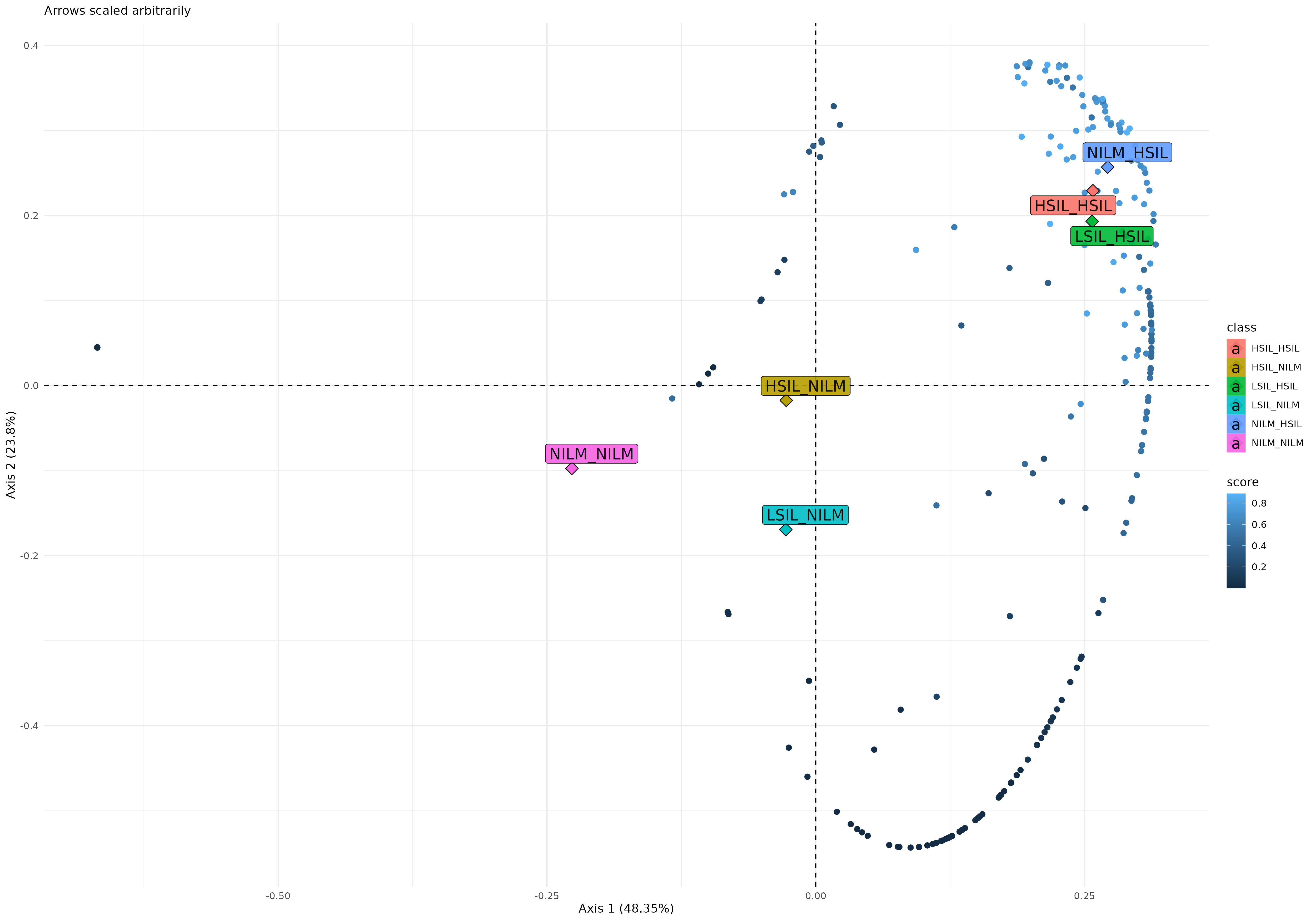

Supplement: Supplementary file 2 — Supplementary Material 2: SuppData 2. [file 10020_2025_1238_MOESM2_ESM.zip › SuppData2/PCOAs/best models/S_rf/score_PCOA_S_rf.png]

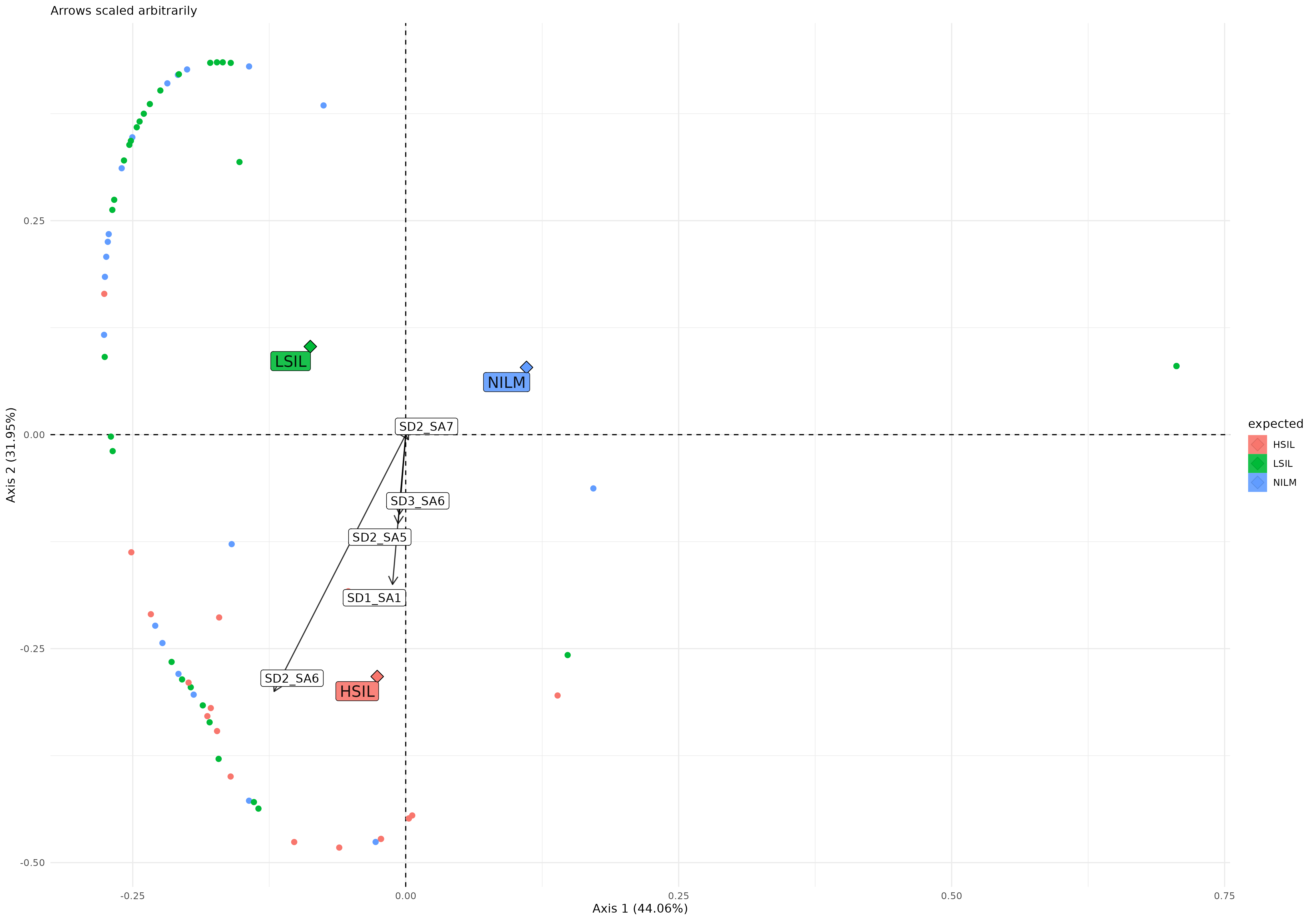

Supplement: Supplementary file 2 — Supplementary Material 2: SuppData 2. [file 10020_2025_1238_MOESM2_ESM.zip › SuppData2/PCOAs/best models/S_rf/Validation_PCOA_S_rf.png]

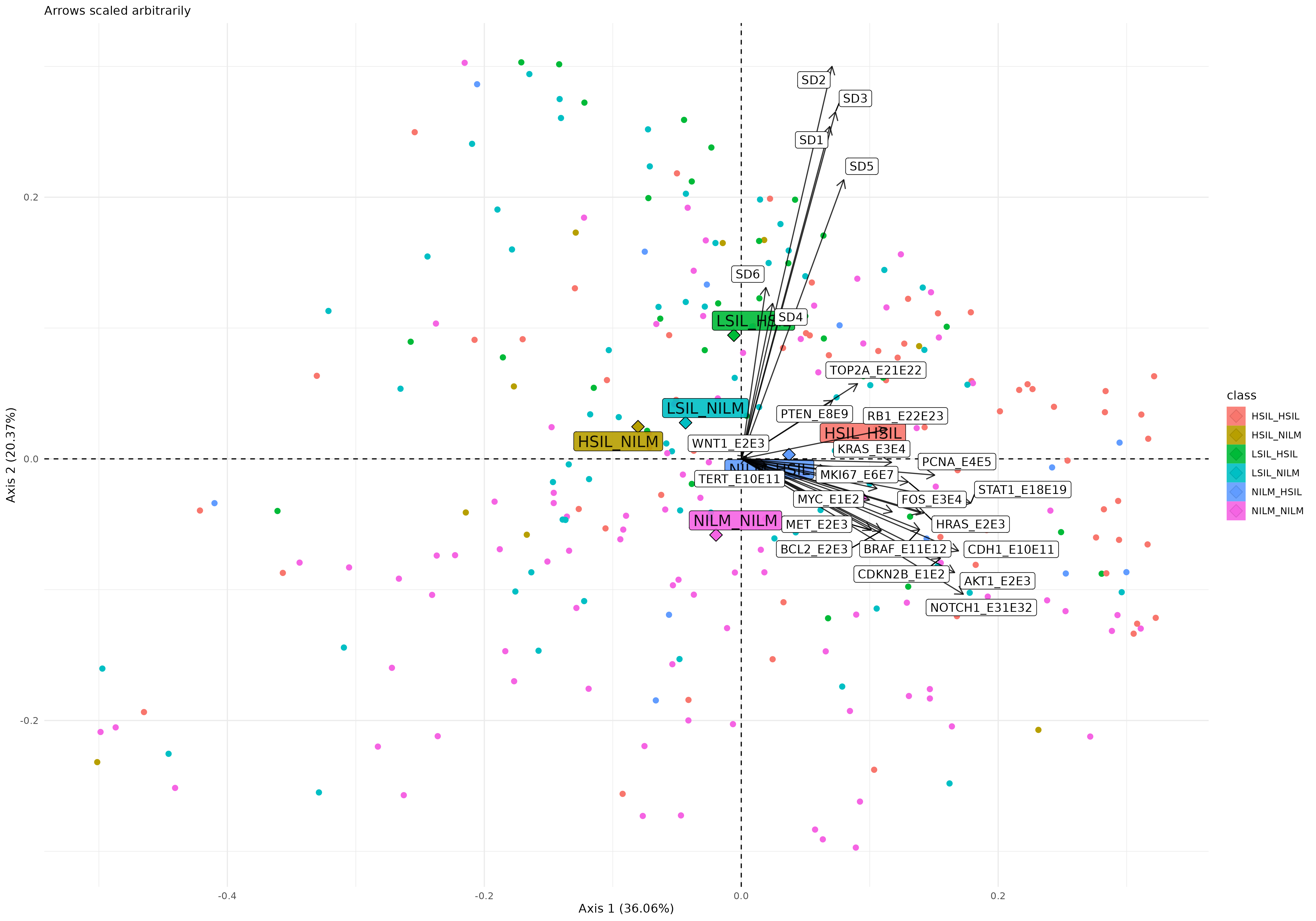

Supplement: Supplementary file 2 — Supplementary Material 2: SuppData 2. [file 10020_2025_1238_MOESM2_ESM.zip › SuppData2/PCOAs/best models/uSH_en/prediction_PCOA_uSH_elasticnet.png]

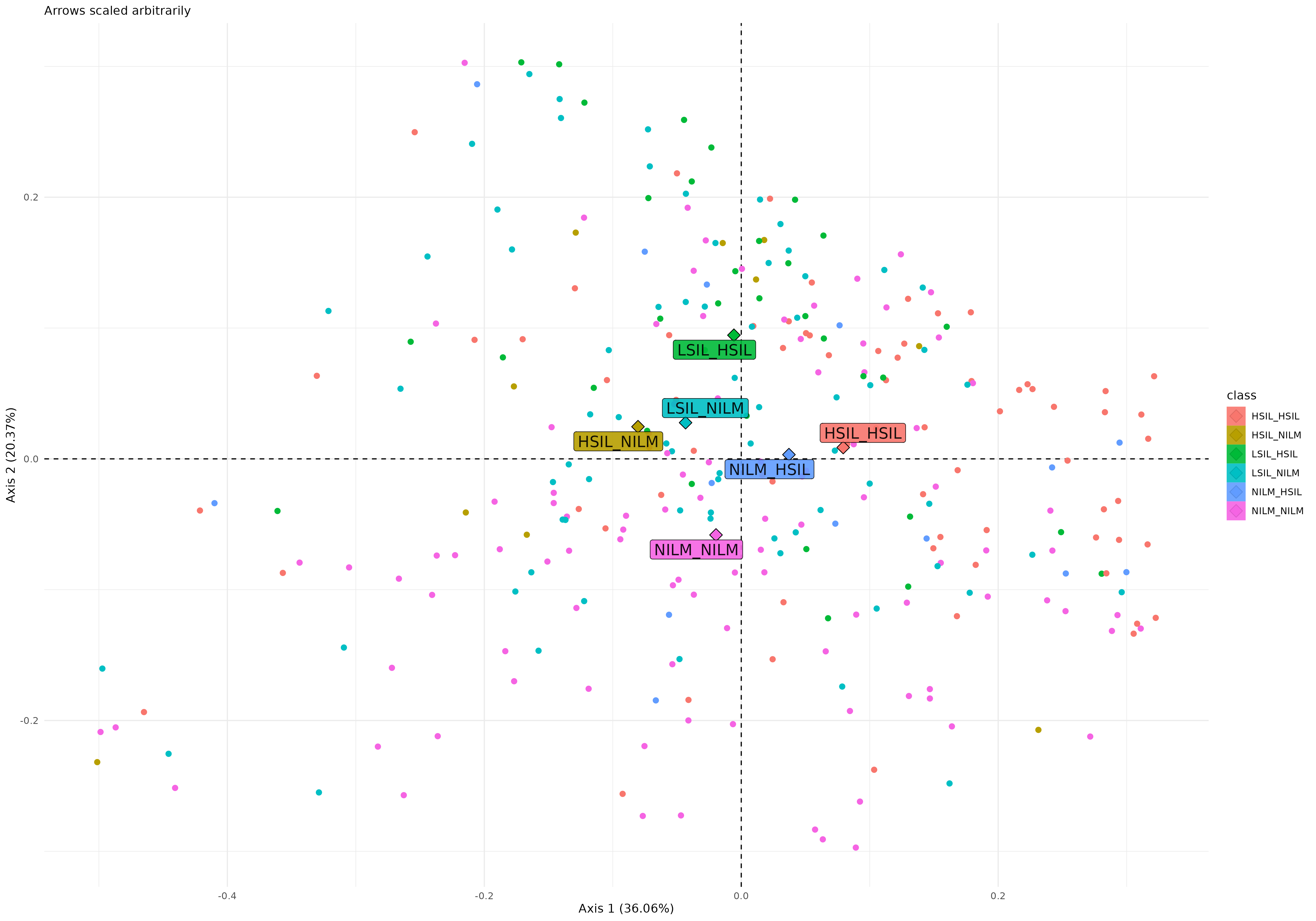

Supplement: Supplementary file 2 — Supplementary Material 2: SuppData 2. [file 10020_2025_1238_MOESM2_ESM.zip › SuppData2/PCOAs/best models/uSH_en/sans_label_PCOA_uSH_elasticnet.png]

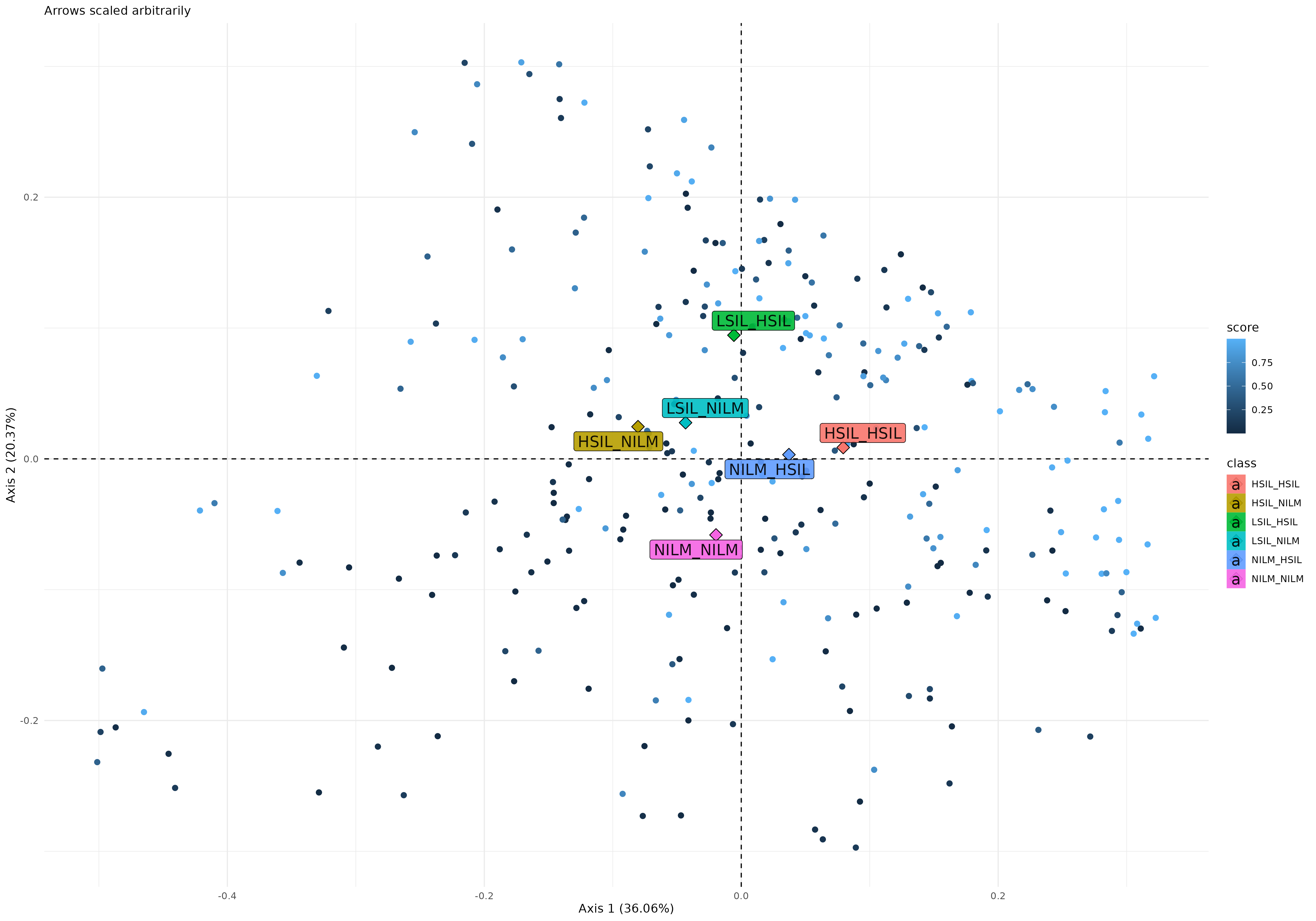

Supplement: Supplementary file 2 — Supplementary Material 2: SuppData 2. [file 10020_2025_1238_MOESM2_ESM.zip › SuppData2/PCOAs/best models/uSH_en/score_PCOA_uSH_elasticnet.png]

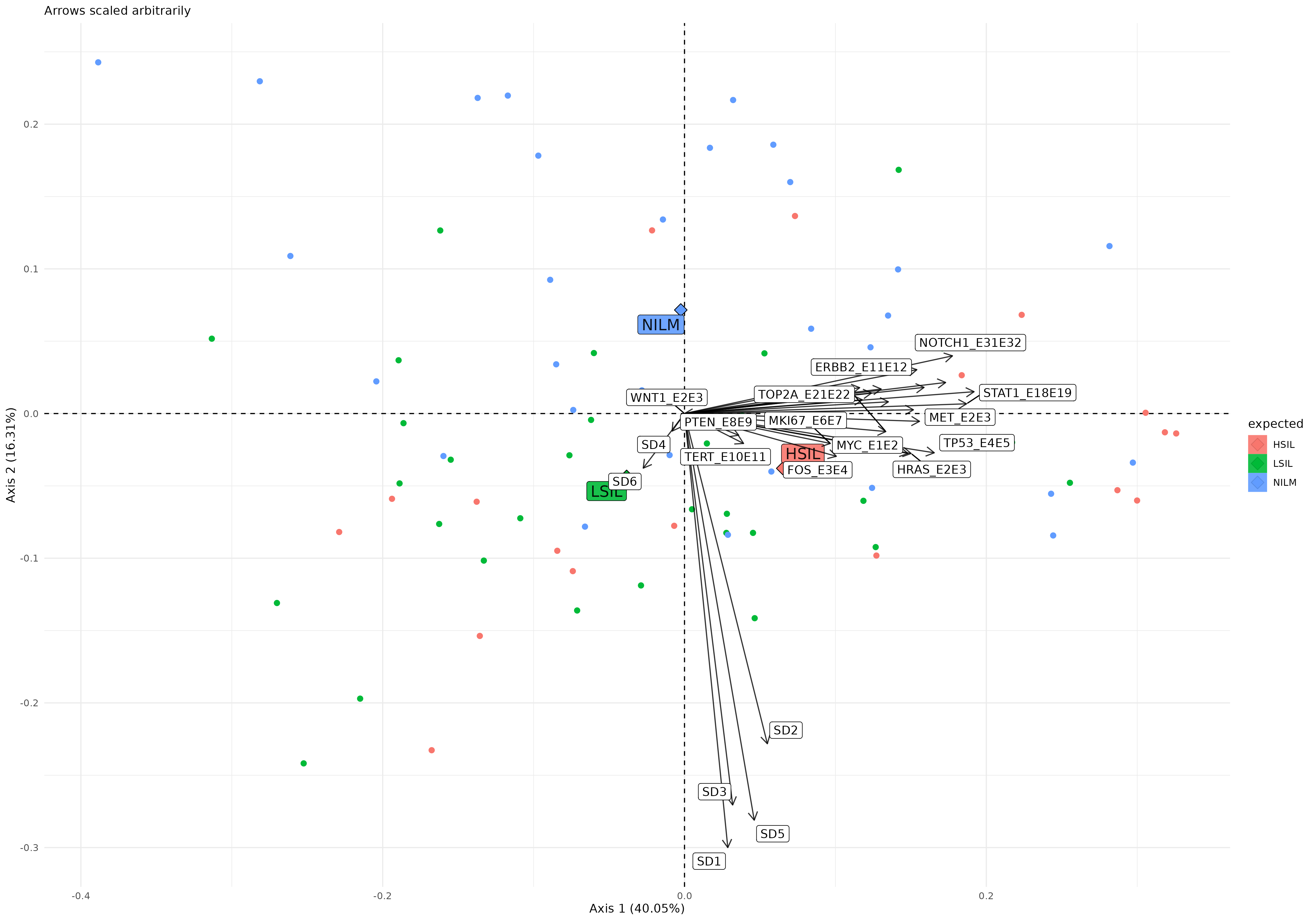

Supplement: Supplementary file 2 — Supplementary Material 2: SuppData 2. [file 10020_2025_1238_MOESM2_ESM.zip › SuppData2/PCOAs/best models/uSH_en/Validation_PCOA_uSH_elasticnet.PNG]

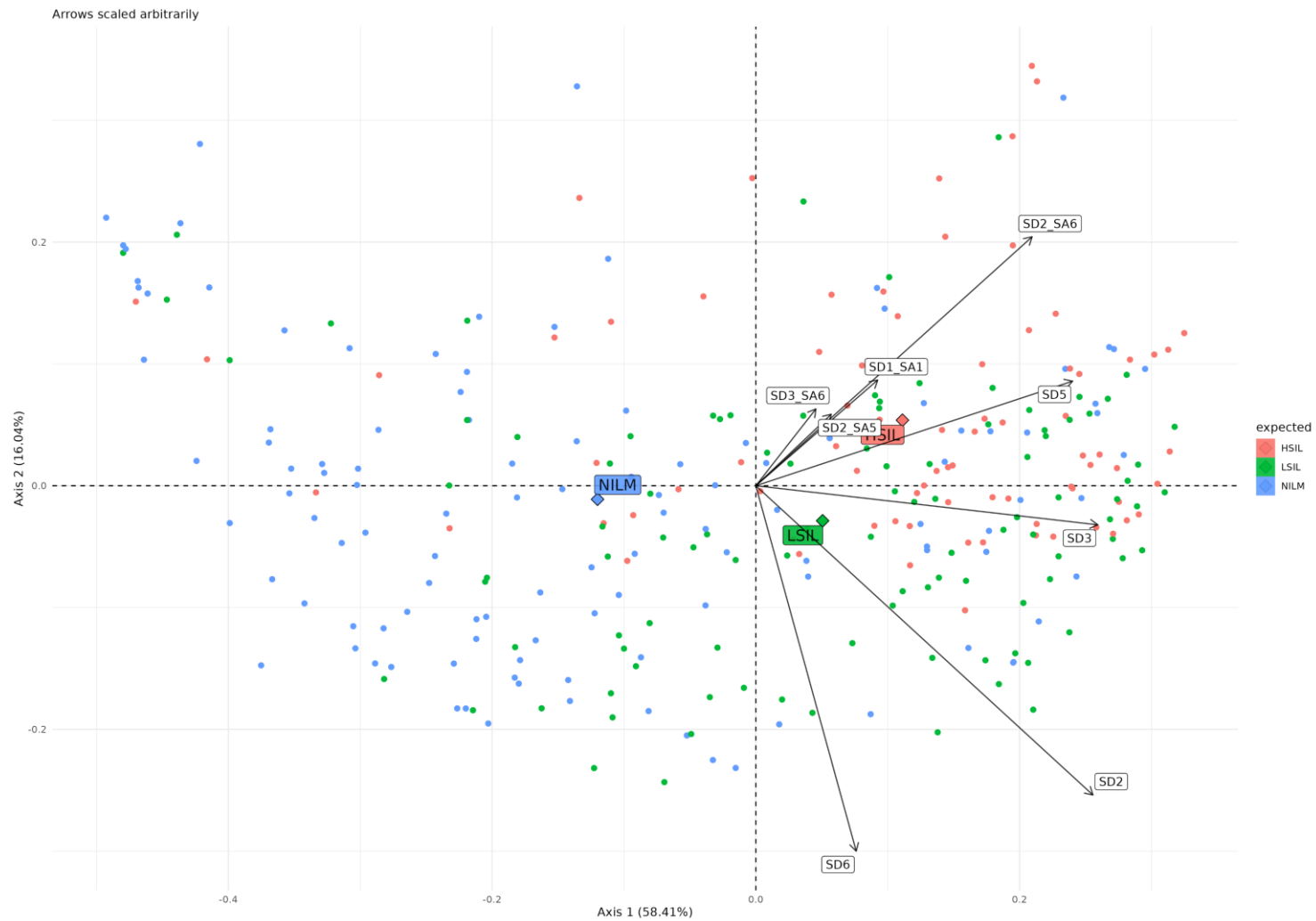

**PCOA example:** PCOA on transcriptomic data; SuS model and all samples.

Supplement: Supplementary file 2 — Supplementary Material 2: SuppData 2. [file 10020_2025_1238_MOESM2_ESM.zip › SuppData2/PCOAs/example PCOA - SuS model on all samples.pdf]

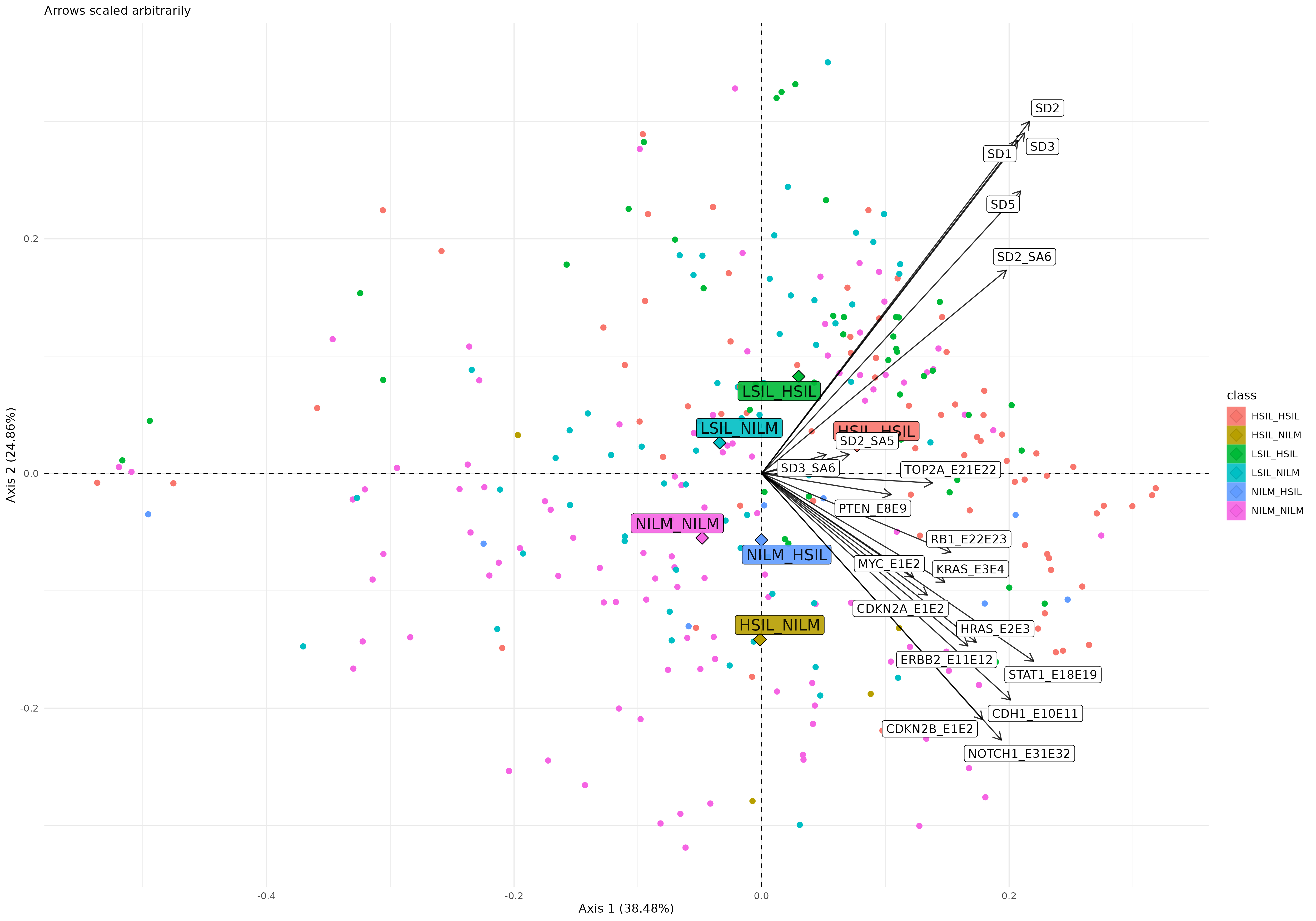

Supplement: Supplementary file 2 — Supplementary Material 2: SuppData 2. [file 10020_2025_1238_MOESM2_ESM.zip › SuppData2/PCOAs/prediction_PCOA_SuSH_rf.png]

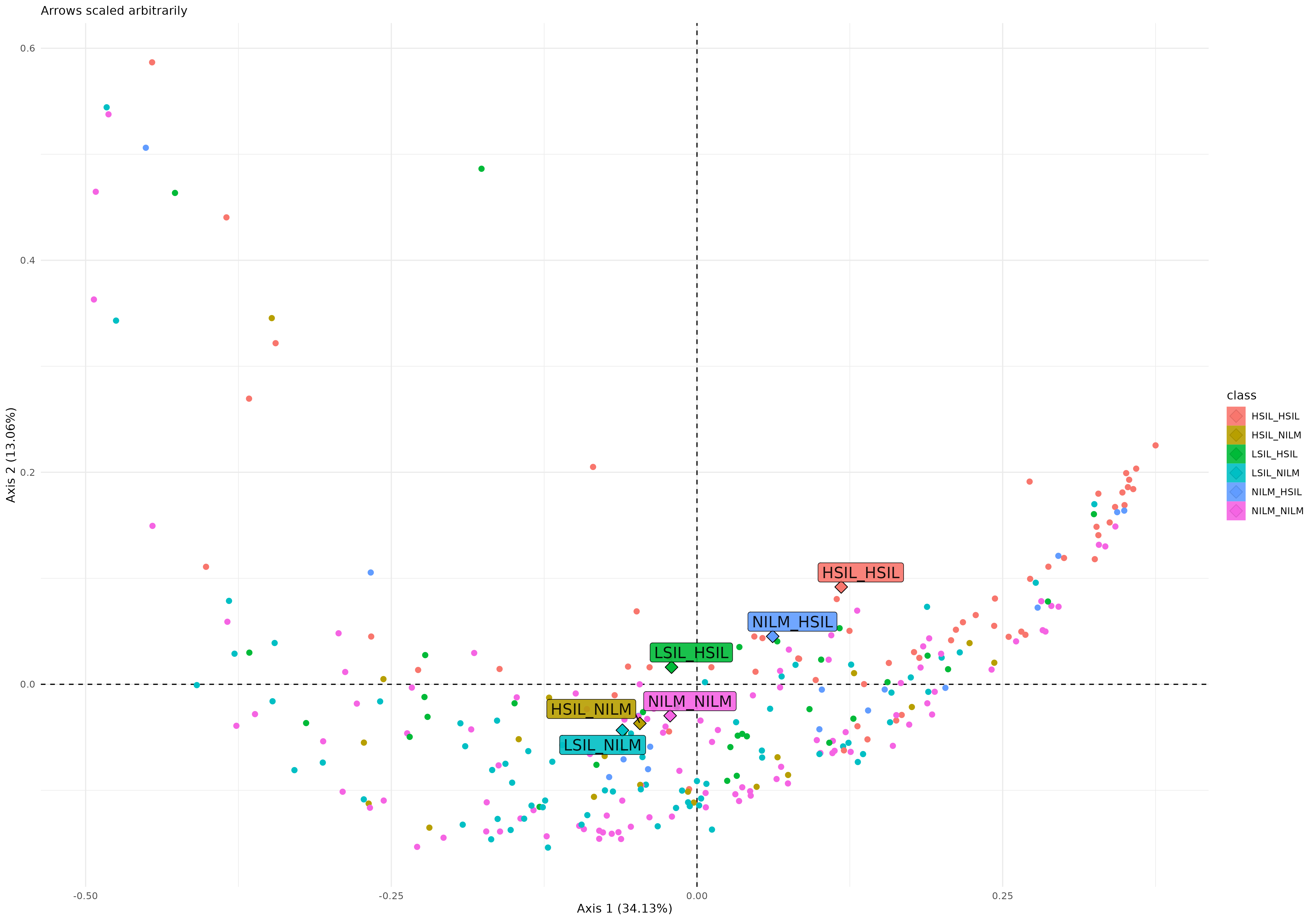

Supplement: Supplementary file 2 — Supplementary Material 2: SuppData 2. [file 10020_2025_1238_MOESM2_ESM.zip › SuppData2/PCOAs/sans_label_PCOA_SH_elasticnet.png]

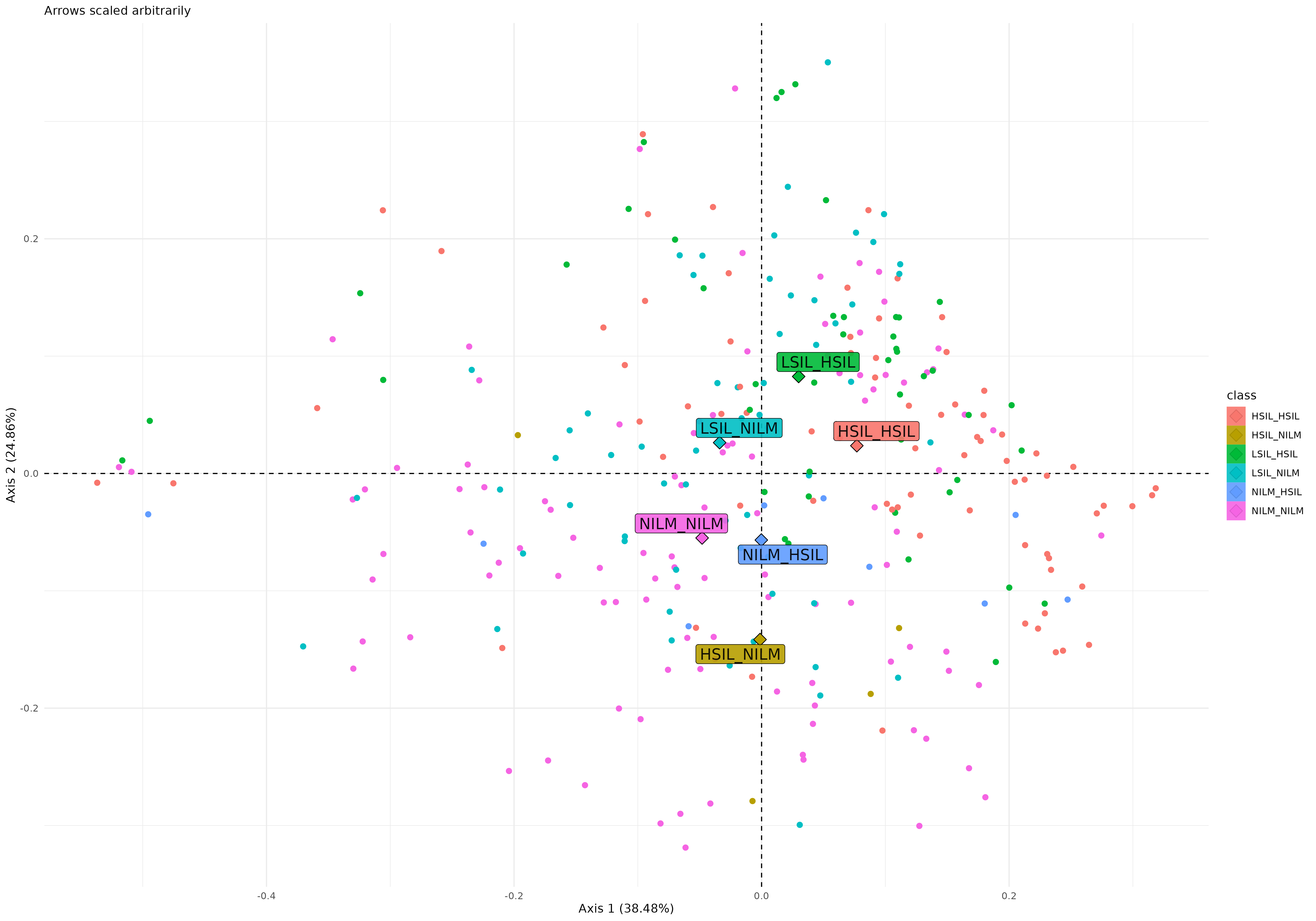

Supplement: Supplementary file 2 — Supplementary Material 2: SuppData 2. [file 10020_2025_1238_MOESM2_ESM.zip › SuppData2/PCOAs/sans_label_PCOA_SuSH_rf.png]

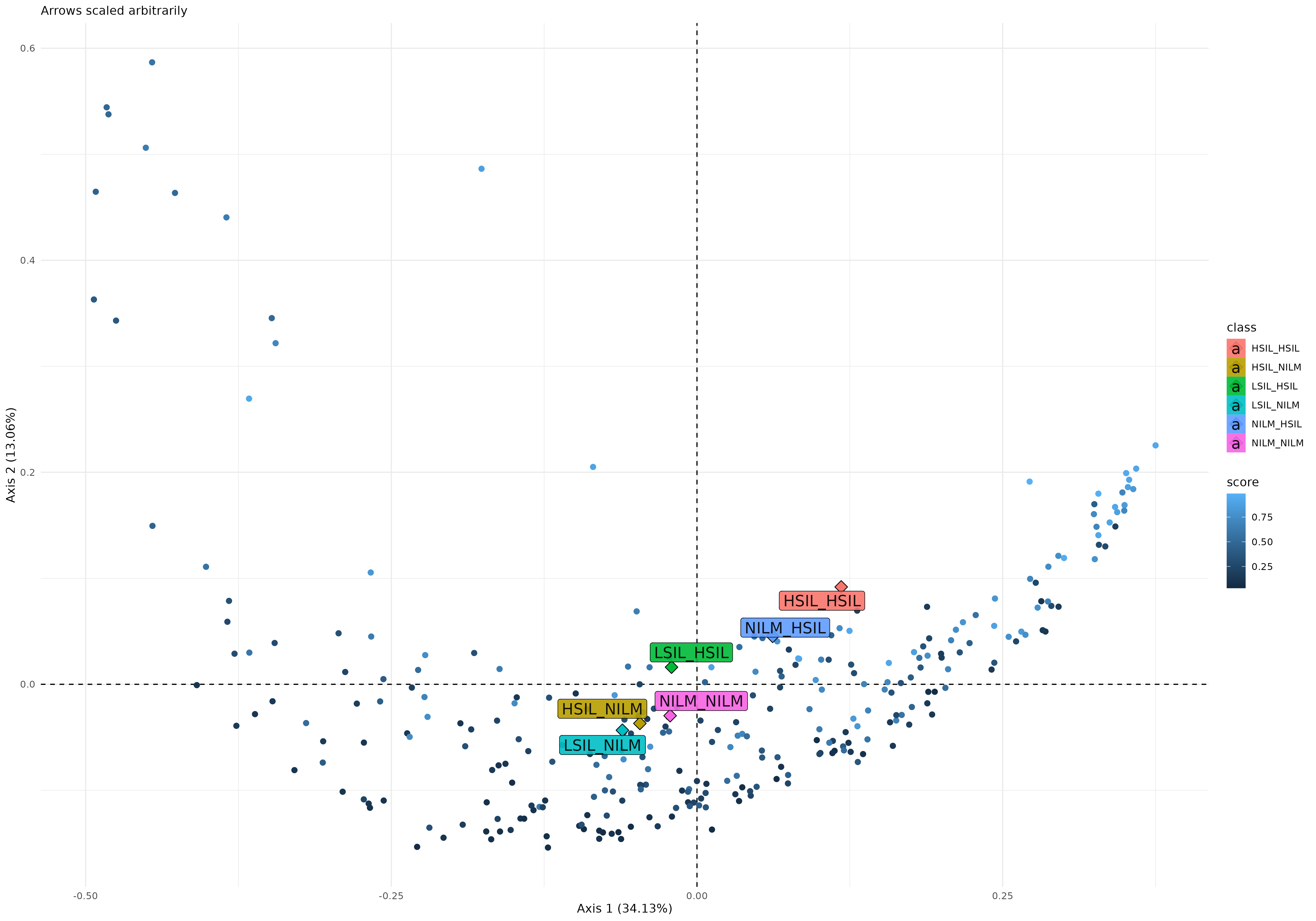

Supplement: Supplementary file 2 — Supplementary Material 2: SuppData 2. [file 10020_2025_1238_MOESM2_ESM.zip › SuppData2/PCOAs/score_PCOA_SH_elasticnet.png]

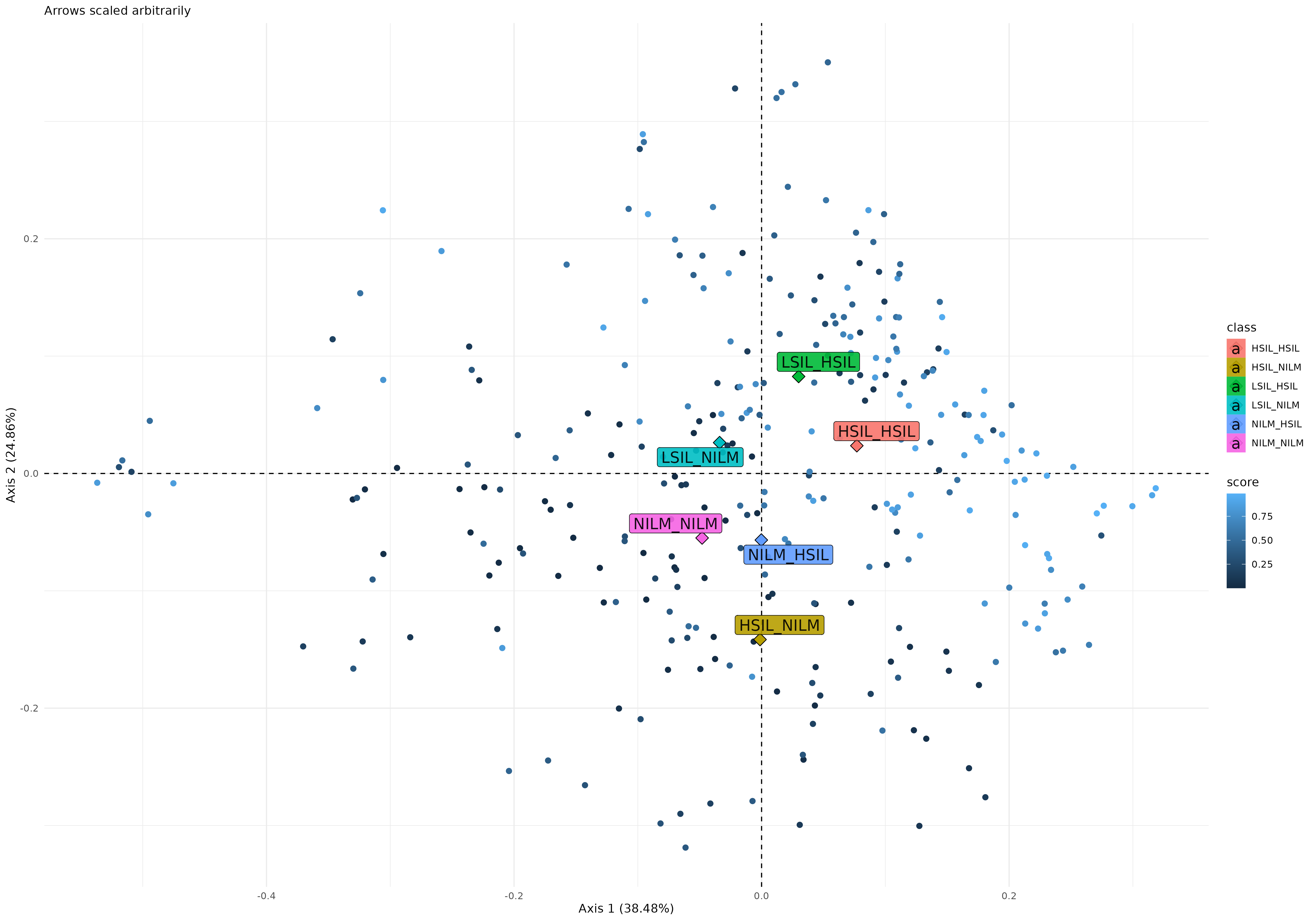

Supplement: Supplementary file 2 — Supplementary Material 2: SuppData 2. [file 10020_2025_1238_MOESM2_ESM.zip › SuppData2/PCOAs/score_PCOA_SuSH_rf.png]

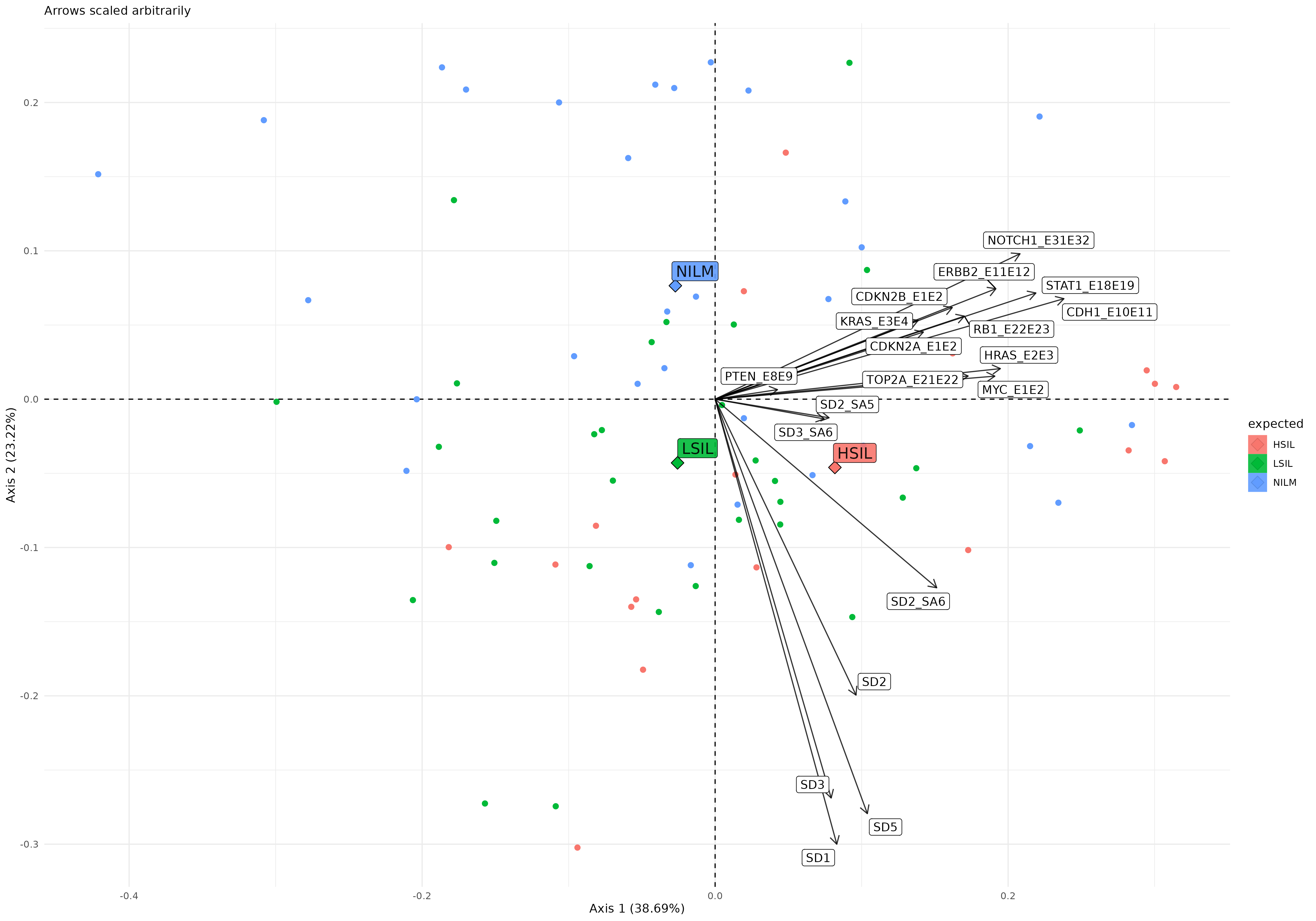

Supplement: Supplementary file 2 — Supplementary Material 2: SuppData 2. [file 10020_2025_1238_MOESM2_ESM.zip › SuppData2/PCOAs/Validation_PCOA_SuSH_rf.png]
