# Supplementary material for: Molecular detection of hrHPV-induced high-grade squamous intraepithelial lesions of the cervix through a targeted RNA next generation sequencing assay
Source: Mol Med. 2025 May 30;31:215. doi: 10.1186/s10020-025-01238-x (PMC12125924; doi:10.1186/s10020-025-01238-x)
Supplement: Supplementary file 2 — Supplementary Material 2: SuppData 2. [file 10020_2025_1238_MOESM2_ESM.zip › SuppData2/Boxplots_predictors.pdf]

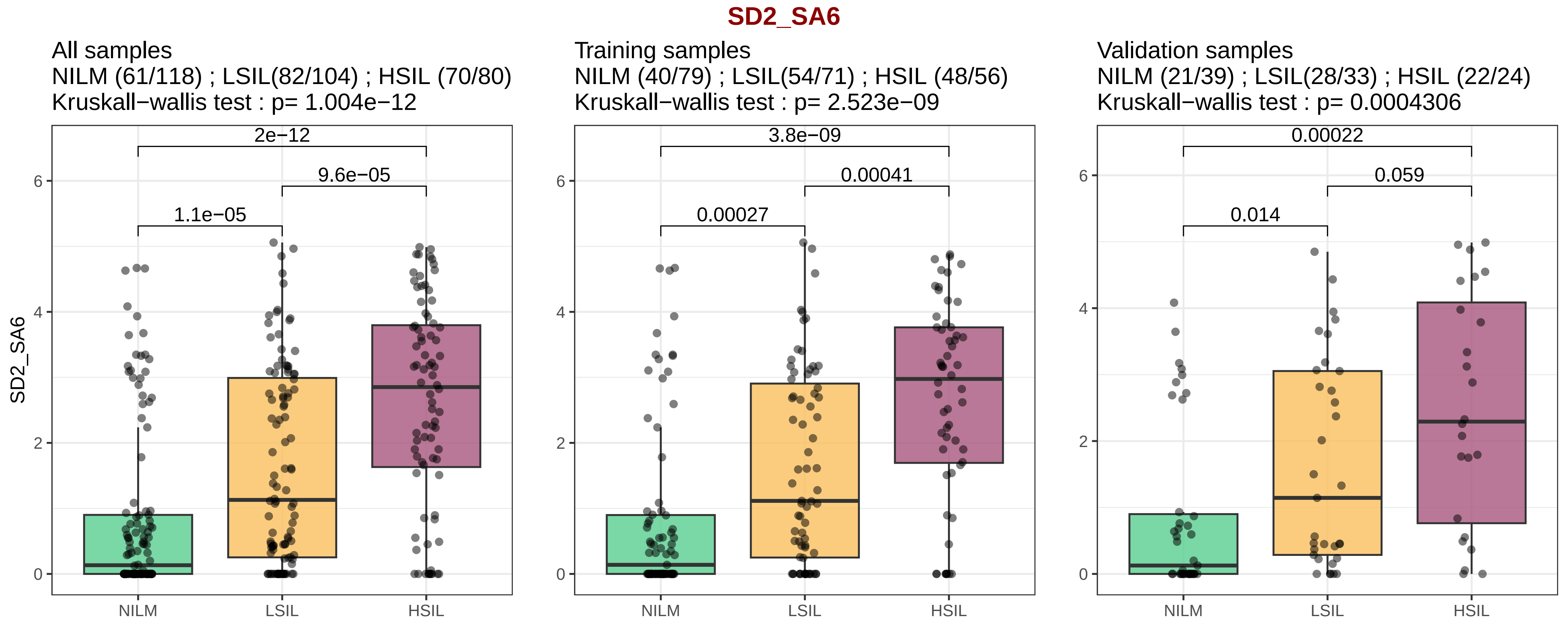

SD2_SA6
All samples
NILM (61/118) ; LSIL(82/104) ; HSIL (70/80)
Kruskall−wallis test : p= 1.004e−12
2e−12
9.6e−05
1.1e−05
Training samples
NILM (40/79) ; LSIL(54/71) ; HSIL (48/56)
Kruskall−wallis test : p= 2.523e−09
3.8e−09
0.00041
0.00027
Validation samples
NILM (21/39) ; LSIL(28/33) ; HSIL (22/24)
Kruskall−wallis test : p= 0.0004306
0.00022
0.059
0.014
SD2_SA6
0
2
4
6
NILM
LSIL
HSIL

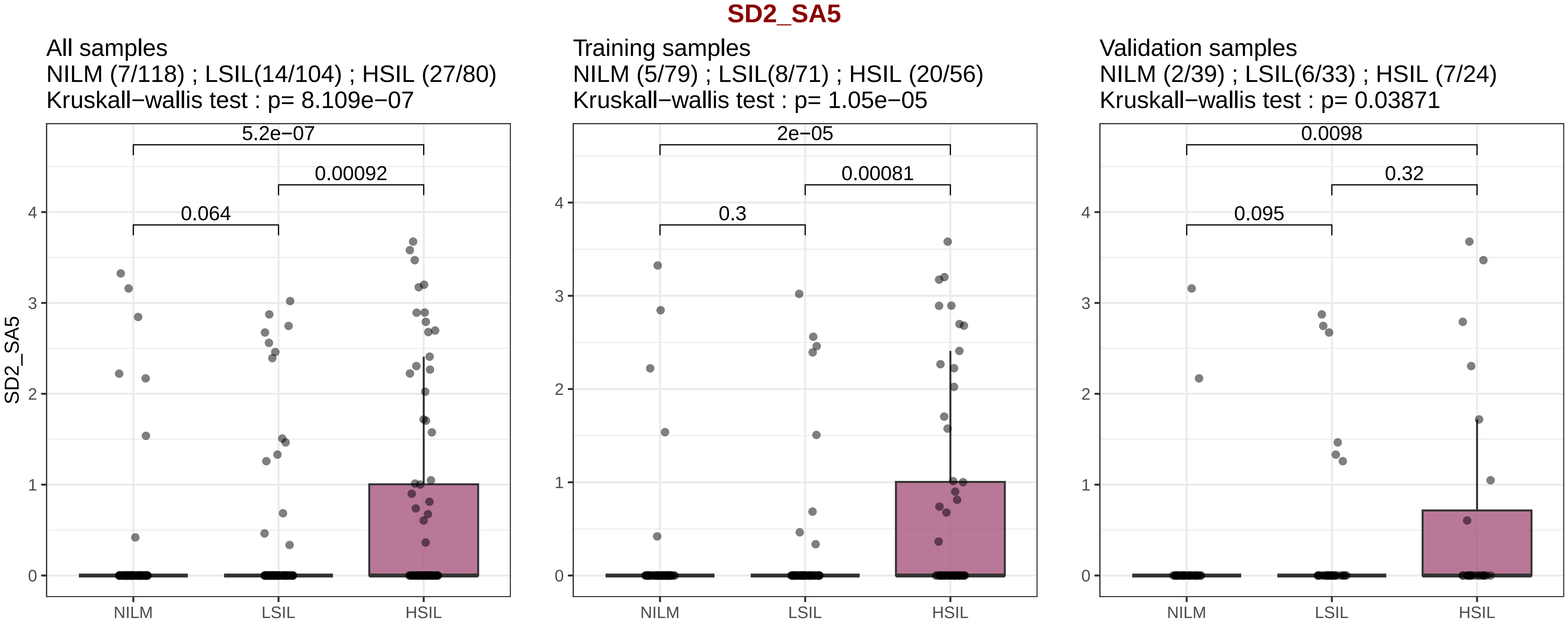
SD2_SA5
All samples
NILM (7/118) ; LSIL(14/104) ; HSIL (27/80)
Kruskall−wallis test : p= 8.109e−07
5.2e−07
0.00092
0.064
Training samples
NILM (5/79) ; LSIL(8/71) ; HSIL (20/56)
Kruskall−wallis test : p= 1.05e−05
2e−05
0.00081
0.3
Validation samples
NILM (2/39) ; LSIL(6/33) ; HSIL (7/24)
Kruskall−wallis test : p= 0.03871
0.0098
0.32
0.095
SD2_SA5
0
1
2
3
4
NILM
LSIL
HSIL

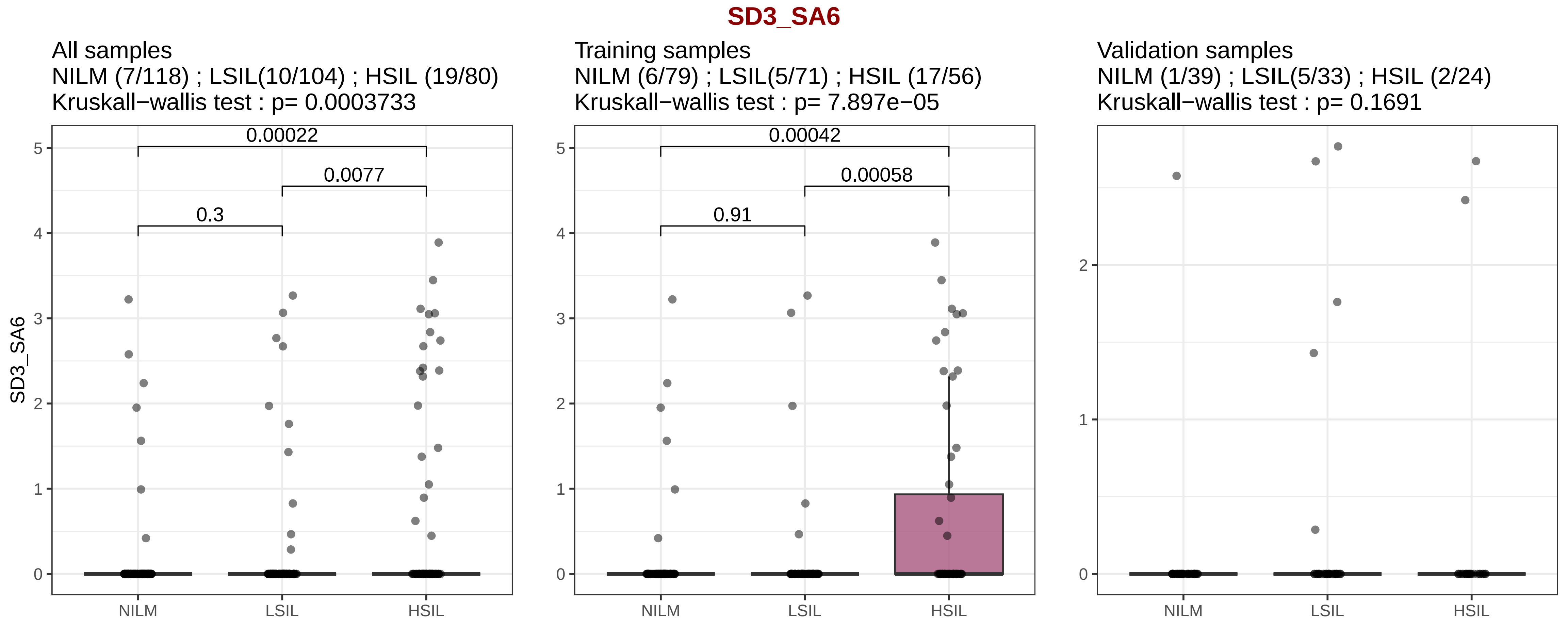

SD3_SA6
All samples
NILM (7/118) ; LSIL(10/104) ; HSIL (19/80)
Kruskall−wallis test : p= 0.0003733
0.00022
0.0077
0.3
Training samples
NILM (6/79) ; LSIL(5/71) ; HSIL (17/56)
Kruskall−wallis test : p= 7.897e−05
0.00042
0.00058
0.91
Validation samples
NILM (1/39) ; LSIL(5/33) ; HSIL (2/24)
Kruskall−wallis test : p= 0.1691
SD3_SA6
NILM
LSIL
HSIL

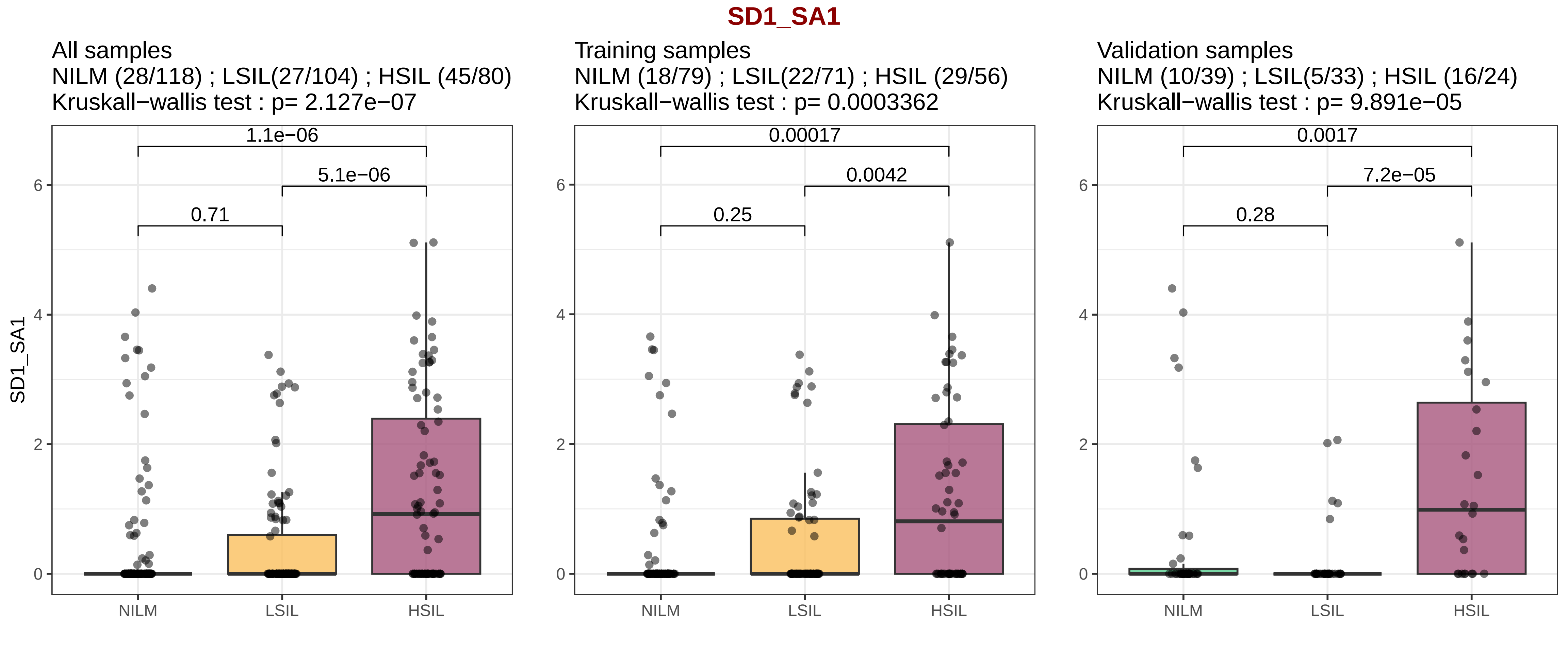
SD1_SA1
All samples
NILM (28/118) ; LSIL(27/104) ; HSIL (45/80)
Kruskall−wallis test : p= 2.127e−07
1.1e−06
5.1e−06
0.71
Training samples
NILM (18/79) ; LSIL(22/71) ; HSIL (29/56)
Kruskall−wallis test : p= 0.0003362
0.00017
0.0042
0.25
Validation samples
NILM (10/39) ; LSIL(5/33) ; HSIL (16/24)
Kruskall−wallis test : p= 9.891e−05
0.0017
7.2e−05
0.28
SD1_SA1
0
2
4
6
NILM
LSIL
HSIL

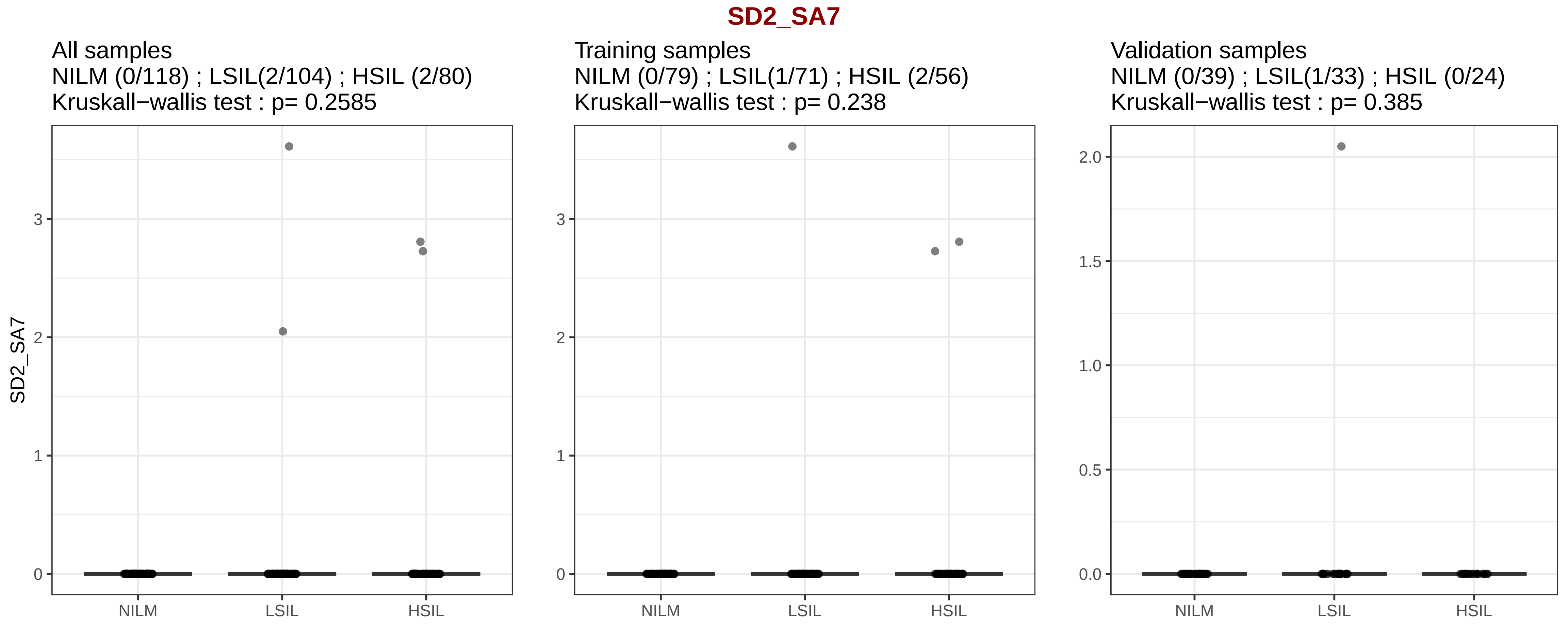
SD2_SA7
All samples
NILM (0/118) ; LSIL(2/104) ; HSIL (2/80)
Kruskall−wallis test : p= 0.2585
Training samples
NILM (0/79) ; LSIL(1/71) ; HSIL (2/56)
Kruskall−wallis test : p= 0.238
Validation samples
NILM (0/39) ; LSIL(1/33) ; HSIL (0/24)
Kruskall−wallis test : p= 0.385
SD2_SA7
0
1
2
3
0.0
0.5
1.0
1.5
2.0
NILM
LSIL
HSIL

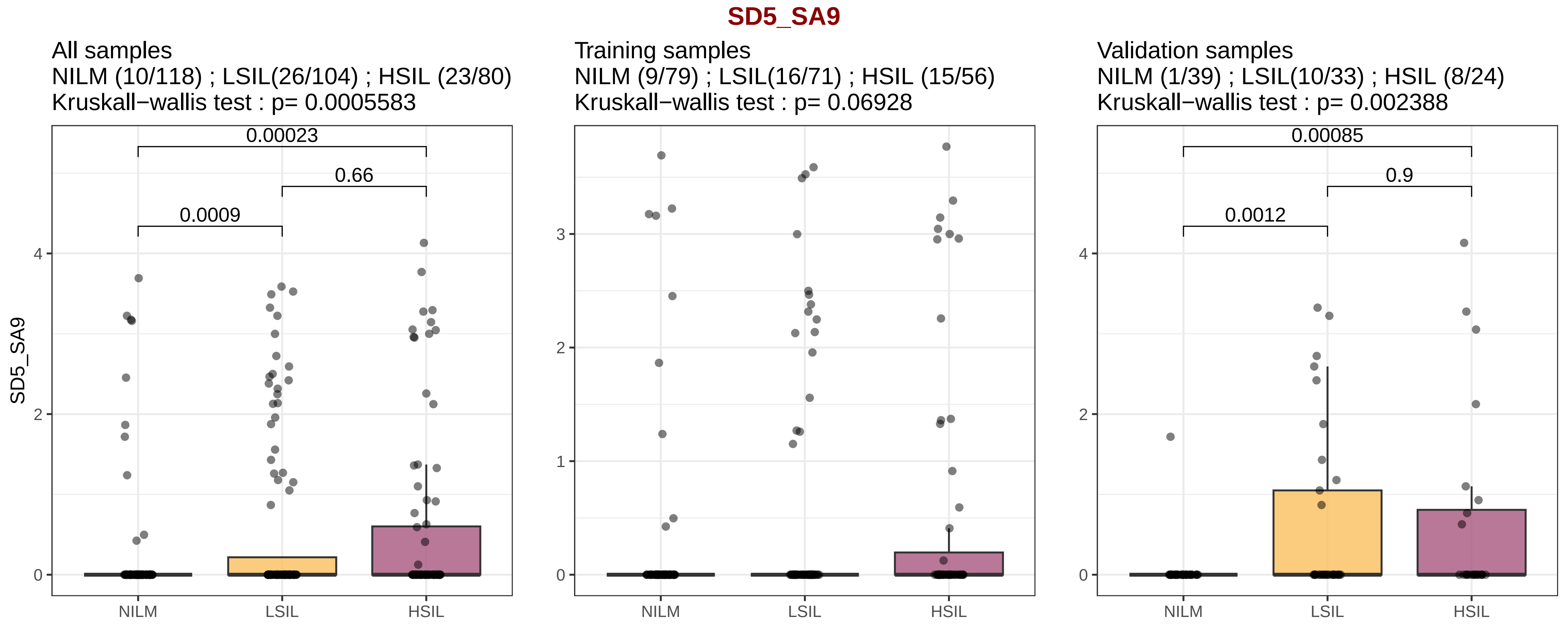
SD5_SA9
All samples
NILM (10/118) ; LSIL(26/104) ; HSIL (23/80)
Kruskall−wallis test : p= 0.0005583
Training samples
NILM (9/79) ; LSIL(16/71) ; HSIL (15/56)
Kruskall−wallis test : p= 0.06928
Validation samples
NILM (1/39) ; LSIL(10/33) ; HSIL (8/24)
Kruskall−wallis test : p= 0.002388
SD5_SA9
0.00023
0.66
0.0009
0.00085
0.9
0.0012
0
1
2
3
4
NILM
LSIL
HSIL

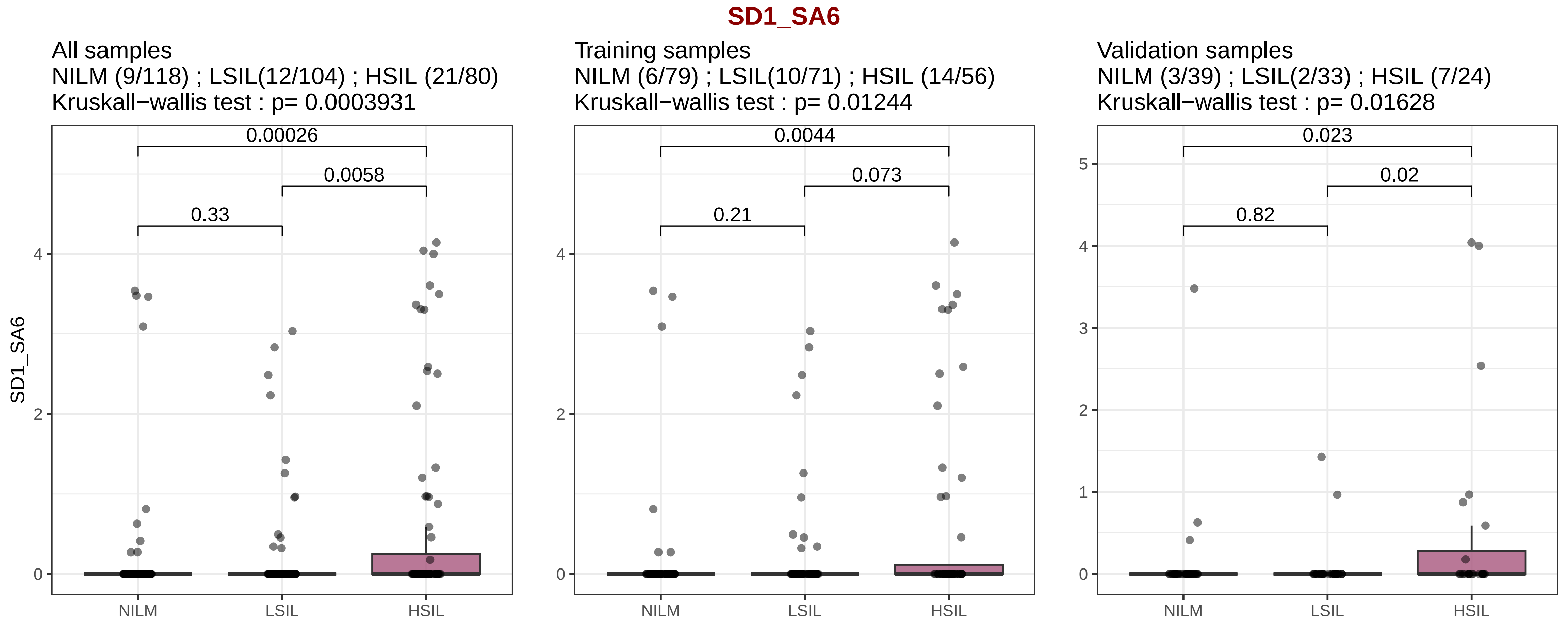

SD1_SA6
All samples
NILM (9/118) ; LSIL(12/104) ; HSIL (21/80)
Kruskall−wallis test : p= 0.0003931
0.00026
0.0058
0.33
Training samples
NILM (6/79) ; LSIL(10/71) ; HSIL (14/56)
Kruskall−wallis test : p= 0.01244
0.0044
0.073
0.21
Validation samples
NILM (3/39) ; LSIL(2/33) ; HSIL (7/24)
Kruskall−wallis test : p= 0.01628
0.023
0.02
0.82
SD1_SA6
NILM
LSIL
HSIL

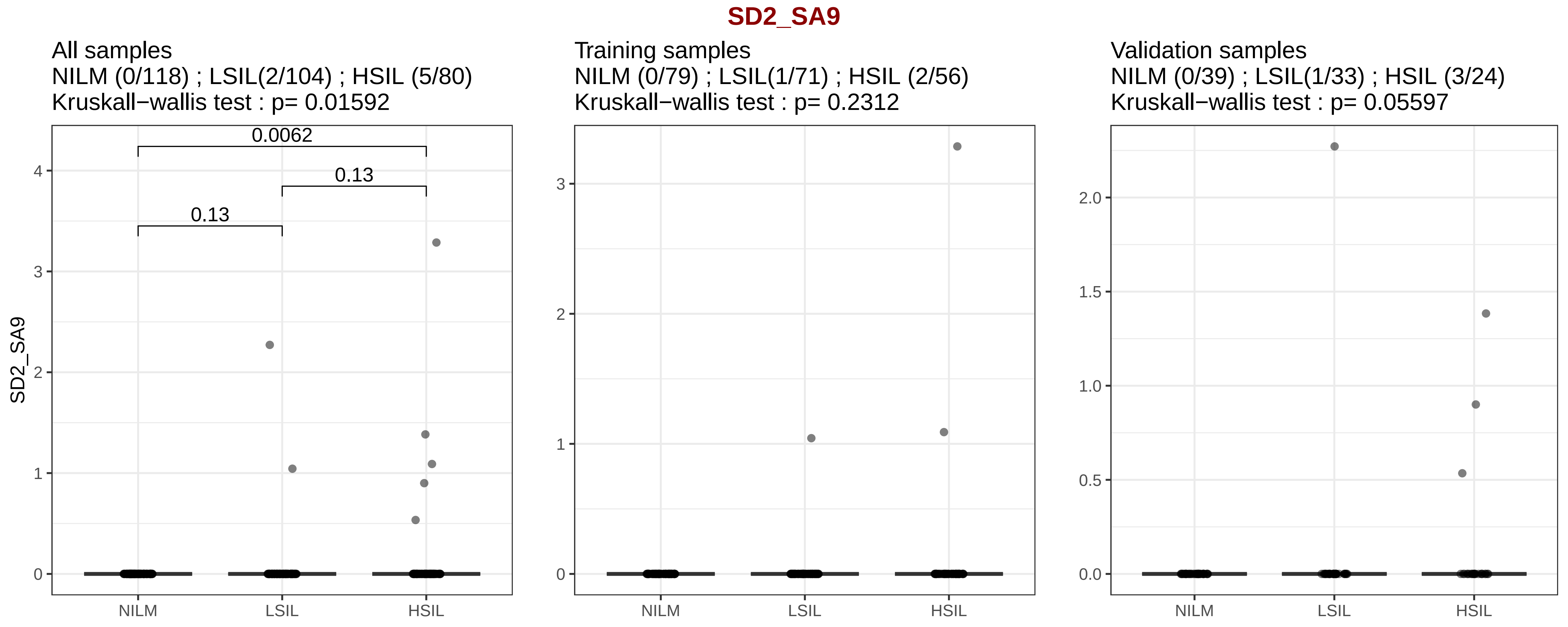

SD2_SA9
All samples
NILM (0/118) ; LSIL(2/104) ; HSIL (5/80)
Kruskall−wallis test : p= 0.01592
0.0062
0.13
0.13
SD2_SA9
0
1
2
3
4
NILM
LSIL
HSIL
Training samples
NILM (0/79) ; LSIL(1/71) ; HSIL (2/56)
Kruskall−wallis test : p= 0.2312
0
1
2
3
NILM
LSIL
HSIL
Validation samples
NILM (0/39) ; LSIL(1/33) ; HSIL (3/24)
Kruskall−wallis test : p= 0.05597
0.0
0.5
1.0
1.5
2.0
NILM
LSIL
HSIL

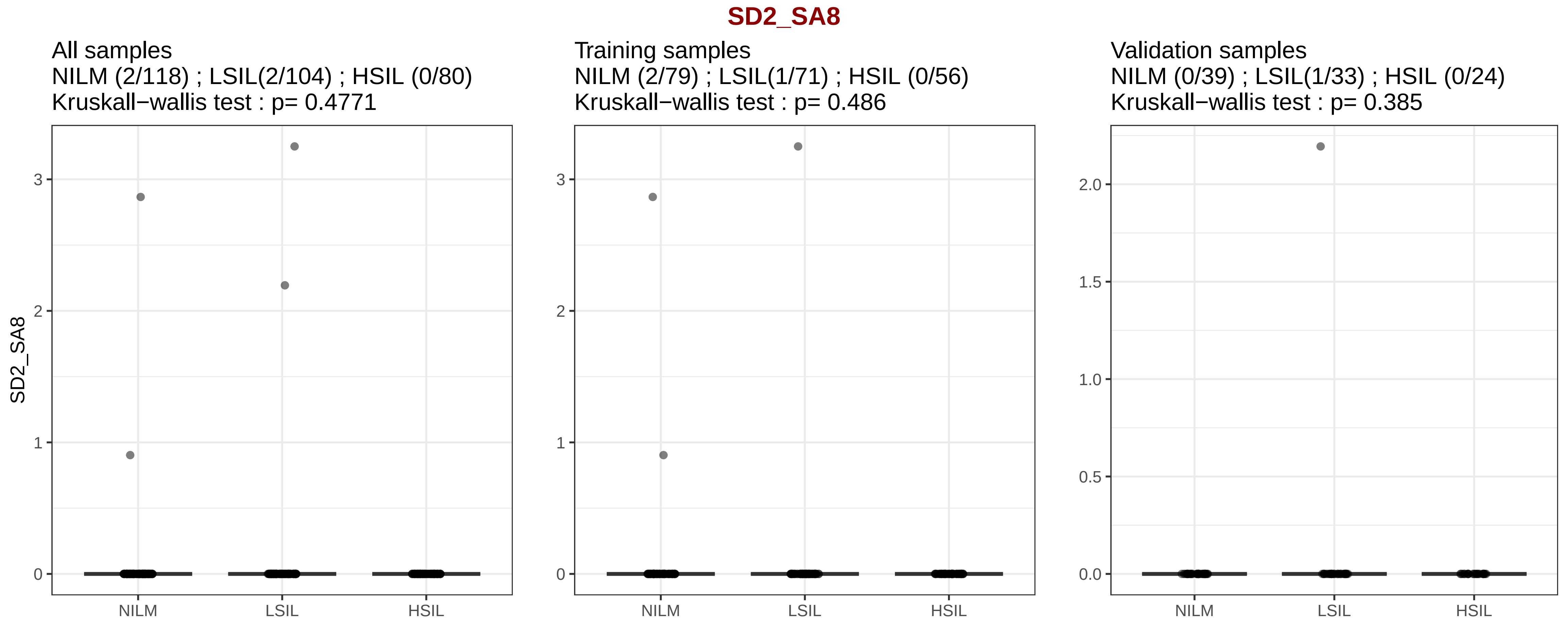

SD2_SA8
All samples
NILM (2/118) ; LSIL(2/104) ; HSIL (0/80)
Kruskall−wallis test : p= 0.4771
Training samples
NILM (2/79) ; LSIL(1/71) ; HSIL (0/56)
Kruskall−wallis test : p= 0.486
Validation samples
NILM (0/39) ; LSIL(1/33) ; HSIL (0/24)
Kruskall−wallis test : p= 0.385
SD2_SA8
3
2
1
0
2.0
1.5
1.0
0.5
0.0
NILM
LSIL
HSIL

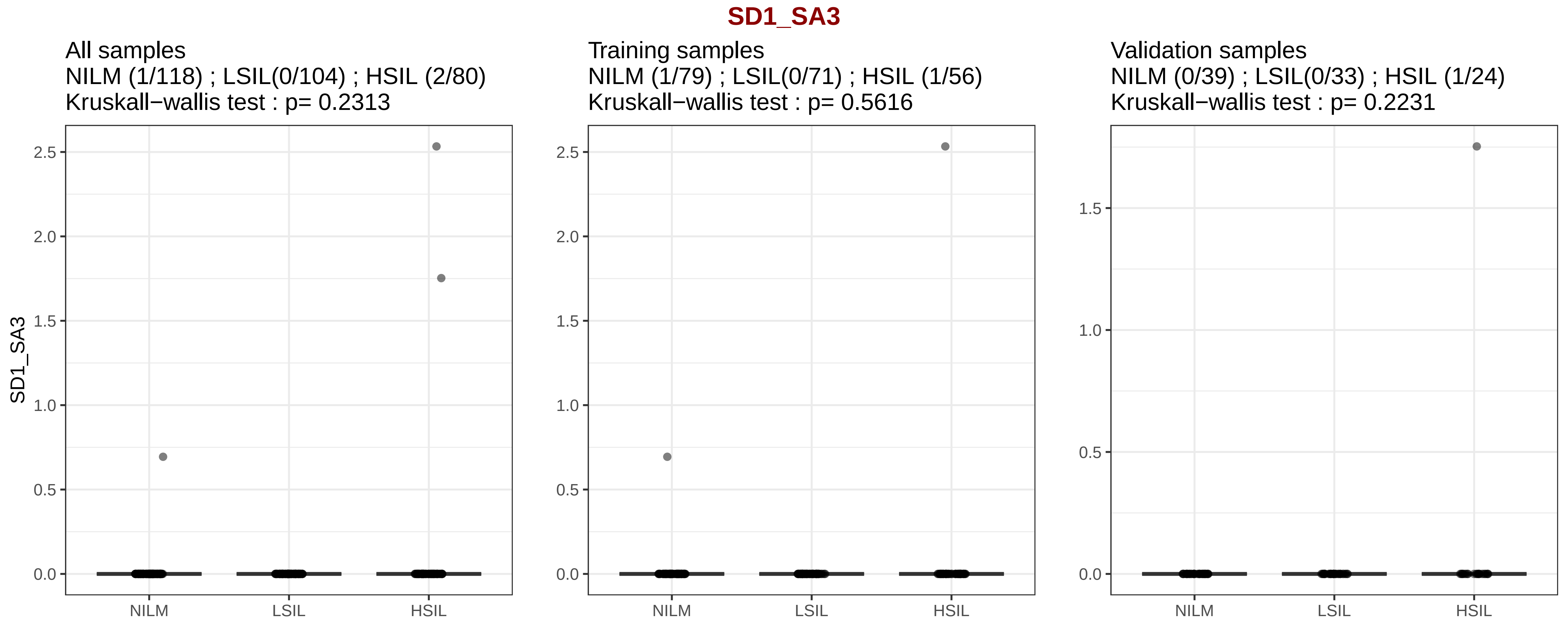
SD1_SA3
All samples
NILM (1/118) ; LSIL(0/104) ; HSIL (2/80)
Kruskall−wallis test : p= 0.2313
Training samples
NILM (1/79) ; LSIL(0/71) ; HSIL (1/56)
Kruskall−wallis test : p= 0.5616
Validation samples
NILM (0/39) ; LSIL(0/33) ; HSIL (1/24)
Kruskall−wallis test : p= 0.2231
SD1_SA3
0.0
0.5
1.0
1.5
2.0
2.5
NILM
LSIL
HSIL

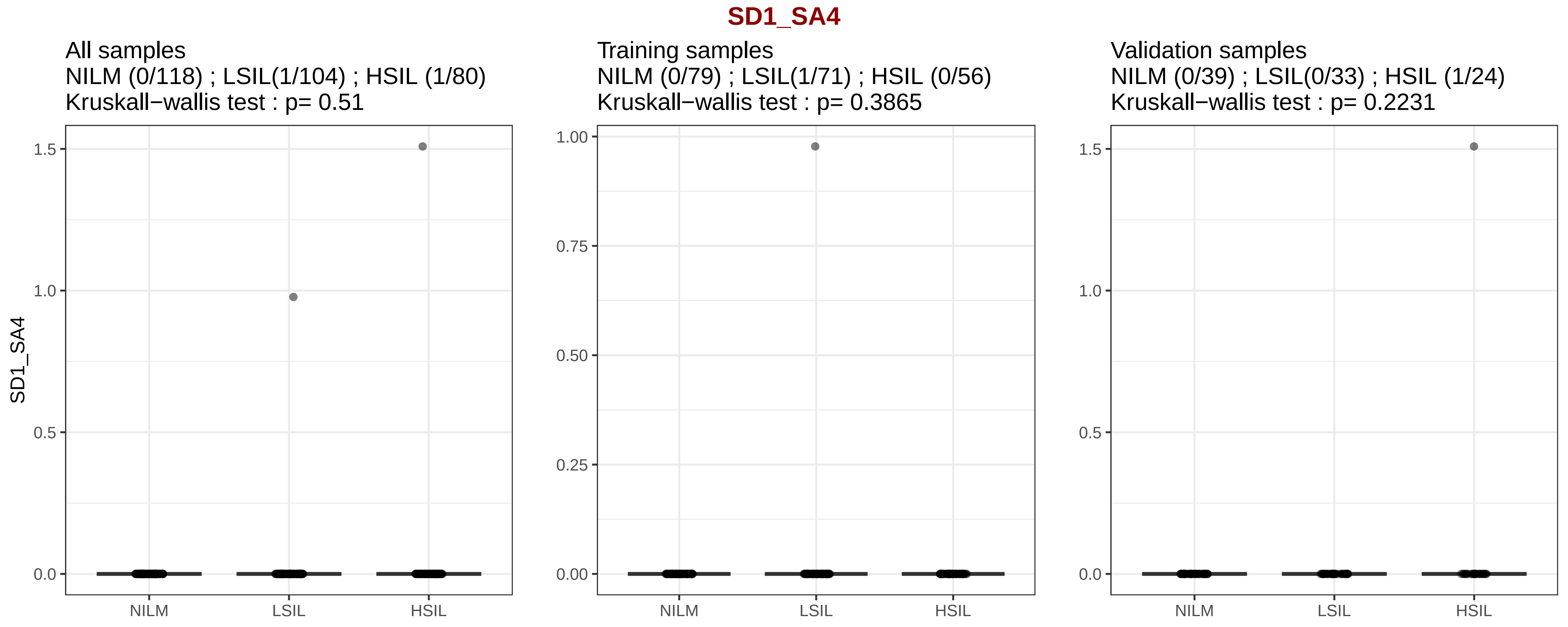

SD1_SA4
All samples
NILM (0/118) ; LSIL(1/104) ; HSIL (1/80)
Kruskall−wallis test : p= 0.51
Training samples
NILM (0/79) ; LSIL(1/71) ; HSIL (0/56)
Kruskall−wallis test : p= 0.3865
Validation samples
NILM (0/39) ; LSIL(0/33) ; HSIL (1/24)
Kruskall−wallis test : p= 0.2231
SD1_SA4
1.5
1.0
0.5
0.0
1.00
0.75
0.50
0.25
0.00
NILM
LSIL
HSIL

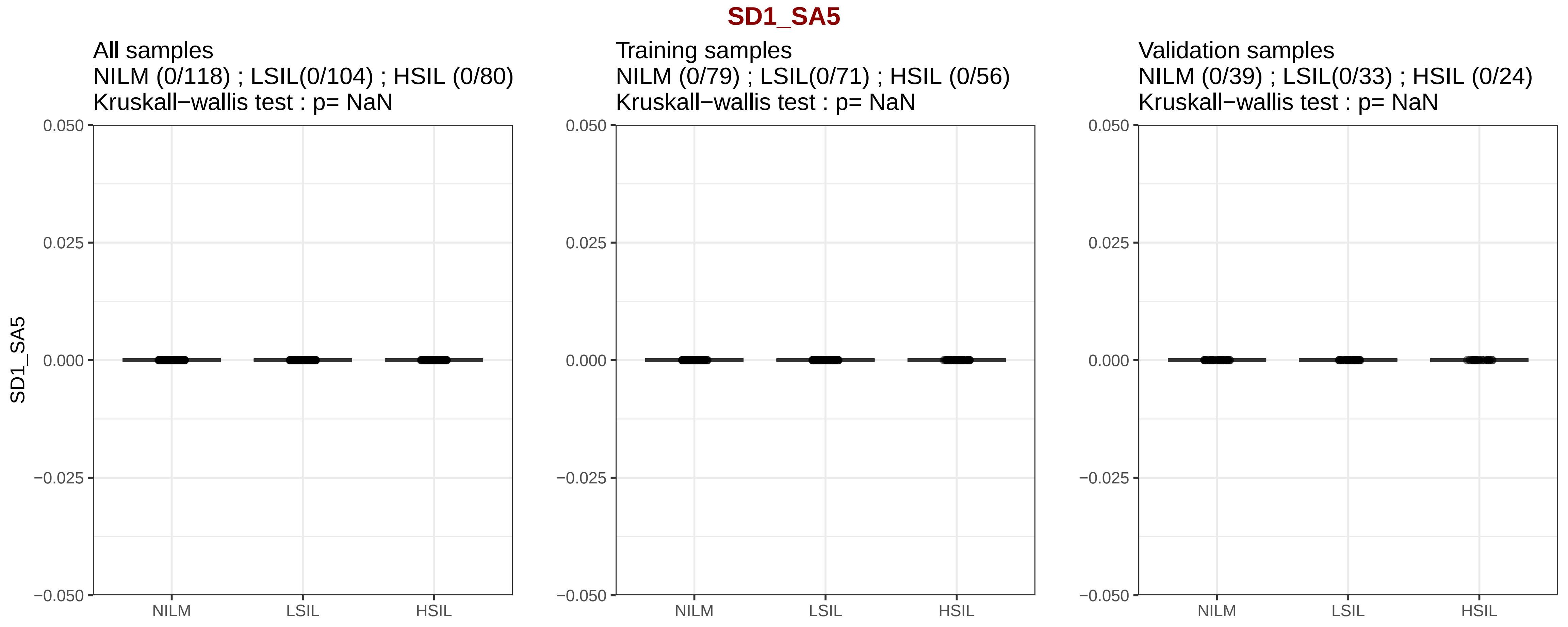
SD1_SA5
All samples
NILM (0/118) ; LSIL(0/104) ; HSIL (0/80)
Kruskall−wallis test : p= NaN
Training samples
NILM (0/79) ; LSIL(0/71) ; HSIL (0/56)
Kruskall−wallis test : p= NaN
Validation samples
NILM (0/39) ; LSIL(0/33) ; HSIL (0/24)
Kruskall−wallis test : p= NaN
SD1_SA5
0.050
0.025
0.000
−0.025
−0.050
NILM
LSIL
HSIL

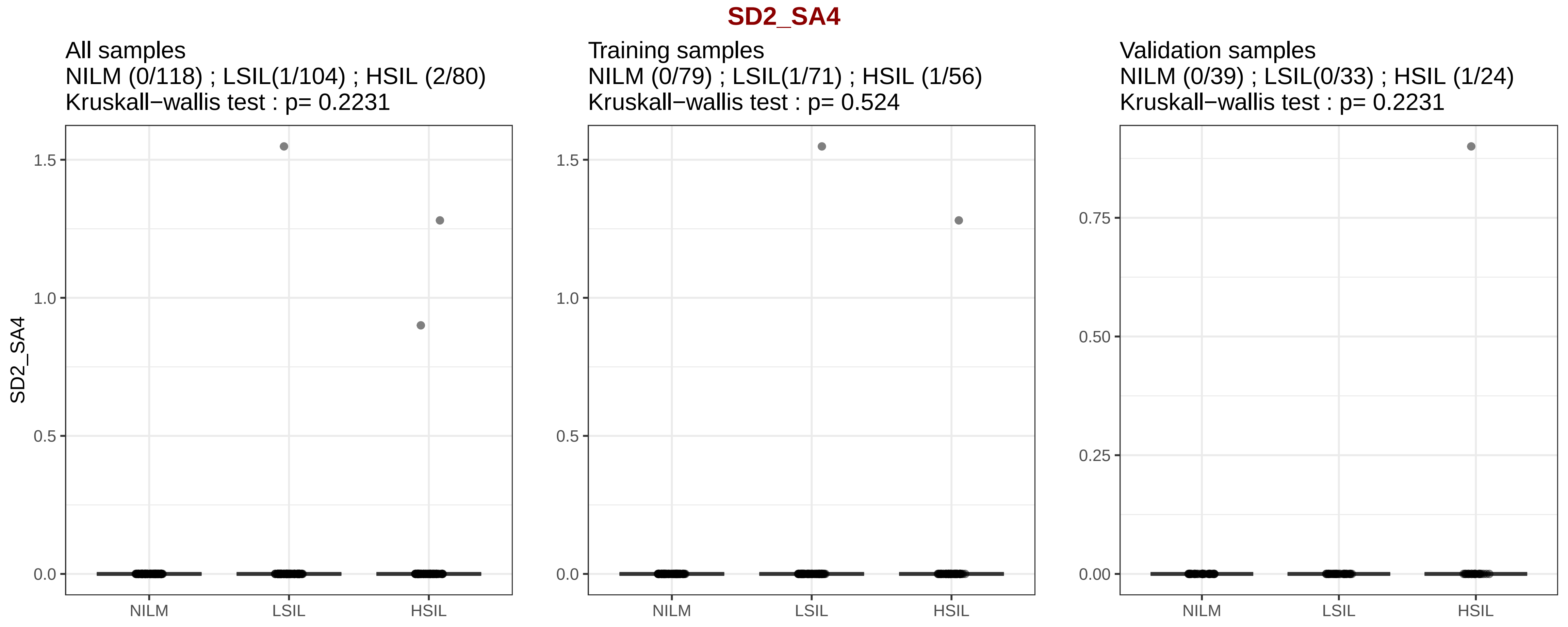
SD2_SA4
All samples
NILM (0/118) ; LSIL(1/104) ; HSIL (2/80)
Kruskall−wallis test : p= 0.2231
Training samples
NILM (0/79) ; LSIL(1/71) ; HSIL (1/56)
Kruskall−wallis test : p= 0.524
Validation samples
NILM (0/39) ; LSIL(0/33) ; HSIL (1/24)
Kruskall−wallis test : p= 0.2231
SD2_SA4
1.5
1.0
0.5
0.0
0.75
0.50
0.25
0.00
NILM
LSIL
HSIL

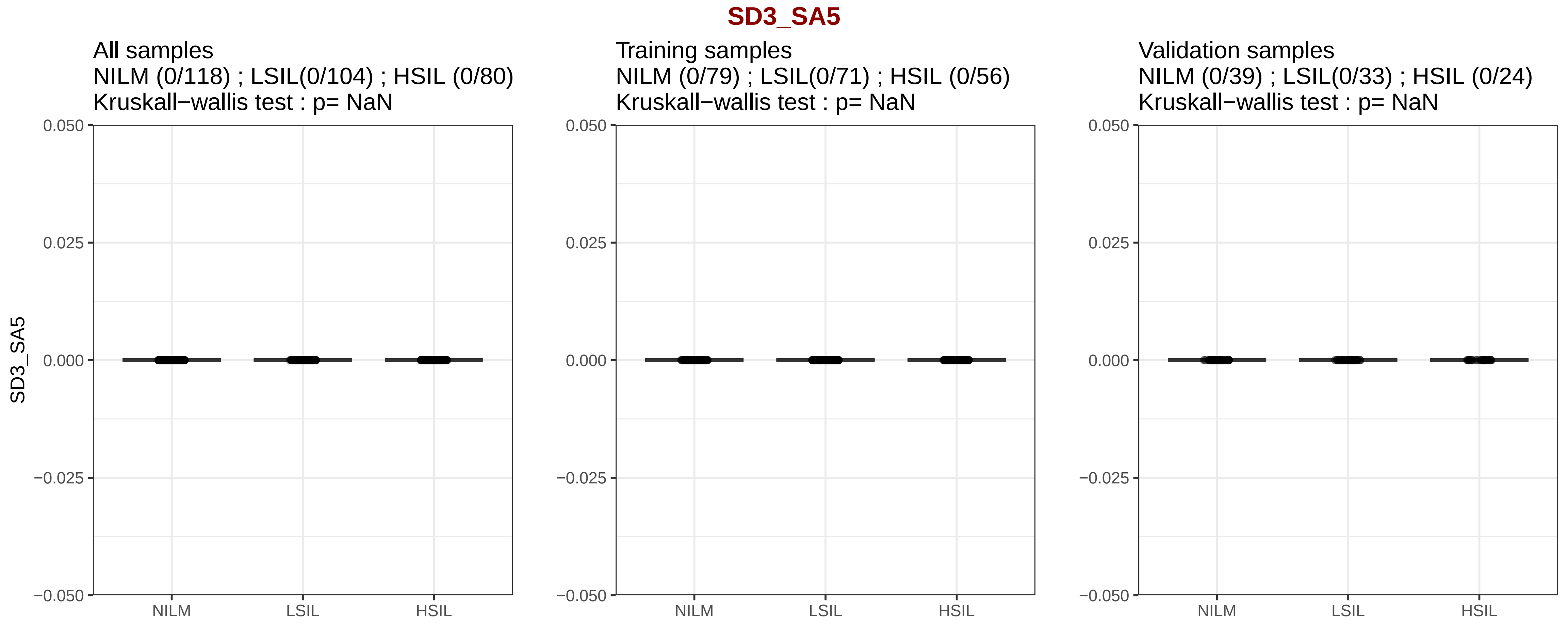
SD3_SA5
All samples
NILM (0/118) ; LSIL(0/104) ; HSIL (0/80)
Kruskall−wallis test : p= NaN
Training samples
NILM (0/79) ; LSIL(0/71) ; HSIL (0/56)
Kruskall−wallis test : p= NaN
Validation samples
NILM (0/39) ; LSIL(0/33) ; HSIL (0/24)
Kruskall−wallis test : p= NaN
SD3_SA5
0.050
0.025
0.000
−0.025
−0.050
NILM
LSIL
HSIL

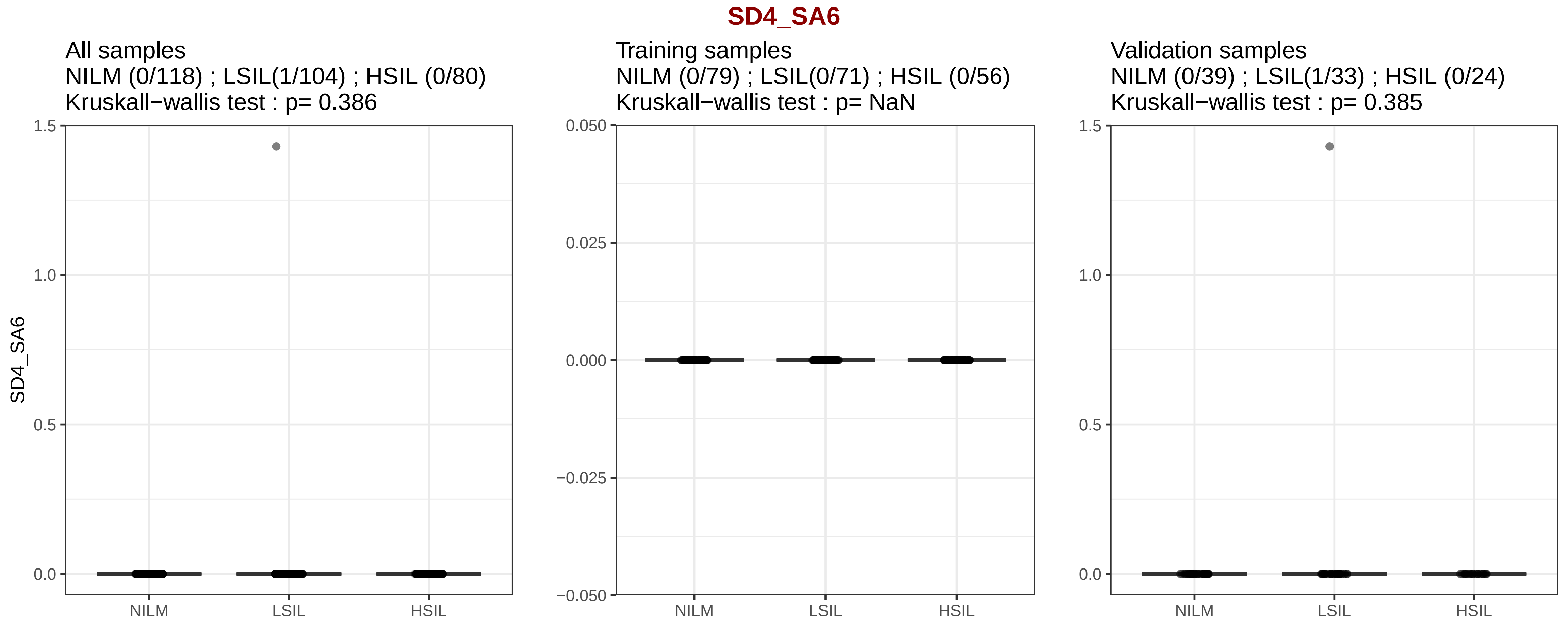
SD4_SA6
All samples
NILM (0/118) ; LSIL(1/104) ; HSIL (0/80)
Kruskall−wallis test : p= 0.386
Training samples
NILM (0/79) ; LSIL(0/71) ; HSIL (0/56)
Kruskall−wallis test : p= NaN
Validation samples
NILM (0/39) ; LSIL(1/33) ; HSIL (0/24)
Kruskall−wallis test : p= 0.385
SD4_SA6
1.5
1.0
0.5
0.0
0.050
0.025
0.000
−0.025
−0.050
NILM
LSIL
HSIL

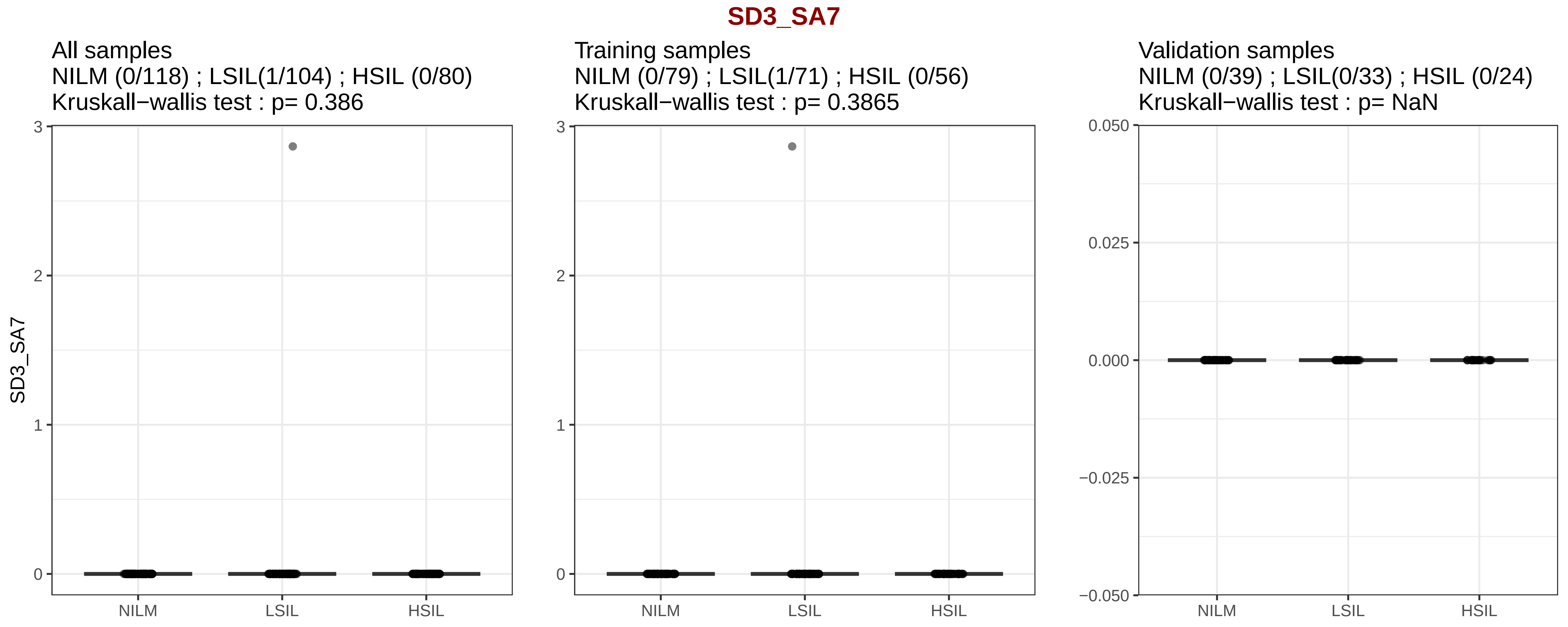
SD3_SA7
All samples
NILM (0/118) ; LSIL(1/104) ; HSIL (0/80)
Kruskall−wallis test : p= 0.386
Training samples
NILM (0/79) ; LSIL(1/71) ; HSIL (0/56)
Kruskall−wallis test : p= 0.3865
Validation samples
NILM (0/39) ; LSIL(0/33) ; HSIL (0/24)
Kruskall−wallis test : p= NaN
SD3_SA7
0
1
2
3
0.050
0.025
0.000
−0.025
−0.050
NILM
LSIL
HSIL

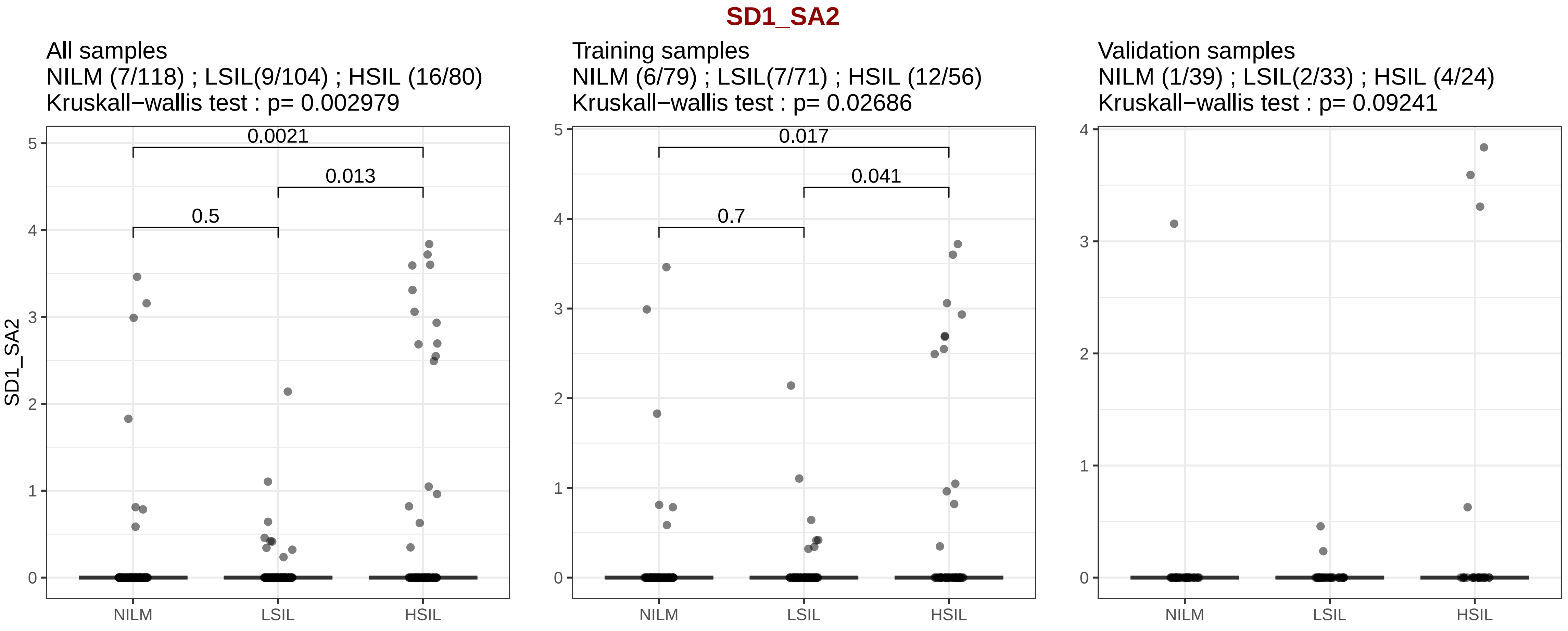

SD1_SA2
All samples
NILM (7/118) ; LSIL(9/104) ; HSIL (16/80)
Kruskall−wallis test : p= 0.002979
0.0021
0.013
0.5
SD1_SA2
0
1
2
3
4
5
NILM
LSIL
HSIL
Training samples
NILM (6/79) ; LSIL(7/71) ; HSIL (12/56)
Kruskall−wallis test : p= 0.02686
0.017
0.041
0.7
0
1
2
3
4
5
NILM
LSIL
HSIL
Validation samples
NILM (1/39) ; LSIL(2/33) ; HSIL (4/24)
Kruskall−wallis test : p= 0.09241
0
1
2
3
4
NILM
LSIL
HSIL

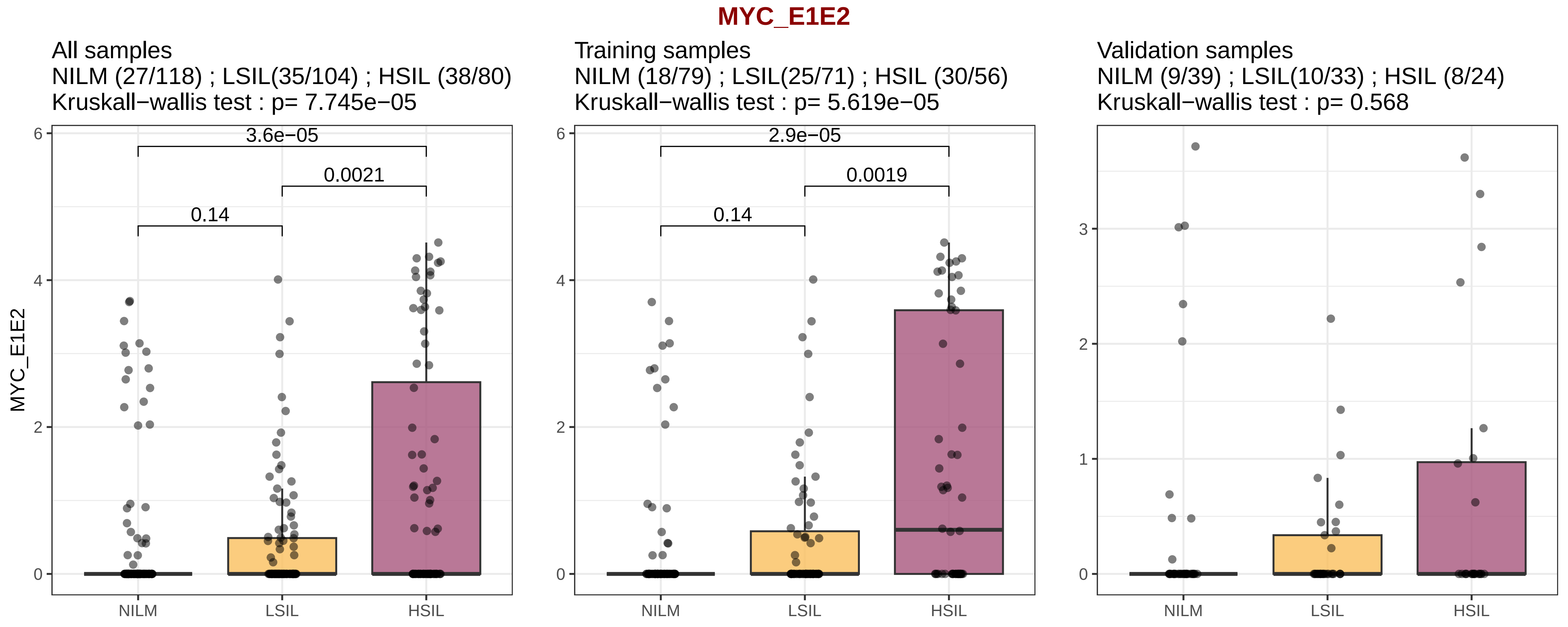
MYC_E1E2
All samples
NILM (27/118) ; LSIL(35/104) ; HSIL (38/80)
Kruskall−wallis test : p= 7.745e−05
Training samples
NILM (18/79) ; LSIL(25/71) ; HSIL (30/56)
Kruskall−wallis test : p= 5.619e−05
Validation samples
NILM (9/39) ; LSIL(10/33) ; HSIL (8/24)
Kruskall−wallis test : p= 0.568
3.6e−05
0.0021
0.14
2.9e−05
0.0019
0.14
MYC_E1E2
NILM
LSIL
HSIL

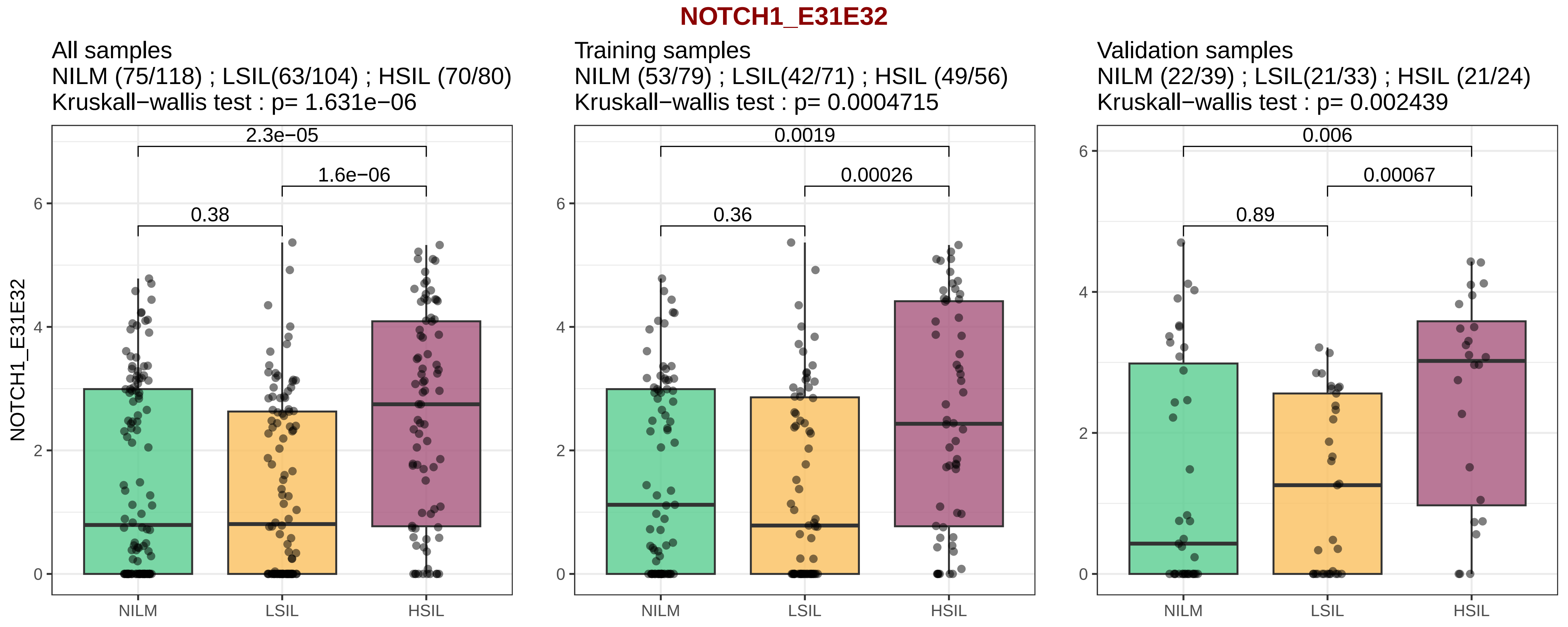

NOTCH1_E31E32
All samples
NILM (75/118) ; LSIL(63/104) ; HSIL (70/80)
Kruskall−wallis test : p= 1.631e−06
2.3e−05
1.6e−06
0.38
Training samples
NILM (53/79) ; LSIL(42/71) ; HSIL (49/56)
Kruskall−wallis test : p= 0.0004715
0.0019
0.00026
0.36
Validation samples
NILM (22/39) ; LSIL(21/33) ; HSIL (21/24)
Kruskall−wallis test : p= 0.002439
0.006
0.00067
0.89
NOTCH1_E31E32
0
2
4
6
NILM
LSIL
HSIL

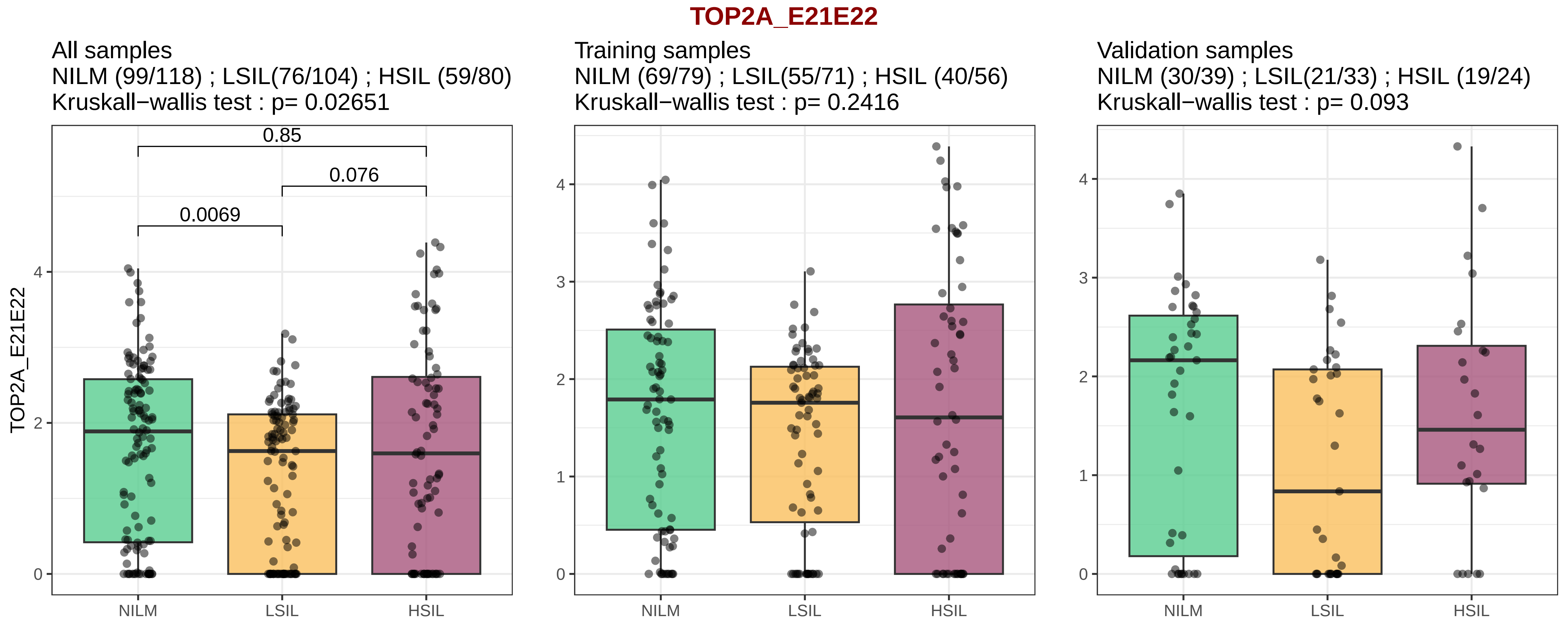
TOP2A_E21E22
All samples
NILM (99/118) ; LSIL(76/104) ; HSIL (59/80)
Kruskall−wallis test : p= 0.02651
0.85
0.076
0.0069
TOP2A_E21E22
0
2
4
NILM
LSIL
HSIL
Training samples
NILM (69/79) ; LSIL(55/71) ; HSIL (40/56)
Kruskall−wallis test : p= 0.2416
0
1
2
3
4
NILM
LSIL
HSIL
Validation samples
NILM (30/39) ; LSIL(21/33) ; HSIL (19/24)
Kruskall−wallis test : p= 0.093
0
1
2
3
4
NILM
LSIL
HSIL

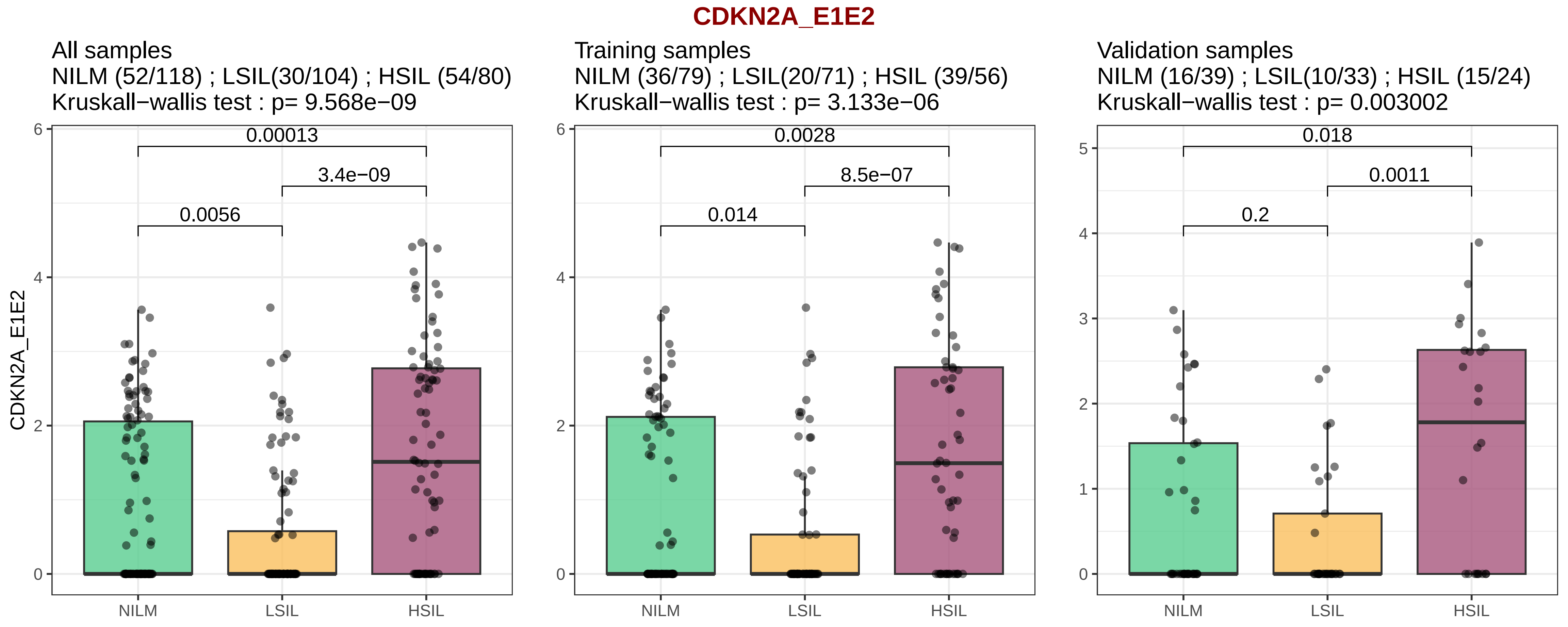

CDKN2A_E1E2
All samples
NILM (52/118) ; LSIL(30/104) ; HSIL (54/80)
Kruskall−wallis test : p= 9.568e−09
0.00013
3.4e−09
0.0056
Training samples
NILM (36/79) ; LSIL(20/71) ; HSIL (39/56)
Kruskall−wallis test : p= 3.133e−06
0.0028
8.5e−07
0.014
Validation samples
NILM (16/39) ; LSIL(10/33) ; HSIL (15/24)
Kruskall−wallis test : p= 0.003002
0.018
0.0011
0.2
CDKN2A_E1E2
NILM
LSIL
HSIL

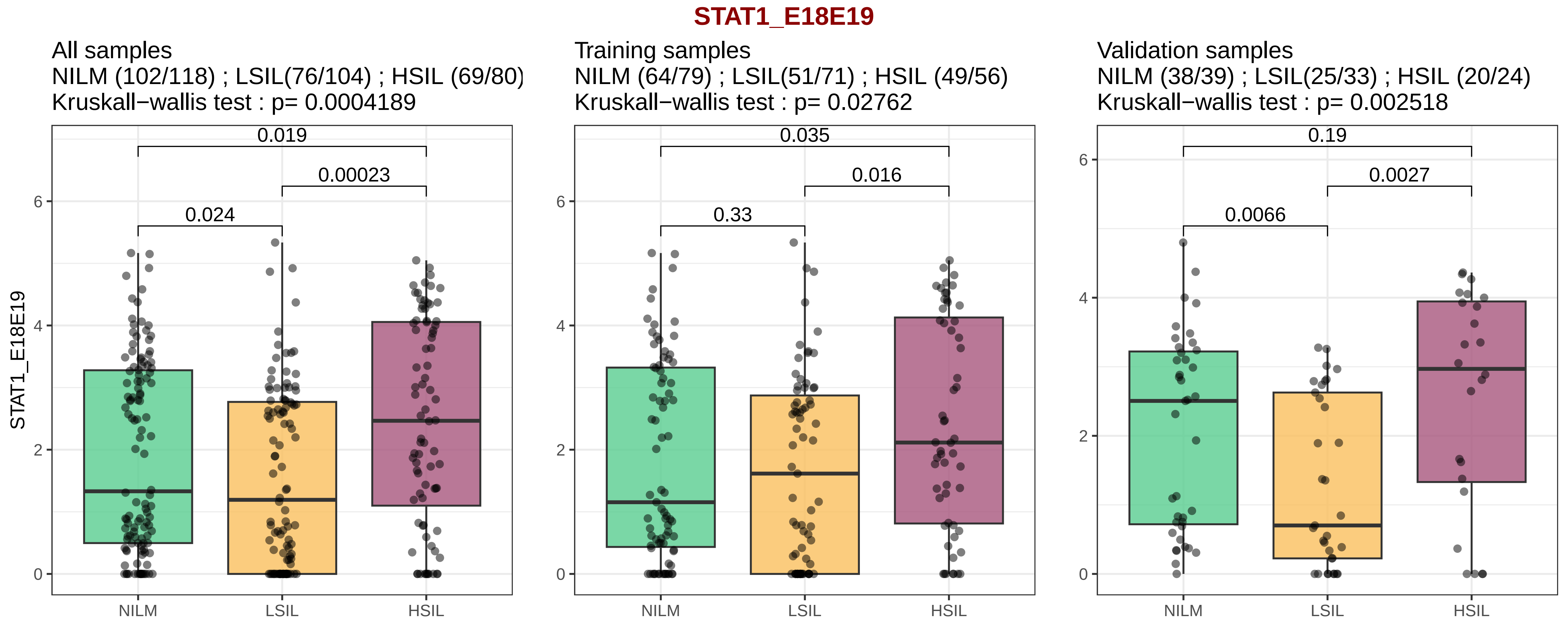
STAT1_E18E19
All samples
NILM (102/118) ; LSIL(76/104) ; HSIL (69/80)
Kruskall−wallis test : p= 0.0004189
0.019
0.00023
0.024
STAT1_E18E19
0
2
4
6
NILM
LSIL
HSIL
Training samples
NILM (64/79) ; LSIL(51/71) ; HSIL (49/56)
Kruskall−wallis test : p= 0.02762
0.035
0.016
0.33
Validation samples
NILM (38/39) ; LSIL(25/33) ; HSIL (20/24)
Kruskall−wallis test : p= 0.002518
0.19
0.0027
0.0066

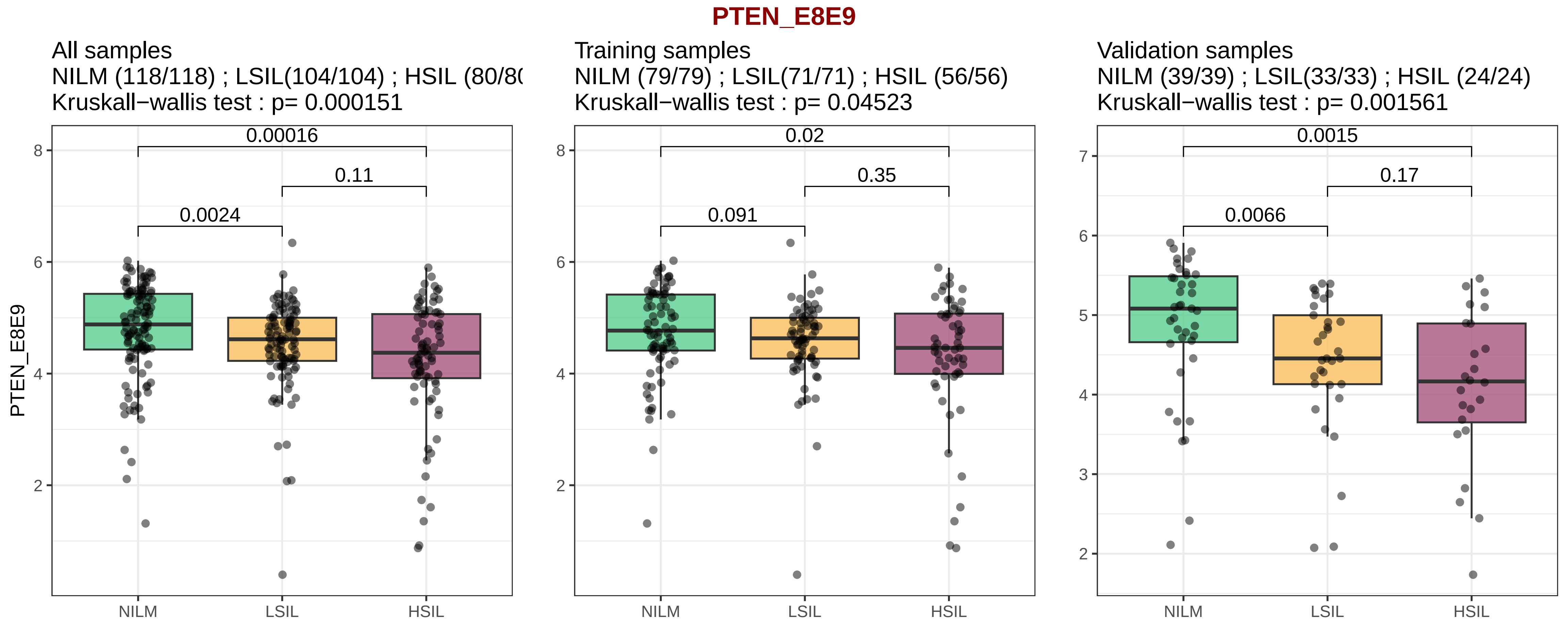
PTEN_E8E9
All samples
NILM (118/118) ; LSIL(104/104) ; HSIL (80/8(
Kruskall−wallis test : p= 0.000151
0.00016
0.11
0.0024
PTEN_E8E9
8
6
4
2
NILM
LSIL
HSIL
Training samples
NILM (79/79) ; LSIL(71/71) ; HSIL (56/56)
Kruskall−wallis test : p= 0.04523
0.02
0.35
0.091
Validation samples
NILM (39/39) ; LSIL(33/33) ; HSIL (24/24)
Kruskall−wallis test : p= 0.001561
0.0015
0.17
0.0066
7
6
5
4
3
2

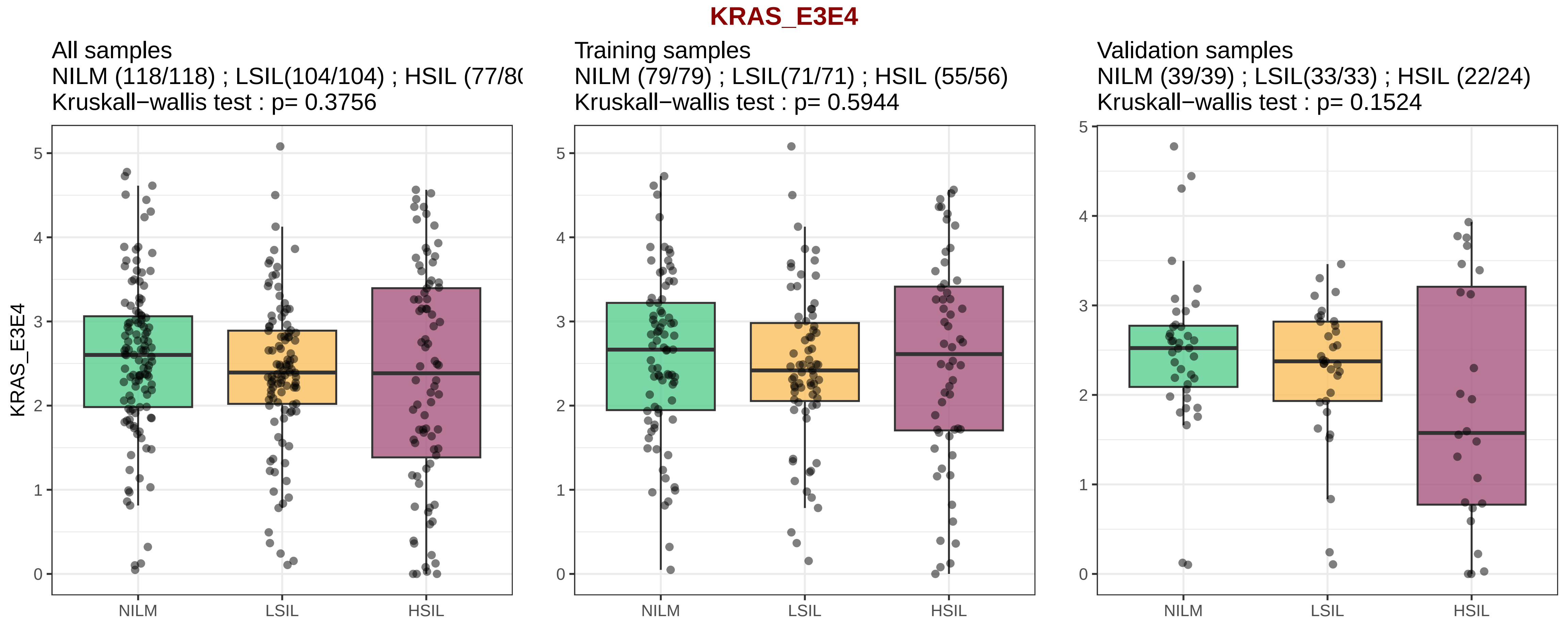

KRAS_E3E4
All samples
NILM (118/118) ; LSIL(104/104) ; HSIL (77/8(
Kruskall−wallis test : p= 0.3756
Training samples
NILM (79/79) ; LSIL(71/71) ; HSIL (55/56)
Kruskall−wallis test : p= 0.5944
Validation samples
NILM (39/39) ; LSIL(33/33) ; HSIL (22/24)
Kruskall−wallis test : p= 0.1524
KRAS_E3E4
0
1
2
3
4
5
NILM
LSIL
HSIL

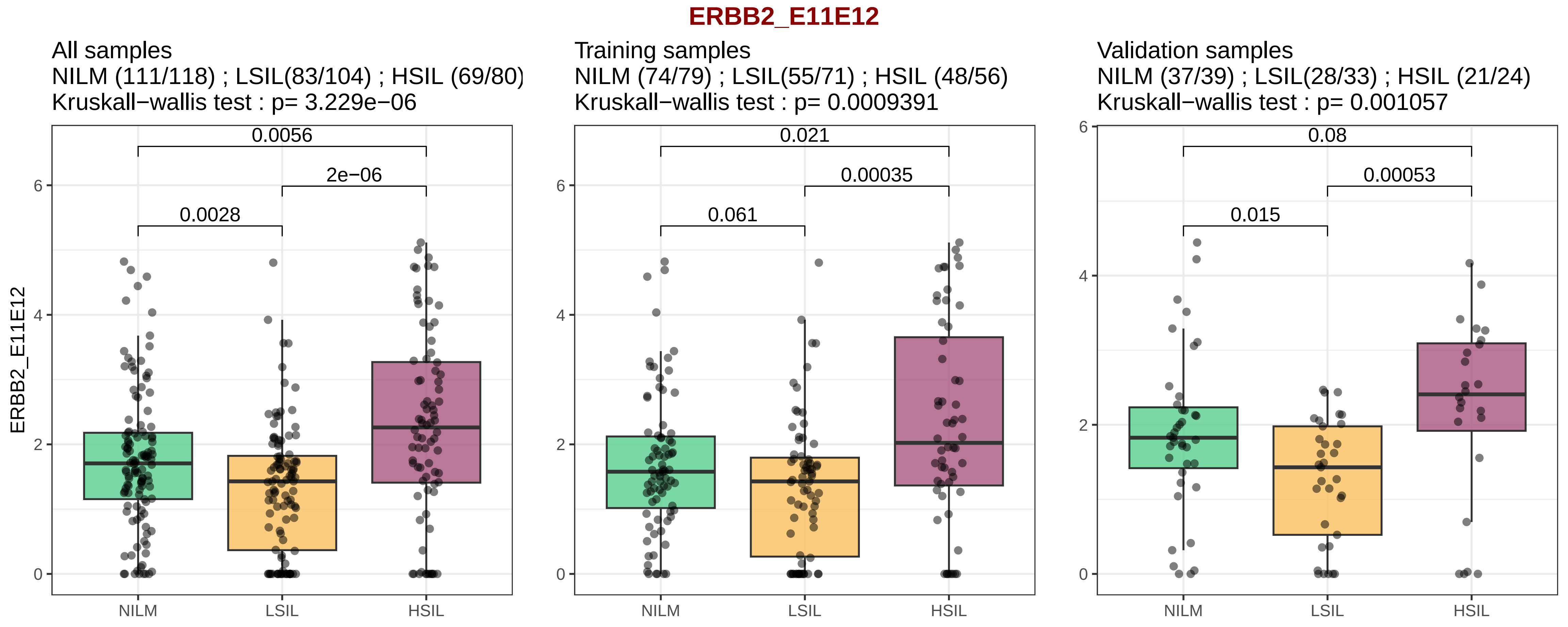

ERBB2_E11E12
All samples
NILM (111/118) ; LSIL(83/104) ; HSIL (69/80)
Kruskall−wallis test : p= 3.229e−06
Training samples
NILM (74/79) ; LSIL(55/71) ; HSIL (48/56)
Kruskall−wallis test : p= 0.0009391
Validation samples
NILM (37/39) ; LSIL(28/33) ; HSIL (21/24)
Kruskall−wallis test : p= 0.001057
ERBB2_E11E12
0.0056
2e−06
0.0028
0.021
0.00035
0.061
0.08
0.00053
0.015
0
2
4
6
NILM
LSIL
HSIL

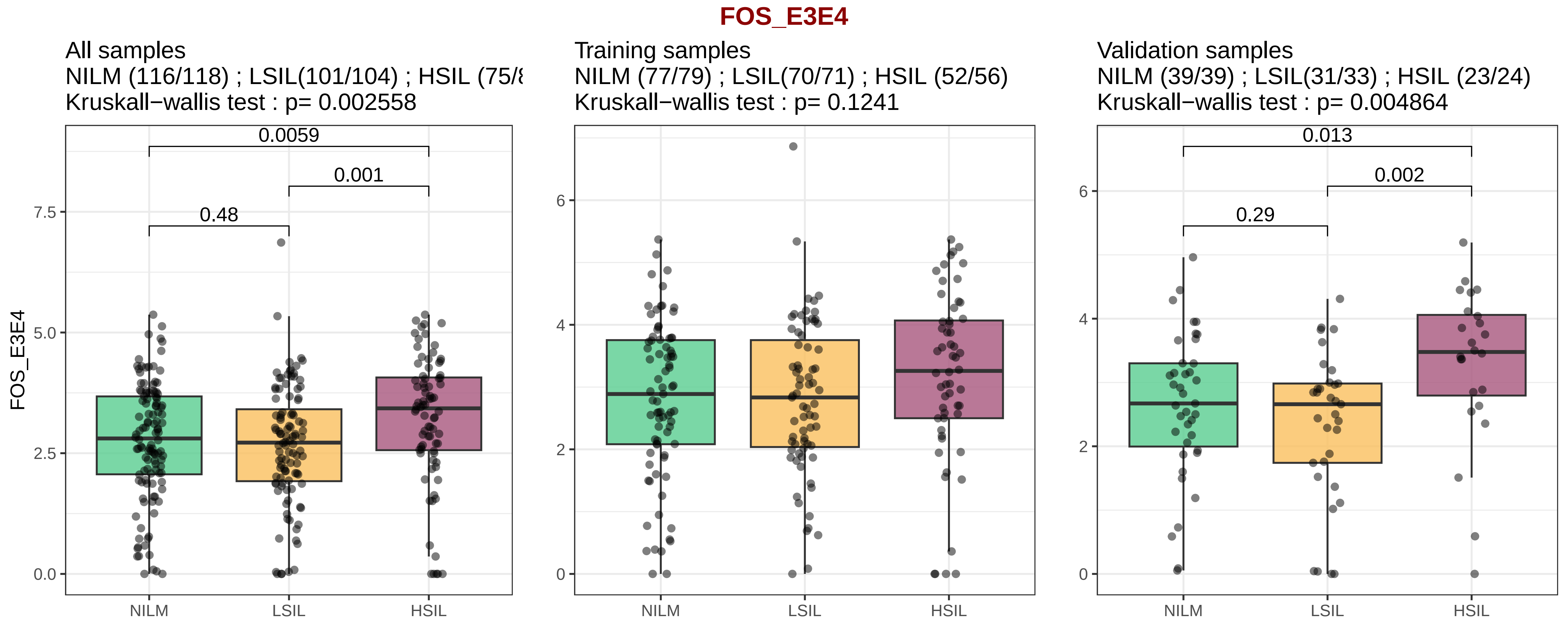
FOS_E3E4
All samples
NILM (116/118) ; LSIL(101/104) ; HSIL (75/8
Kruskall−wallis test : p= 0.002558
Training samples
NILM (77/79) ; LSIL(70/71) ; HSIL (52/56)
Kruskall−wallis test : p= 0.1241
Validation samples
NILM (39/39) ; LSIL(31/33) ; HSIL (23/24)
Kruskall−wallis test : p= 0.004864
0.0059
0.001
0.48
0.013
0.002
0.29
FOS_E3E4
NILM
LSIL
HSIL

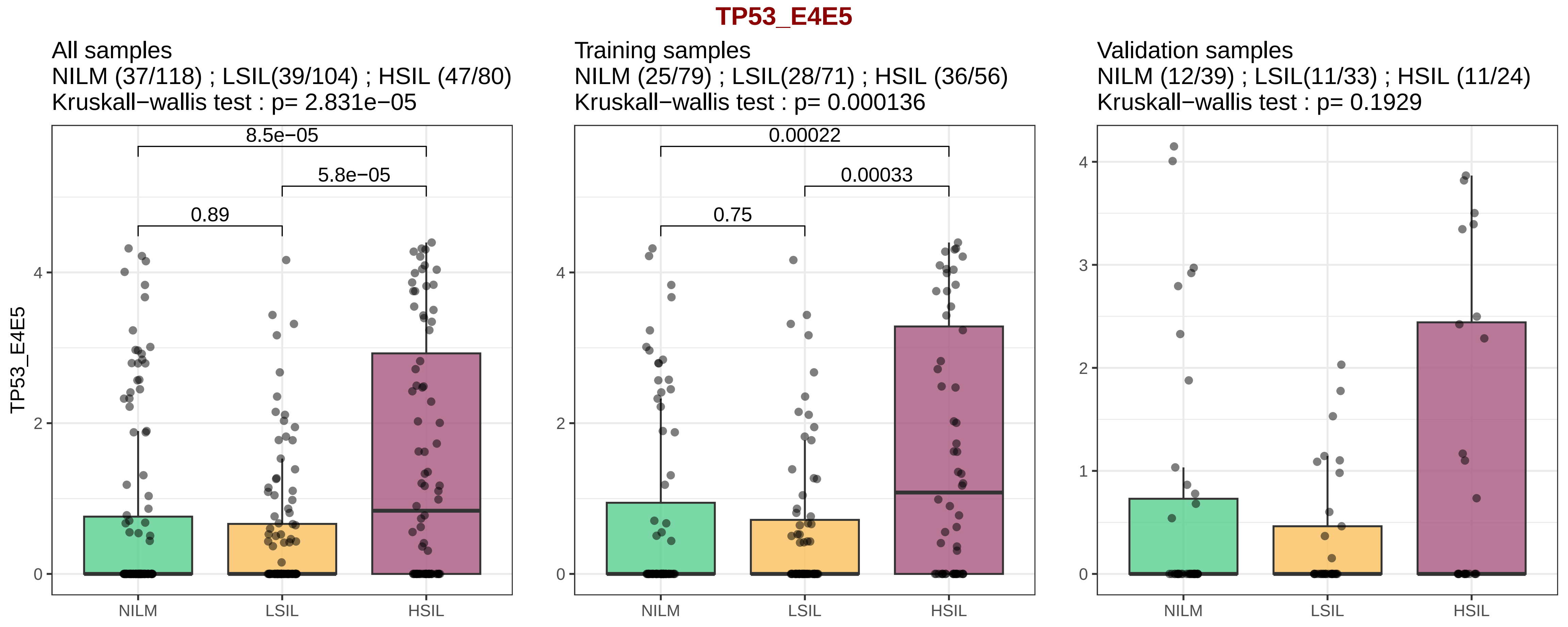

TP53_E4E5
All samples
NILM (37/118) ; LSIL(39/104) ; HSIL (47/80)
Kruskall−wallis test : p= 2.831e−05
8.5e−05
5.8e−05
0.89
TP53_E4E5
0
2
4
NILM
LSIL
HSIL
Training samples
NILM (25/79) ; LSIL(28/71) ; HSIL (36/56)
Kruskall−wallis test : p= 0.000136
0.00022
0.00033
0.75
0
2
4
NILM
LSIL
HSIL
Validation samples
NILM (12/39) ; LSIL(11/33) ; HSIL (11/24)
Kruskall−wallis test : p= 0.1929
0
1
2
3
4
NILM
LSIL
HSIL

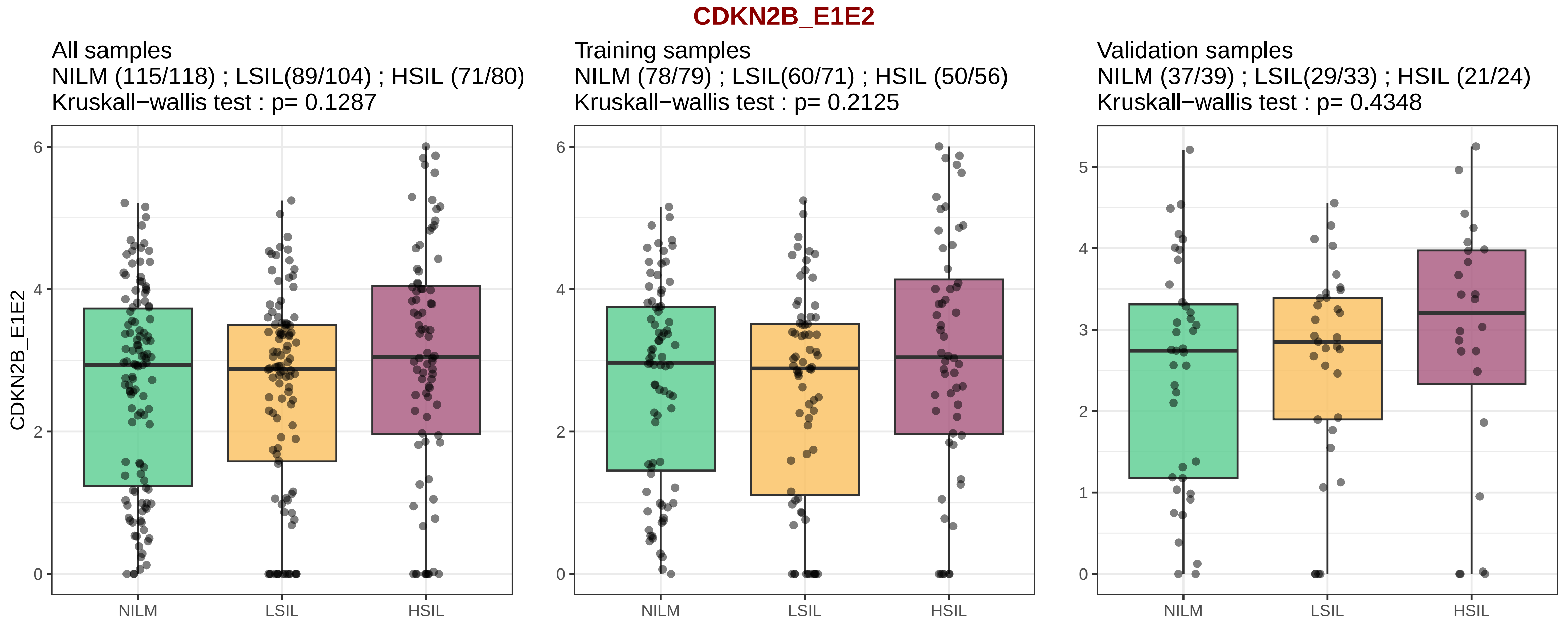
CDKN2B_E1E2
All samples
NILM (115/118) ; LSIL(89/104) ; HSIL (71/80)
Kruskall−wallis test : p= 0.1287
Training samples
NILM (78/79) ; LSIL(60/71) ; HSIL (50/56)
Kruskall−wallis test : p= 0.2125
Validation samples
NILM (37/39) ; LSIL(29/33) ; HSIL (21/24)
Kruskall−wallis test : p= 0.4348
CDKN2B_E1E2
NILM
LSIL
HSIL

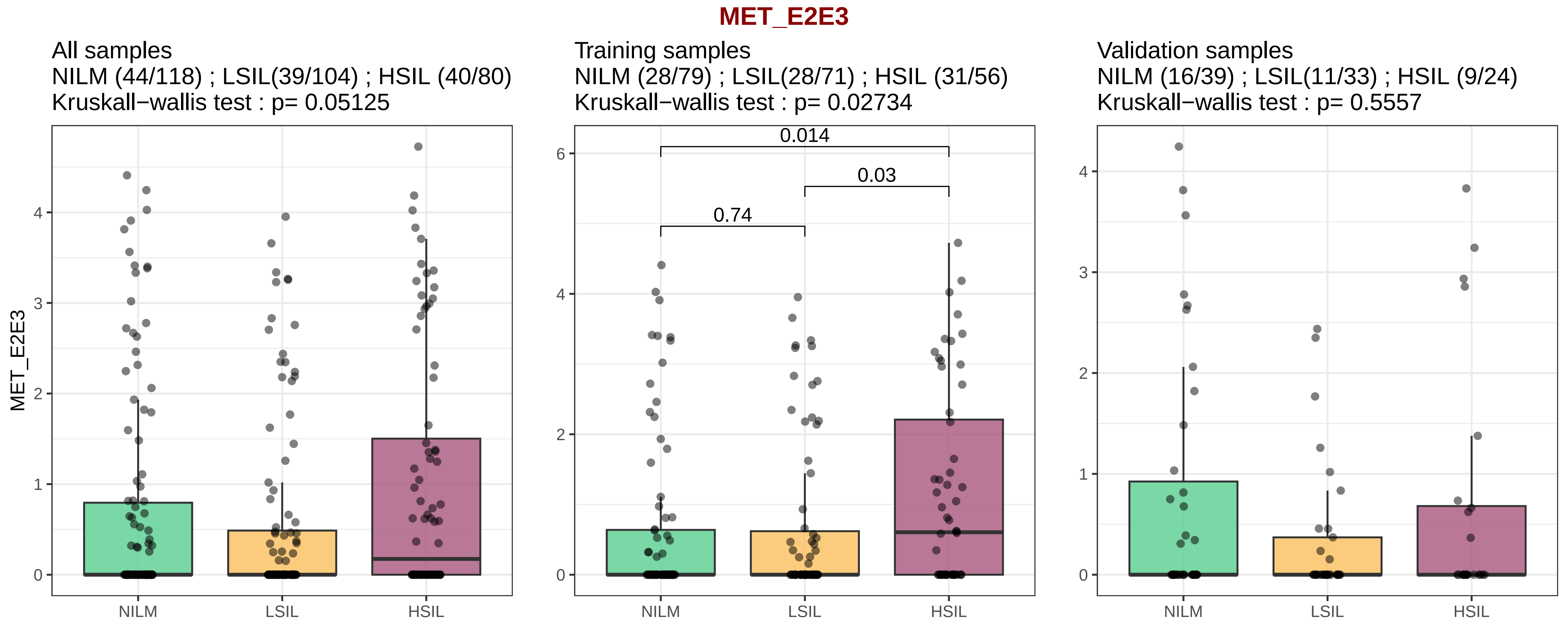

MET_E2E3
All samples
NILM (44/118) ; LSIL(39/104) ; HSIL (40/80)
Kruskall−wallis test : p= 0.05125
Training samples
NILM (28/79) ; LSIL(28/71) ; HSIL (31/56)
Kruskall−wallis test : p= 0.02734
Validation samples
NILM (16/39) ; LSIL(11/33) ; HSIL (9/24)
Kruskall−wallis test : p= 0.5557
MET_E2E3
0.014
0.03
0.74
NILM
LSIL
HSIL

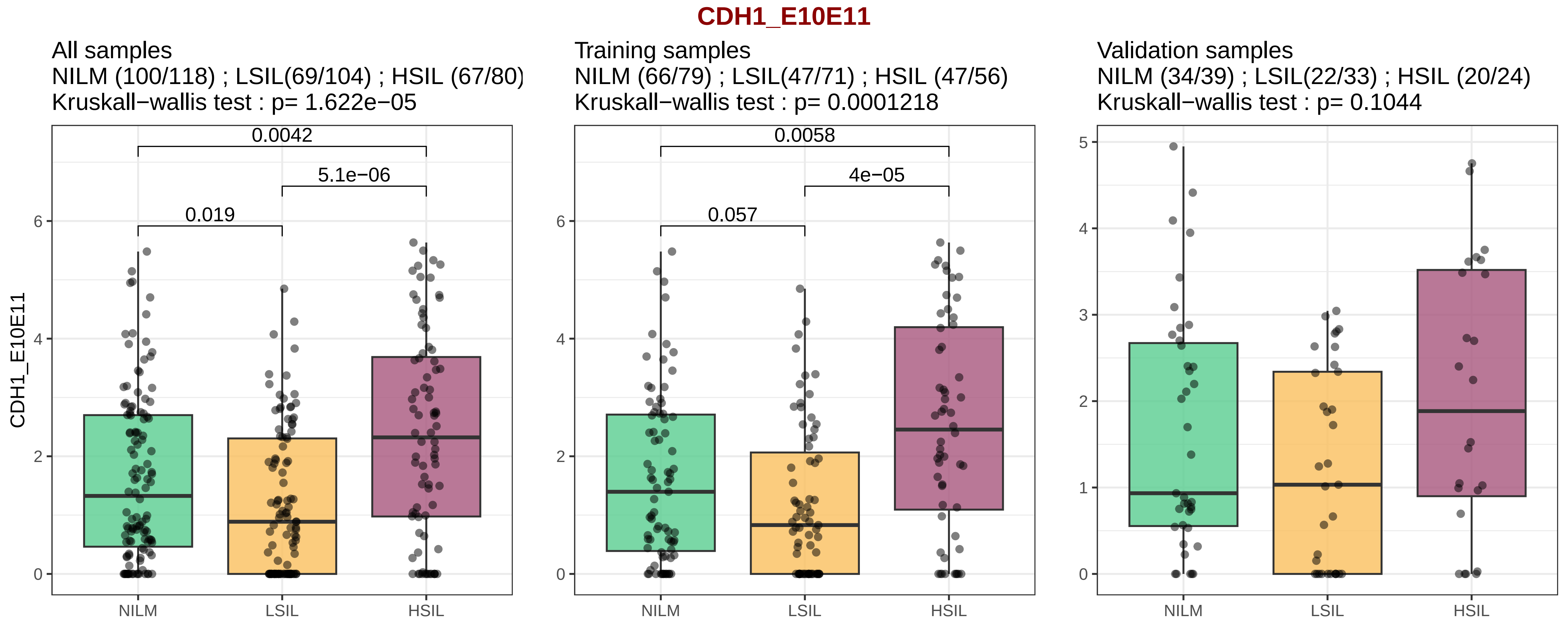
CDH1_E10E11
All samples
NILM (100/118) ; LSIL(69/104) ; HSIL (67/80)
Kruskall−wallis test : p= 1.622e−05
0.0042
5.1e−06
0.019
Training samples
NILM (66/79) ; LSIL(47/71) ; HSIL (47/56)
Kruskall−wallis test : p= 0.0001218
0.0058
4e−05
0.057
Validation samples
NILM (34/39) ; LSIL(22/33) ; HSIL (20/24)
Kruskall−wallis test : p= 0.1044
CDH1_E10E11
NILM
LSIL
HSIL
0
2
4
6
0
1
2
3
4
5

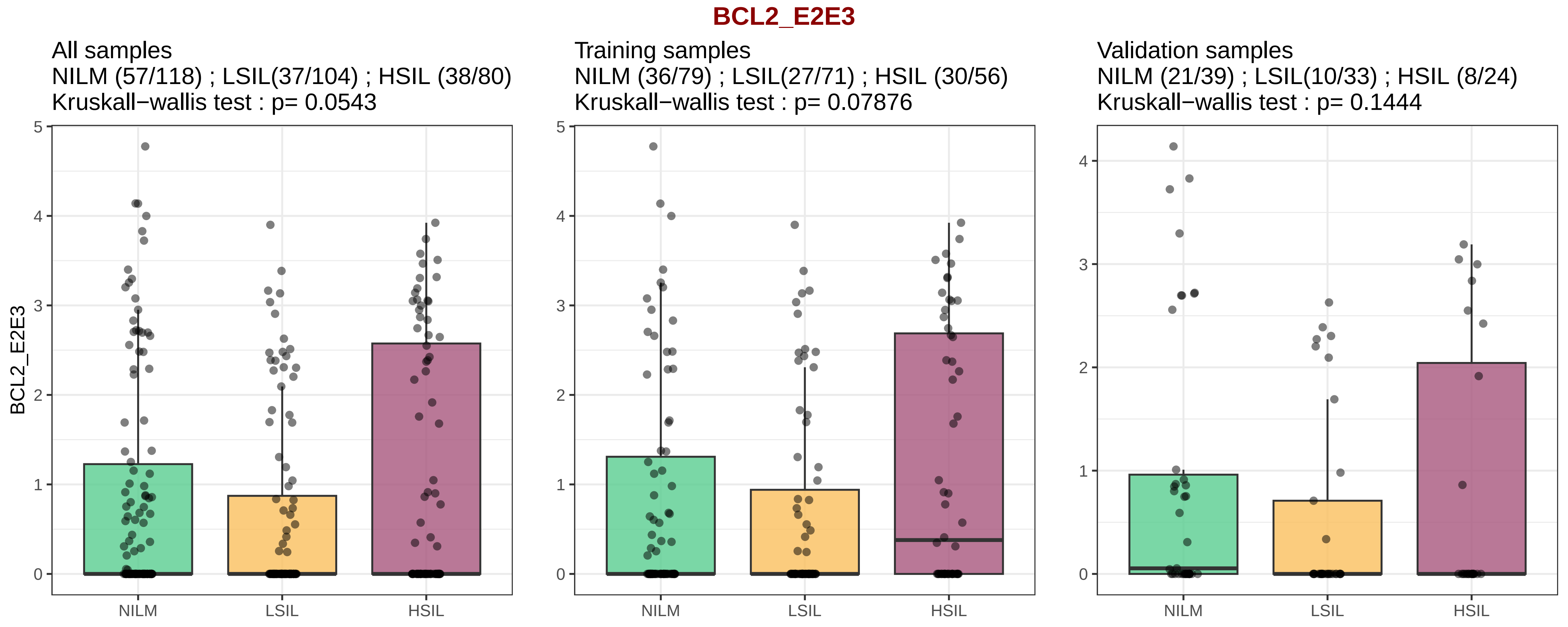
BCL2_E2E3
All samples
NILM (57/118) ; LSIL(37/104) ; HSIL (38/80)
Kruskall−wallis test : p= 0.0543
Training samples
NILM (36/79) ; LSIL(27/71) ; HSIL (30/56)
Kruskall−wallis test : p= 0.07876
Validation samples
NILM (21/39) ; LSIL(10/33) ; HSIL (8/24)
Kruskall−wallis test : p= 0.1444
BCL2_E2E3
NILM
LSIL
HSIL

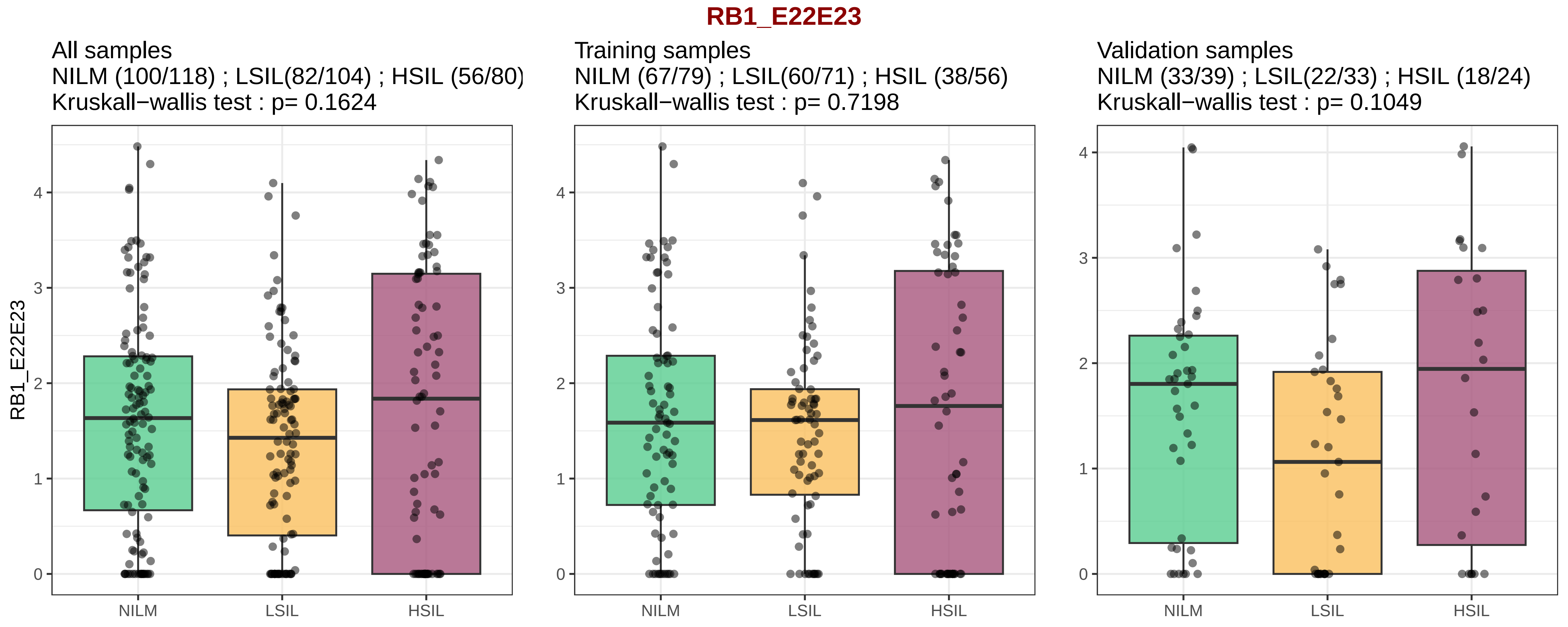
RB1_E22E23
All samples
NILM (100/118) ; LSIL(82/104) ; HSIL (56/80)
Kruskall−wallis test : p= 0.1624
Training samples
NILM (67/79) ; LSIL(60/71) ; HSIL (38/56)
Kruskall−wallis test : p= 0.7198
Validation samples
NILM (33/39) ; LSIL(22/33) ; HSIL (18/24)
Kruskall−wallis test : p= 0.1049
RB1_E22E23
0
1
2
3
4
NILM
LSIL
HSIL

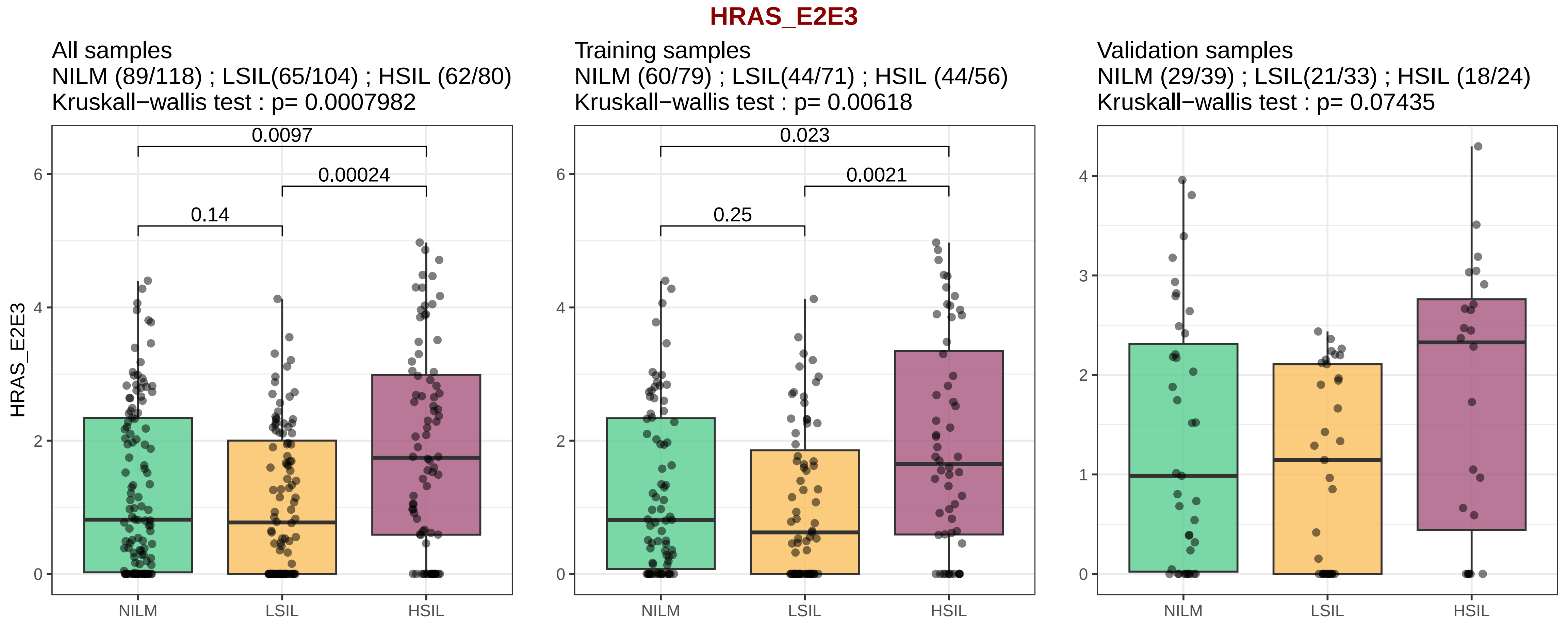

HRAS_E2E3
All samples
NILM (89/118) ; LSIL(65/104) ; HSIL (62/80)
Kruskall−wallis test : p= 0.0007982
0.0097
0.00024
0.14
HRAS_E2E3
0
2
4
6
NILM
LSIL
HSIL
Training samples
NILM (60/79) ; LSIL(44/71) ; HSIL (44/56)
Kruskall−wallis test : p= 0.00618
0.023
0.0021
0.25
0
2
4
6
NILM
LSIL
HSIL
Validation samples
NILM (29/39) ; LSIL(21/33) ; HSIL (18/24)
Kruskall−wallis test : p= 0.07435
0
1
2
3
4
NILM
LSIL
HSIL

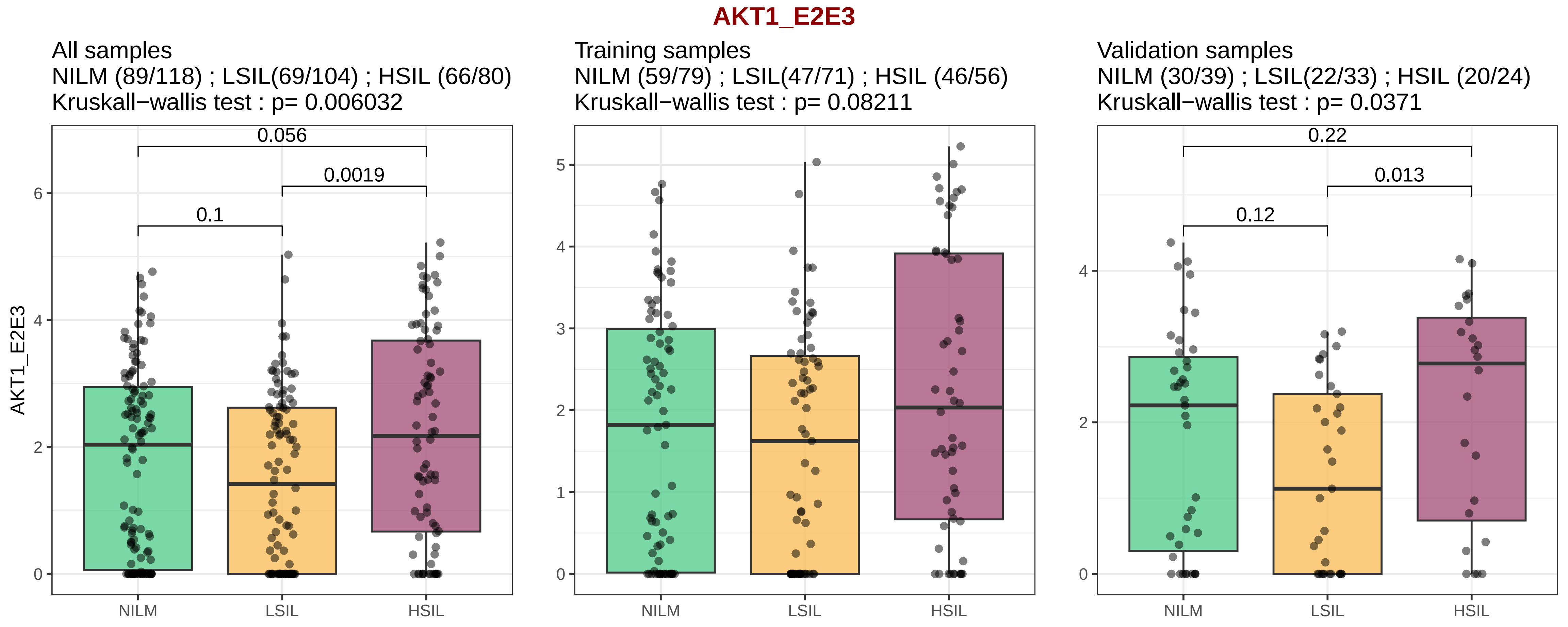

AKT1_E2E3
All samples
NILM (89/118) ; LSIL(69/104) ; HSIL (66/80)
Kruskall−wallis test : p= 0.006032
0.056
0.0019
0.1
AKT1_E2E3
0
2
4
6
NILM
LSIL
HSIL
Training samples
NILM (59/79) ; LSIL(47/71) ; HSIL (46/56)
Kruskall−wallis test : p= 0.08211
0
1
2
3
4
5
NILM
LSIL
HSIL
Validation samples
NILM (30/39) ; LSIL(22/33) ; HSIL (20/24)
Kruskall−wallis test : p= 0.0371
0.22
0.013
0.12
0
2
4
NILM
LSIL
HSIL

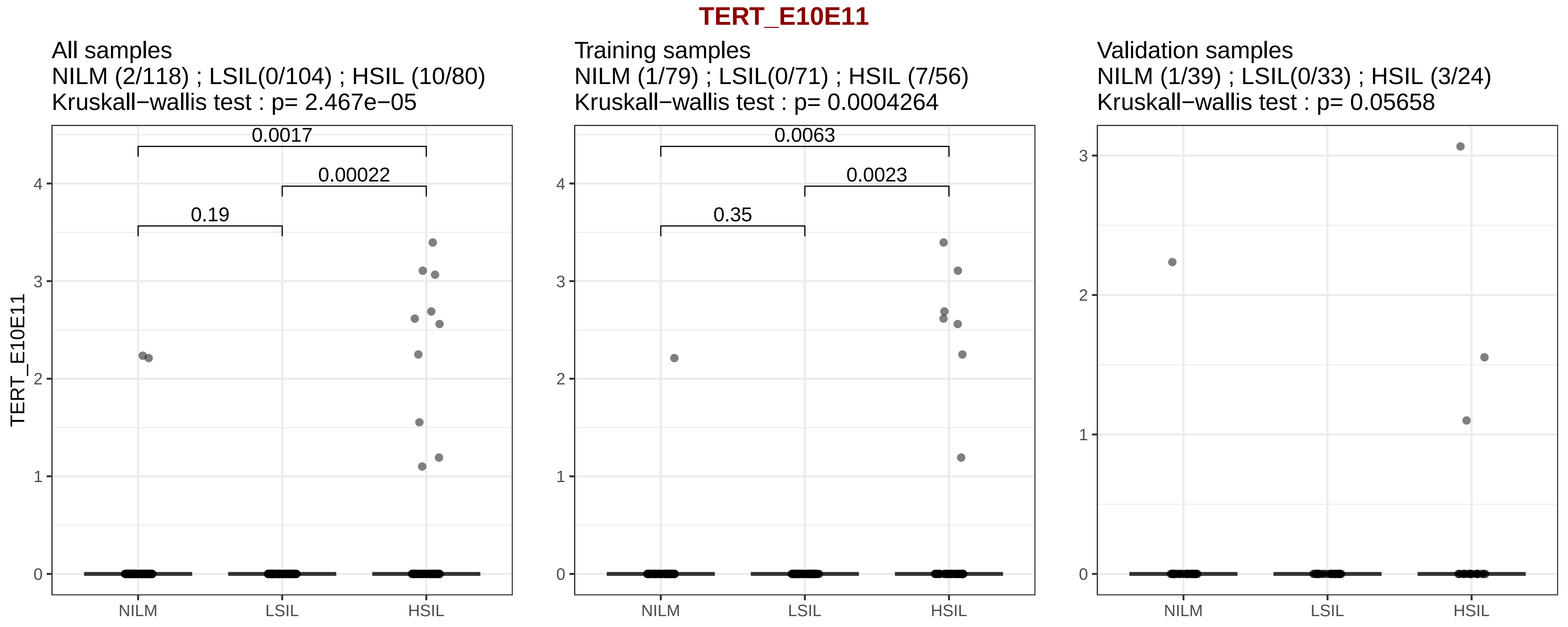
TERT_E10E11
All samples
NILM (2/118) ; LSIL(0/104) ; HSIL (10/80)
Kruskall−wallis test : p= 2.467e−05
0.0017
0.00022
0.19
TERT_E10E11
0
1
2
3
4
NILM
LSIL
HSIL
Training samples
NILM (1/79) ; LSIL(0/71) ; HSIL (7/56)
Kruskall−wallis test : p= 0.0004264
0.0063
0.0023
0.35
Validation samples
NILM (1/39) ; LSIL(0/33) ; HSIL (3/24)
Kruskall−wallis test : p= 0.05658

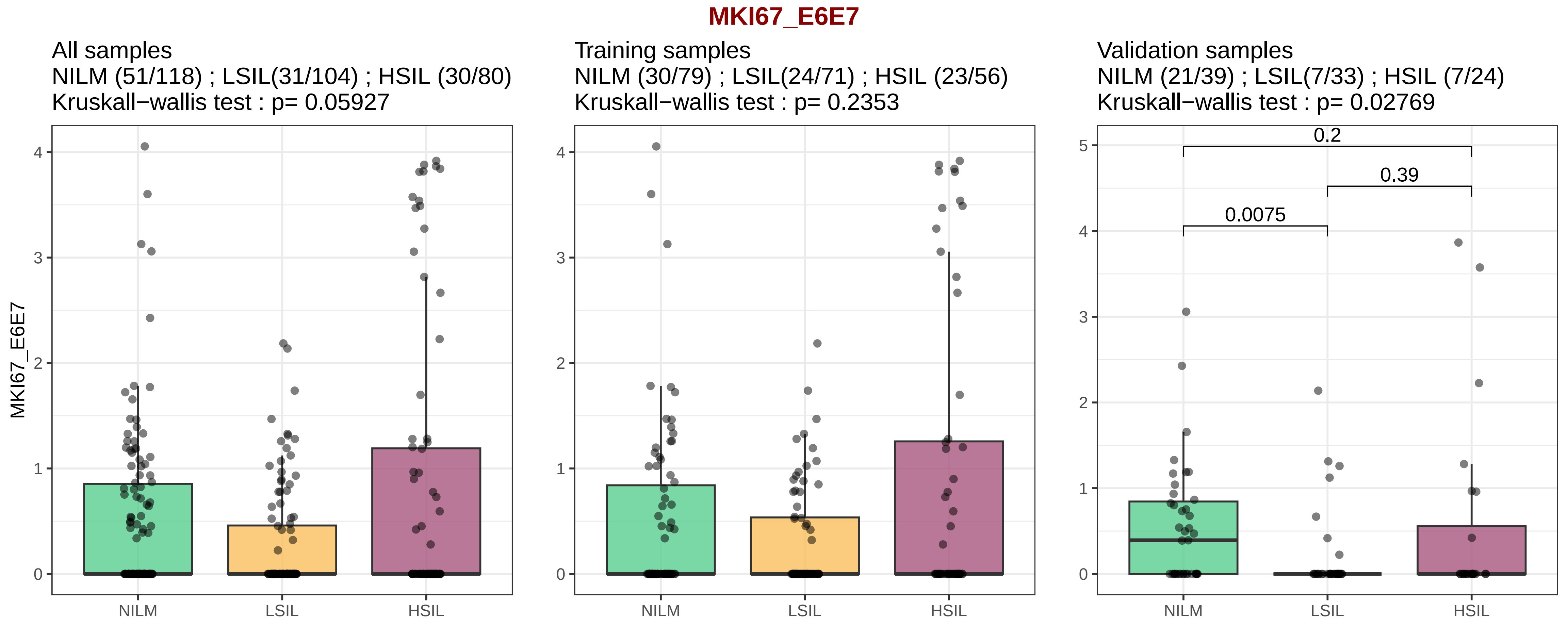
MKI67_E6E7
All samples
NILM (51/118) ; LSIL(31/104) ; HSIL (30/80)
Kruskall−wallis test : p= 0.05927
Training samples
NILM (30/79) ; LSIL(24/71) ; HSIL (23/56)
Kruskall−wallis test : p= 0.2353
Validation samples
NILM (21/39) ; LSIL(7/33) ; HSIL (7/24)
Kruskall−wallis test : p= 0.02769
MKI67_E6E7
0.2
0.39
0.0075
NILM
LSIL
HSIL

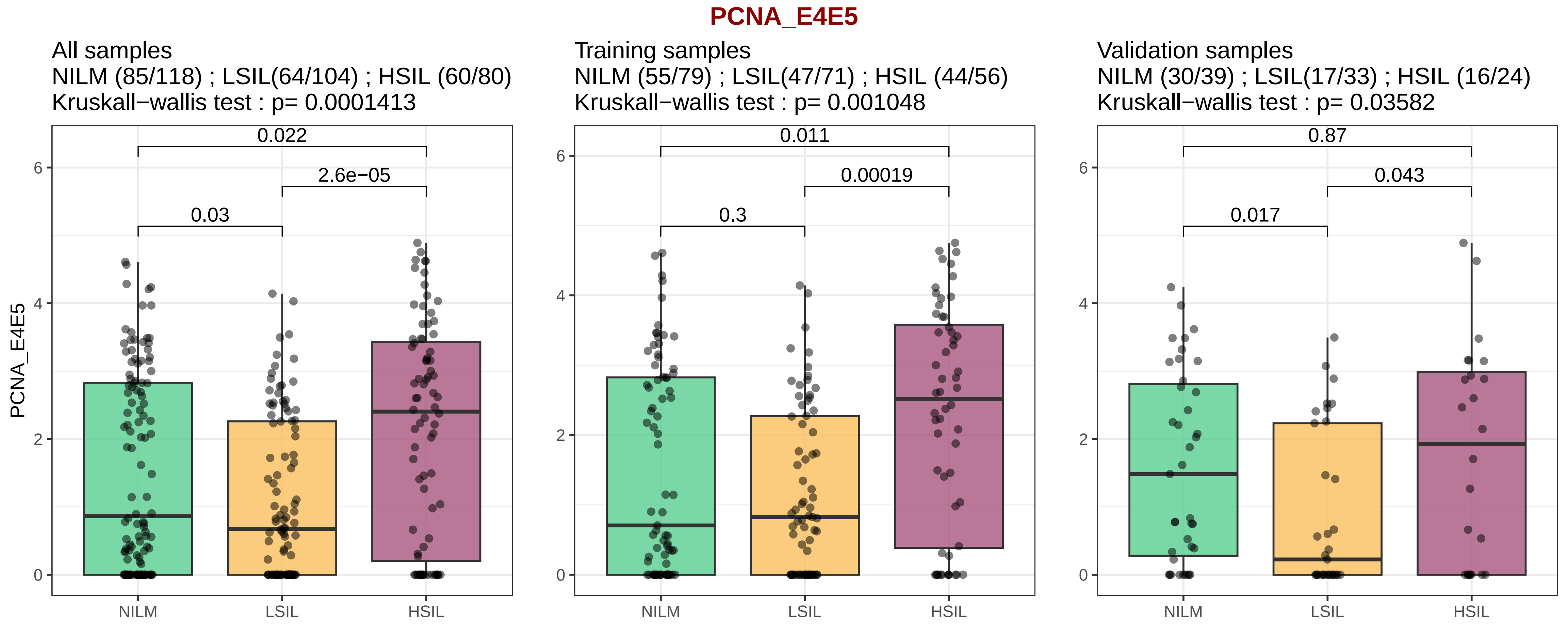
PCNA_E4E5
All samples
NILM (85/118) ; LSIL(64/104) ; HSIL (60/80)
Kruskall−wallis test : p= 0.0001413
0.022
2.6e−05
0.03
Training samples
NILM (55/79) ; LSIL(47/71) ; HSIL (44/56)
Kruskall−wallis test : p= 0.001048
0.011
0.00019
0.3
Validation samples
NILM (30/39) ; LSIL(17/33) ; HSIL (16/24)
Kruskall−wallis test : p= 0.03582
0.87
0.043
0.017
PCNA_E4E5
0
2
4
6
NILM
LSIL
HSIL

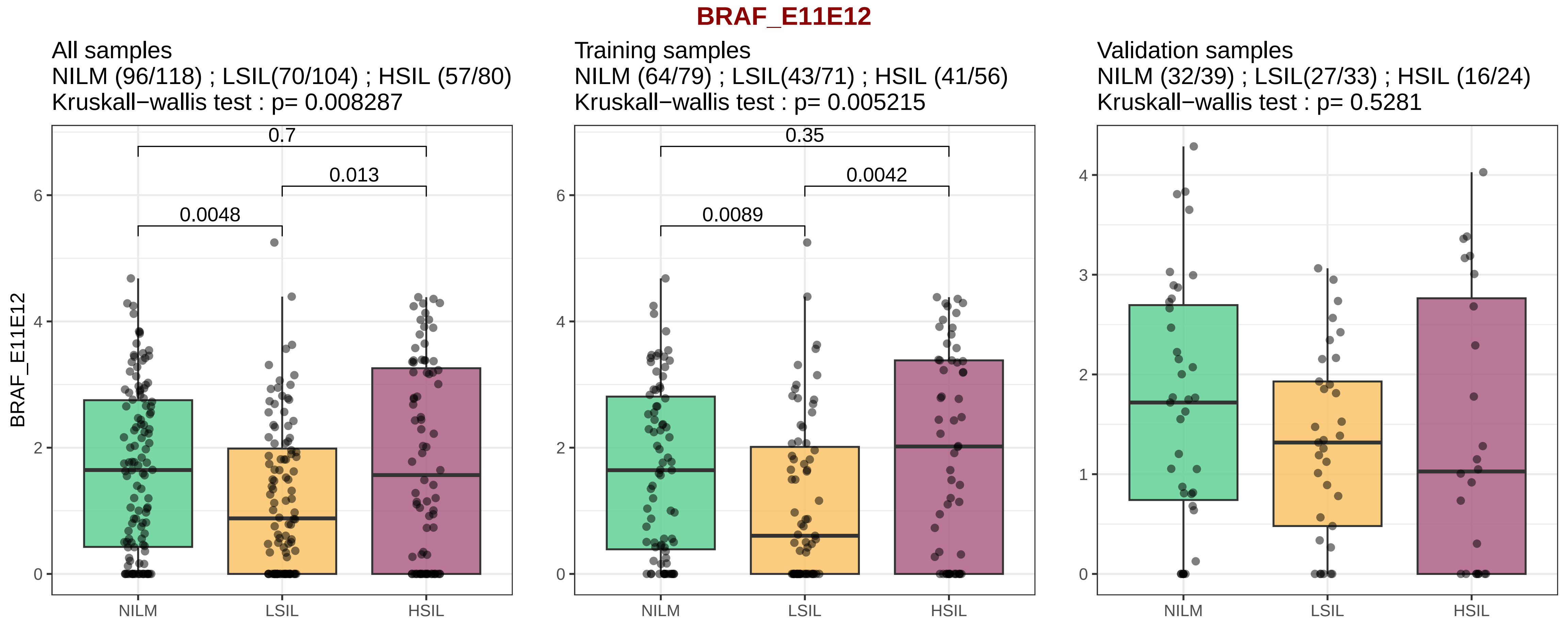

BRAF_E11E12
All samples
NILM (96/118) ; LSIL(70/104) ; HSIL (57/80)
Kruskall−wallis test : p= 0.008287
0.7
0.013
0.0048
BRAF_E11E12
0
2
4
6
NILM
LSIL
HSIL
Training samples
NILM (64/79) ; LSIL(43/71) ; HSIL (41/56)
Kruskall−wallis test : p= 0.005215
0.35
0.0042
0.0089
0
2
4
6
NILM
LSIL
HSIL
Validation samples
NILM (32/39) ; LSIL(27/33) ; HSIL (16/24)
Kruskall−wallis test : p= 0.5281
0
1
2
3
4
NILM
LSIL
HSIL

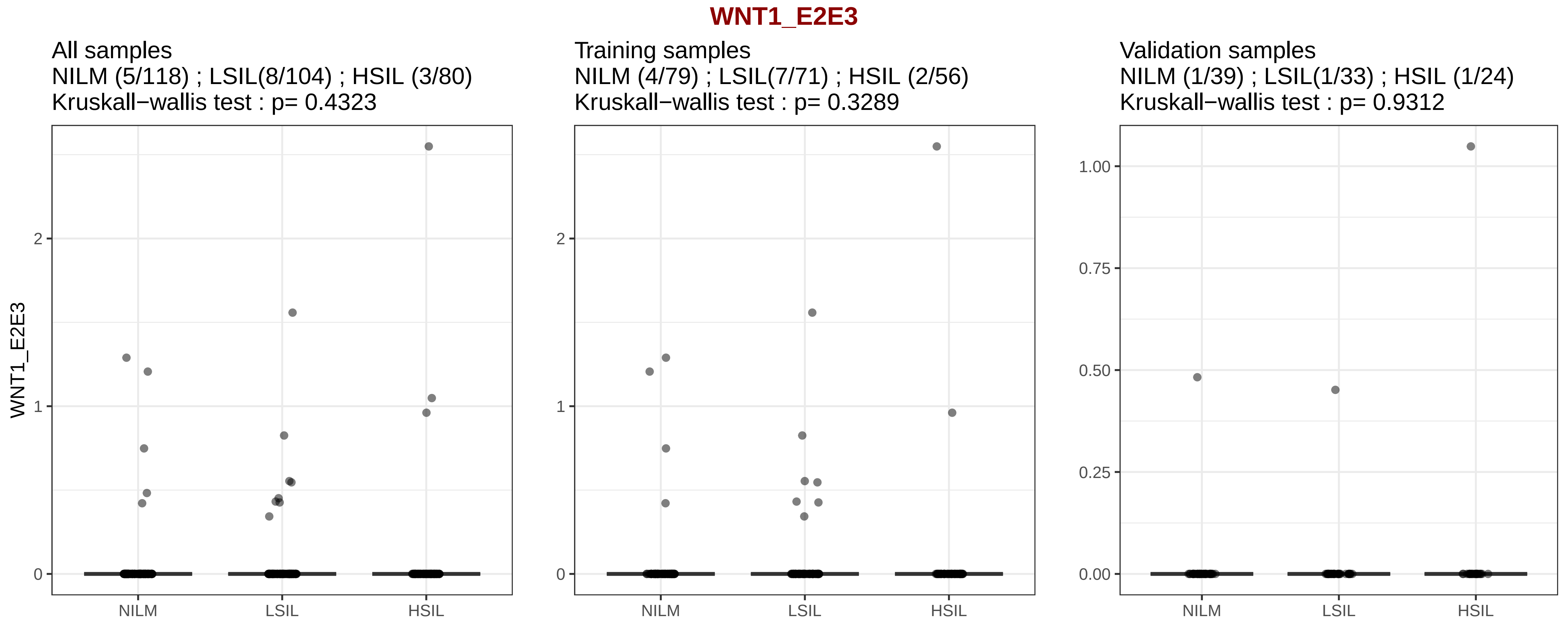
WNT1_E2E3
All samples
NILM (5/118) ; LSIL(8/104) ; HSIL (3/80)
Kruskall−wallis test : p= 0.4323
Training samples
NILM (4/79) ; LSIL(7/71) ; HSIL (2/56)
Kruskall−wallis test : p= 0.3289
Validation samples
NILM (1/39) ; LSIL(1/33) ; HSIL (1/24)
Kruskall−wallis test : p= 0.9312
WNT1_E2E3
0
1
2
1.00
0.75
0.50
0.25
0.00
NILM
LSIL
HSIL

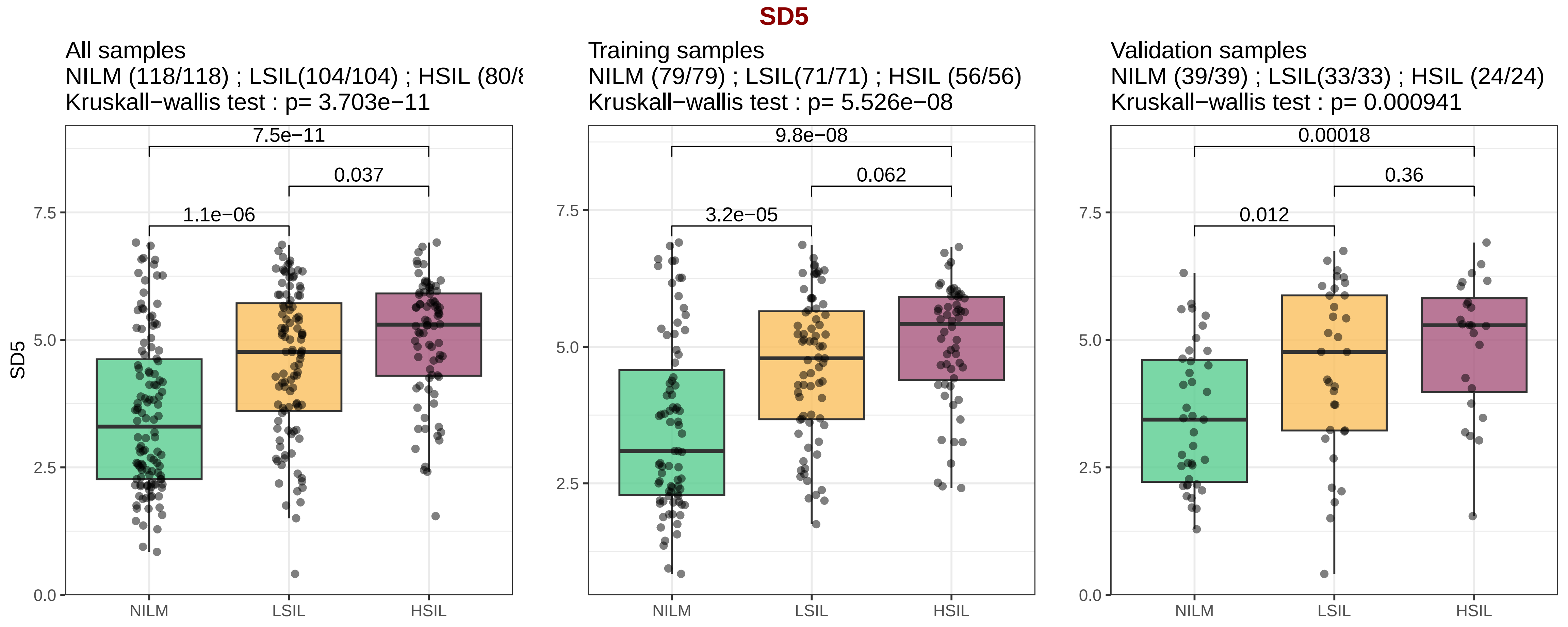
SD5
All samples
NILM (118/118) ; LSIL(104/104) ; HSIL (80/8
Kruskall−wallis test : p= 3.703e−11
7.5e−11
0.037
1.1e−06
Training samples
NILM (79/79) ; LSIL(71/71) ; HSIL (56/56)
Kruskall−wallis test : p= 5.526e−08
9.8e−08
0.062
3.2e−05
Validation samples
NILM (39/39) ; LSIL(33/33) ; HSIL (24/24)
Kruskall−wallis test : p= 0.000941
0.00018
0.36
0.012
SD5
0.0
2.5
5.0
7.5
NILM
LSIL
HSIL

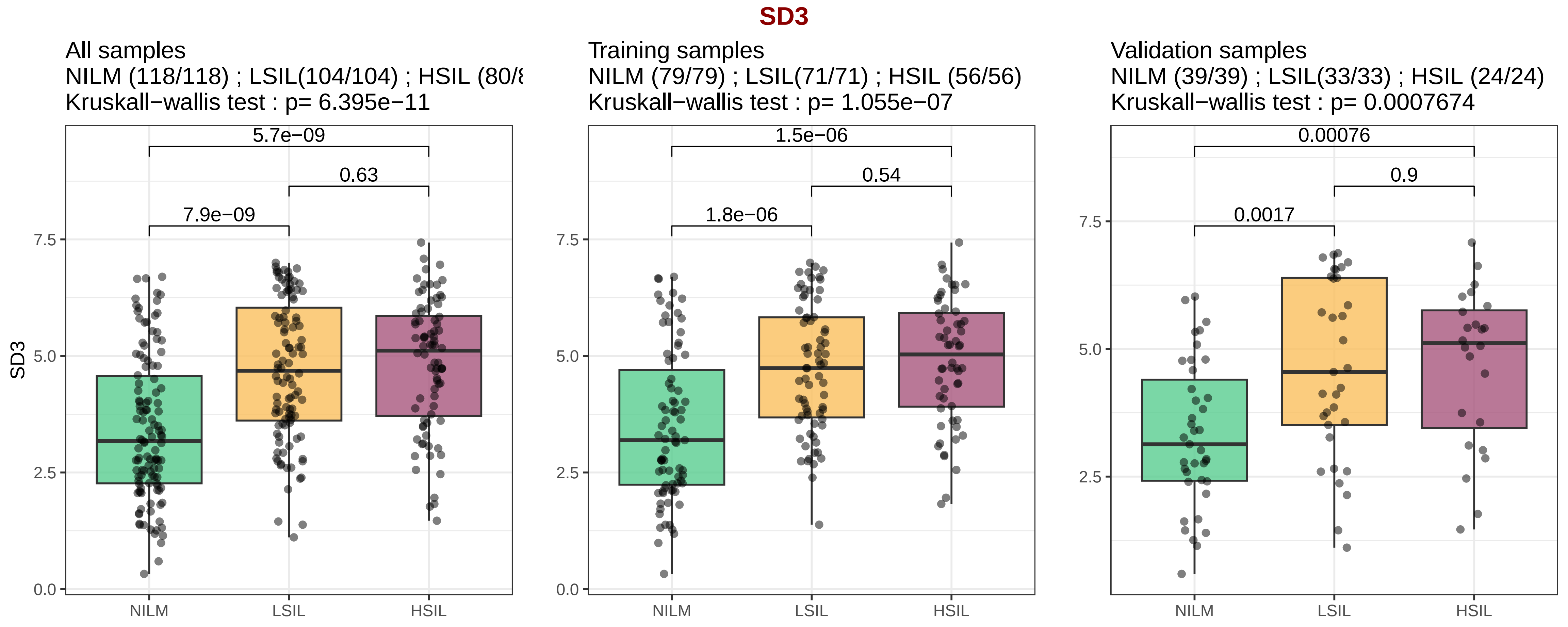

SD3
All samples
NILM (118/118) ; LSIL(104/104) ; HSIL (80/8
Kruskall−wallis test : p= 6.395e−11
5.7e−09
0.63
7.9e−09
Training samples
NILM (79/79) ; LSIL(71/71) ; HSIL (56/56)
Kruskall−wallis test : p= 1.055e−07
1.5e−06
0.54
1.8e−06
Validation samples
NILM (39/39) ; LSIL(33/33) ; HSIL (24/24)
Kruskall−wallis test : p= 0.0007674
0.00076
0.9
0.0017
SD3
0.0
2.5
5.0
7.5
NILM
LSIL
HSIL

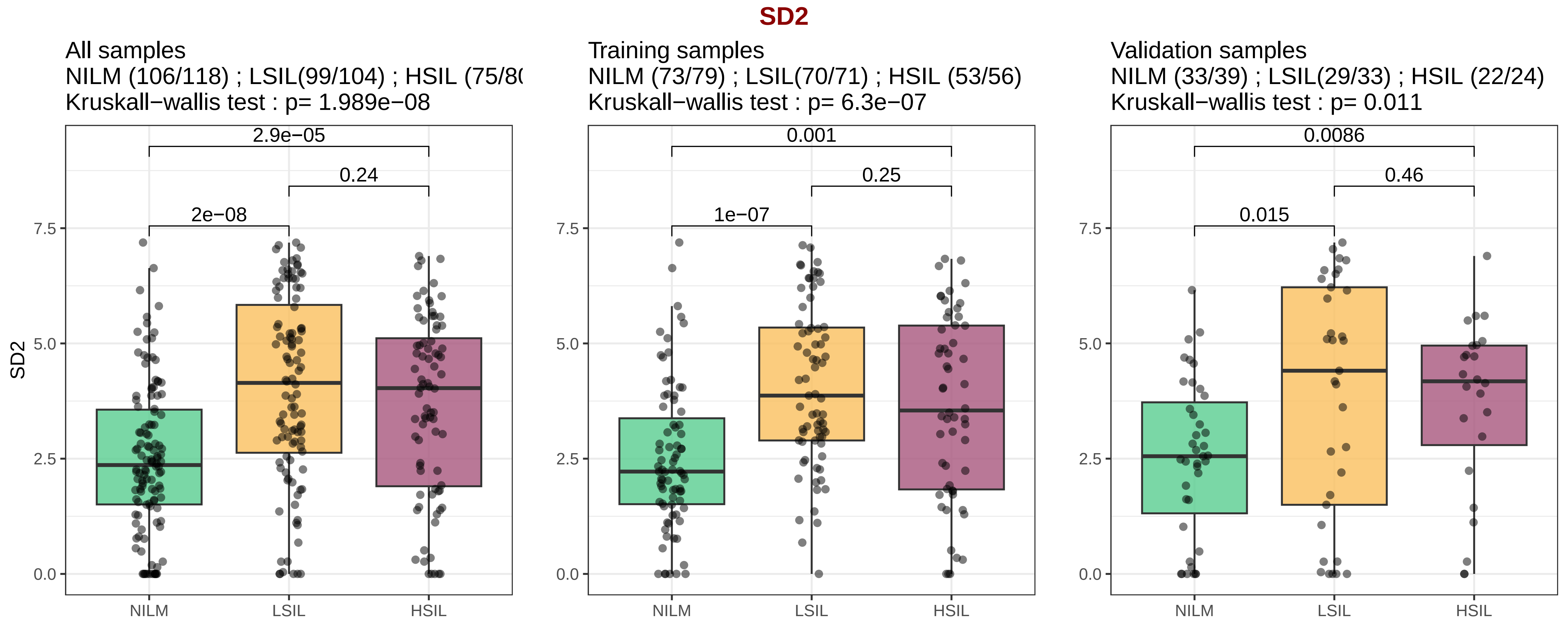

SD2
All samples
NILM (106/118) ; LSIL(99/104) ; HSIL (75/8(
Kruskall−wallis test : p= 1.989e−08
2.9e−05
0.24
2e−08
Training samples
NILM (73/79) ; LSIL(70/71) ; HSIL (53/56)
Kruskall−wallis test : p= 6.3e−07
0.001
0.25
1e−07
Validation samples
NILM (33/39) ; LSIL(29/33) ; HSIL (22/24)
Kruskall−wallis test : p= 0.011
0.0086
0.46
0.015
SD2
0.0
2.5
5.0
7.5
NILM
LSIL
HSIL

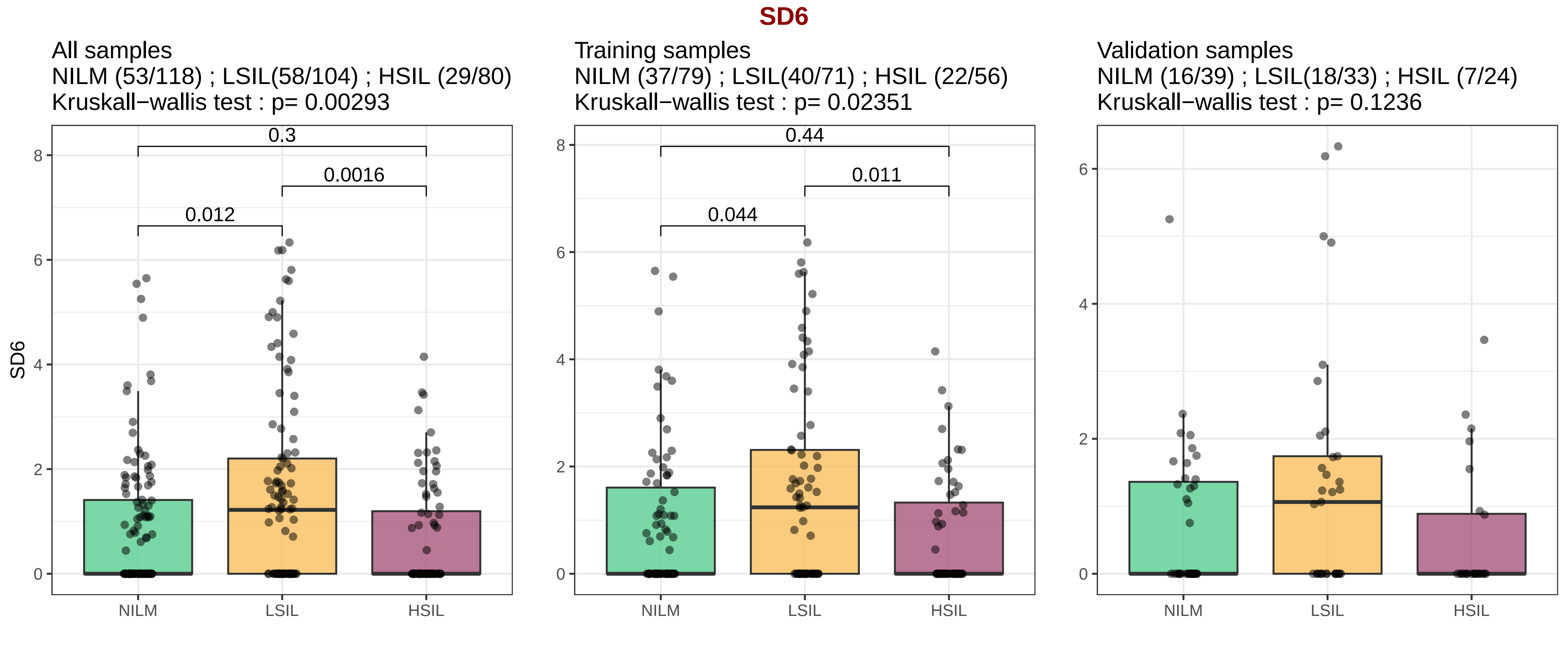
SD6
All samples
NILM (53/118) ; LSIL(58/104) ; HSIL (29/80)
Kruskall−wallis test : p= 0.00293
0.3
0.0016
0.012
Training samples
NILM (37/79) ; LSIL(40/71) ; HSIL (22/56)
Kruskall−wallis test : p= 0.02351
0.44
0.011
0.044
Validation samples
NILM (16/39) ; LSIL(18/33) ; HSIL (7/24)
Kruskall−wallis test : p= 0.1236
SD6
NILM
LSIL
HSIL

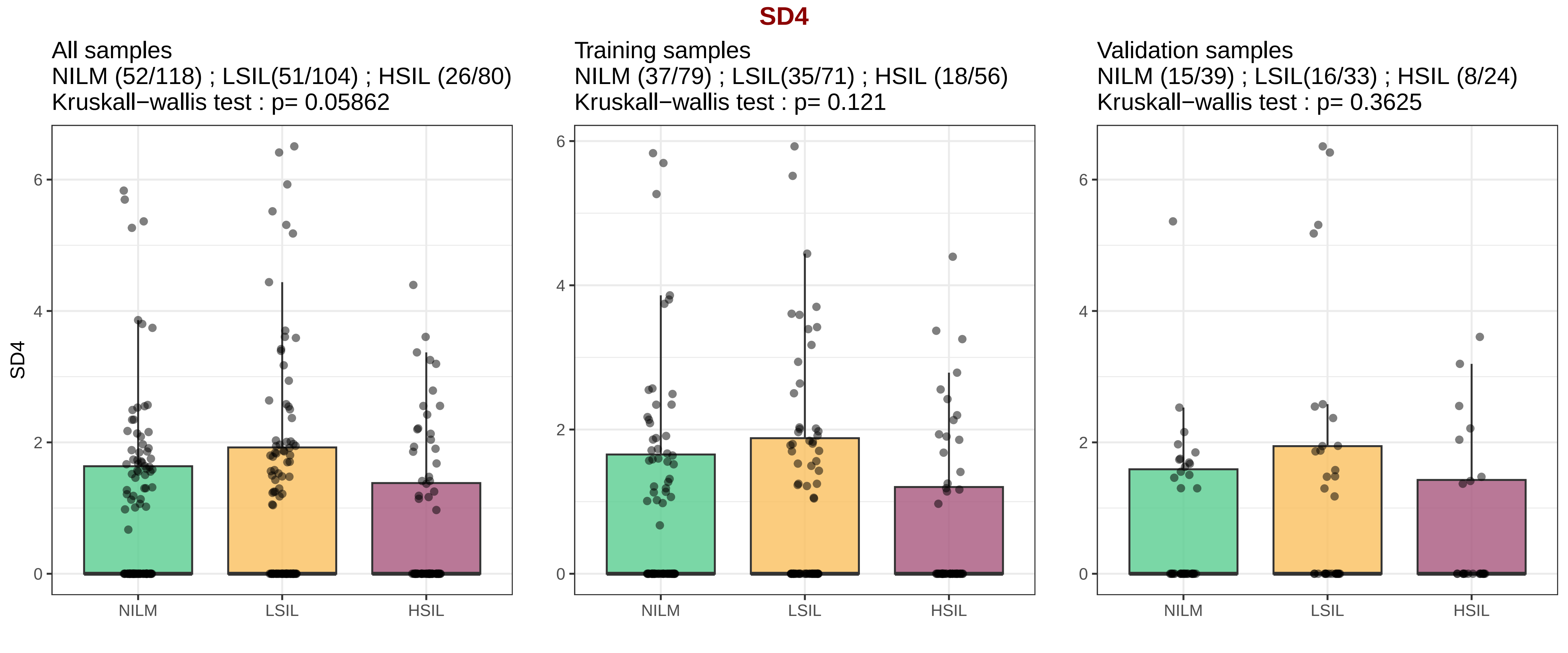
SD4
All samples
NILM (52/118) ; LSIL(51/104) ; HSIL (26/80)
Kruskall−wallis test : p= 0.05862
Training samples
NILM (37/79) ; LSIL(35/71) ; HSIL (18/56)
Kruskall−wallis test : p= 0.121
Validation samples
NILM (15/39) ; LSIL(16/33) ; HSIL (8/24)
Kruskall−wallis test : p= 0.3625
SD4
0
2
4
6
NILM
LSIL
HSIL

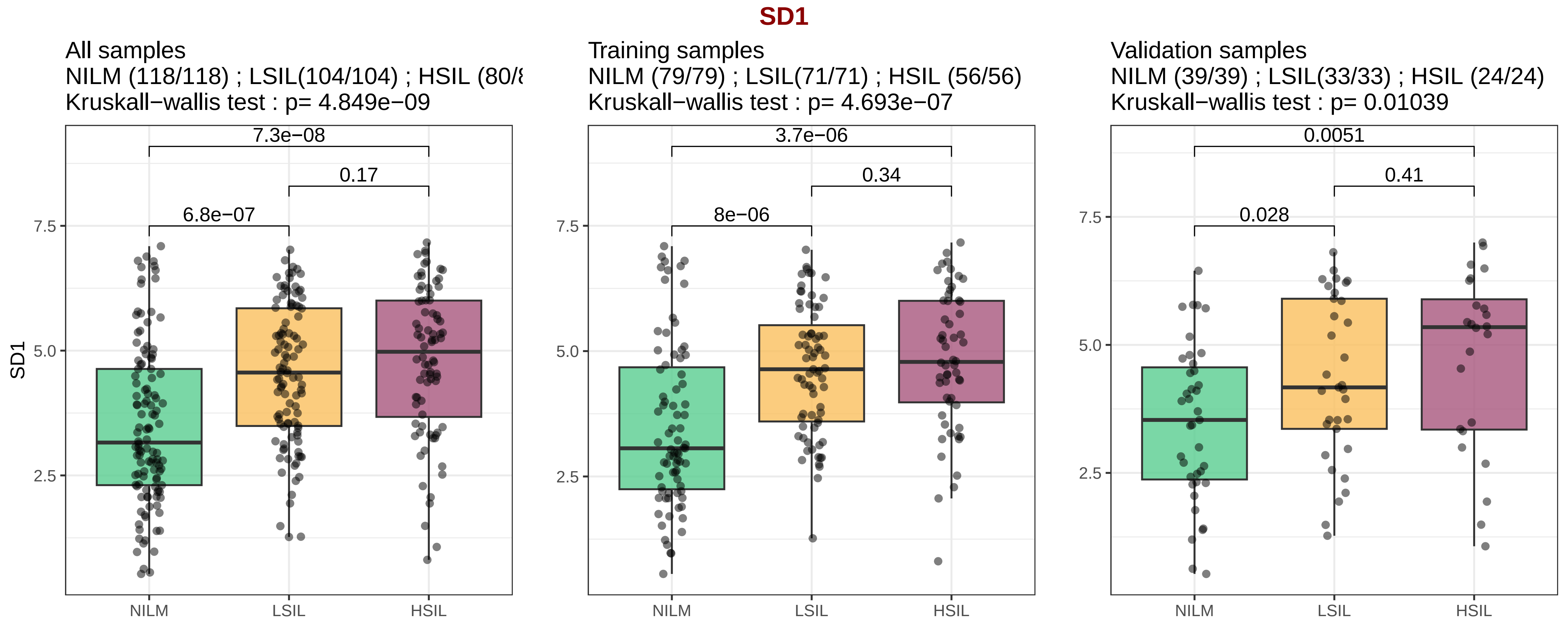
SD1
All samples
NILM (118/118) ; LSIL(104/104) ; HSIL (80/8
Kruskall−wallis test : p= 4.849e−09
7.3e−08
0.17
6.8e−07
Training samples
NILM (79/79) ; LSIL(71/71) ; HSIL (56/56)
Kruskall−wallis test : p= 4.693e−07
3.7e−06
0.34
8e−06
Validation samples
NILM (39/39) ; LSIL(33/33) ; HSIL (24/24)
Kruskall−wallis test : p= 0.01039
0.0051
0.41
0.028
SD1
7.5
5.0
2.5
NILM
LSIL
HSIL
