# Supplementary material for: Molecular detection of hrHPV-induced high-grade squamous intraepithelial lesions of the cervix through a targeted RNA next generation sequencing assay
Source: Mol Med. 2025 May 30;31:215. doi: 10.1186/s10020-025-01238-x (PMC12125924; doi:10.1186/s10020-025-01238-x)
Supplement: Supplementary file 5 — Supplementary Material 5: SuppData 5. [file 10020_2025_1238_MOESM5_ESM.pdf]

Positive Predictive Value

1. Positive Predictive Value: basic definition

The Positive Predictive value is defined as the proportion of True Positive predictions (TP) among the total number of positive predictions, that is $TP + FP$, where FP, is the number of False Positives.

$$PPV = \frac{TP}{TP + FP} \tag{1}$$

The Positive Predictive Value may also be obtained based on Sensitivity (Se) and Specificity (Sp) estimates, with an externally supplied value p, the frequency of positive results:

$$PPV = \frac{p \times Se}{p \times Se + (1 - p) \times (1 - Sp)} \tag{2}$$

2. Positive Predictive Value in relation with HPV-RNA-seq

Our study was aimed at evaluating the prediction performance of our model with three classes, including two classes of lesions :

- *HSIL*, high grade lesions
- *LSIL*, low grade lesions

as well as a a negative class,

- *NILM*, no lesion

The frequency of these different classes is unknown in the population under investigation. Let p_H be the frequency of high-grade lesions, and p_L the frequency of low grade lesions arising due to infection with HPV ; $1 - (p_H + p_L)$ being the frequency of *NILM*.
We assume p_H is low ($p_H \leq 0.01 \leq p_L$)[1] and for simplicity reasons we define the following ratio $k = p_L/p_H$ such that p_L may be defined in relation with p_H, i.e., $p_L = k \times p_H$.

In order to increase performance of models, training was made with two classes only *HSIL* and *NILM* (after removing *LSIL* observation from the training data).

[1](Cuzick J, Cadman L, Mesher D, Austin J, Ashdown-Barr L, Ho L, et al. Comparing the performance of six human papillomavirus tests in a screening population. Br J Cancer. 2013;108:908–13.)

Our purpose here is to define how observations that are randomly drawn from the population of interest (from 3 classes of interest, *NILM*, *LSIL*, *HSIL*) will be predicted by the model with two outcomes, only. We therefore set a, b and c as the probability of an observation from class *NILM*, *LSIL*, and *HSIL* respectively, to be predicted either *HSIL* or *NILM* (see Table 1).

Table 1: Prediction probabilities and population frequency of the different classes (*HSIL*, *LSIL*, *NILM*). Three probabilities are indicated in the three different columns: the probability to be predicted as normal (*NILM*), the probability to be predicted as high-grade (*HSIL*) and the frequency of different lesions in the population of interest.

Class (observed)	Predicted NILM	Predicted HSIL	Frequency in the population
NILM	1 - a	a	1 - $p_H(k + 1)$
LSIL	1 - b	b	kp_H
HSIL	1 - c	c	p_H

Further, we calculate the fraction of *non-HSIL* observations (i.e., both *NILM* and *LSIL*) that will be either predicted *NILM* or *HSIL*, by collapsing rows of Table 1 (see Table 2).

Table 2: Population frequency of observations from classes *HSIL* and *non-HSIL* (which is obtained by collapsing *LSIL* and *NILM*). Expected frequencies of predictions in the population of interest are indicated in the two different columns: the expected frequency of *non-HSIL* and the expected frequency of high-grade lesions (*HSIL*).

Class (observed)	Expected frequency of NILM prediction	Expected frequency of HISL prediction
Non-HSIL	$(1 - a)\ [1 - p_H(k + 1)] + (1 - b)\ kp_H$	$a[1 - p_H(k + 1)] + bkp_H$
HSIL	$p_H(1 - c)$	$p_H c$

Finally we can derive an explicit expression for the Positive Predictive Value:

$$PPV = \frac{TP}{TP + FP} = \frac{p \times c}{p \times c + a \times [1 - p(k+1)] + b \times kp} \tag{3}$$

3. Existing data about commercial assays

For the sake of comparison with our study, estimates of sensitivity and specificity were gathered for a number of commercial HPV assays (see Table 3).

Table 3: Estimates of Sensitivity (Se) and Specificity (Sp) gathered from five different assays:

Assay	Se	Sp
Aptima (Macedo et al. 2019)	0.928	0.605
PreTect Proofer (Derbie et al. 2020)	0.830	0.730
Quantivirus (Derbie et al. 2020)	0.861	0.546
Predica (Andralojc et al. 2022)	0.850	0.720
Aptima (Derbie et al. 2020)	0.914	0.462

Applying the formula above (see Eq. [2]) we calculate Positive Predictive Values relative to the different tests that were released.

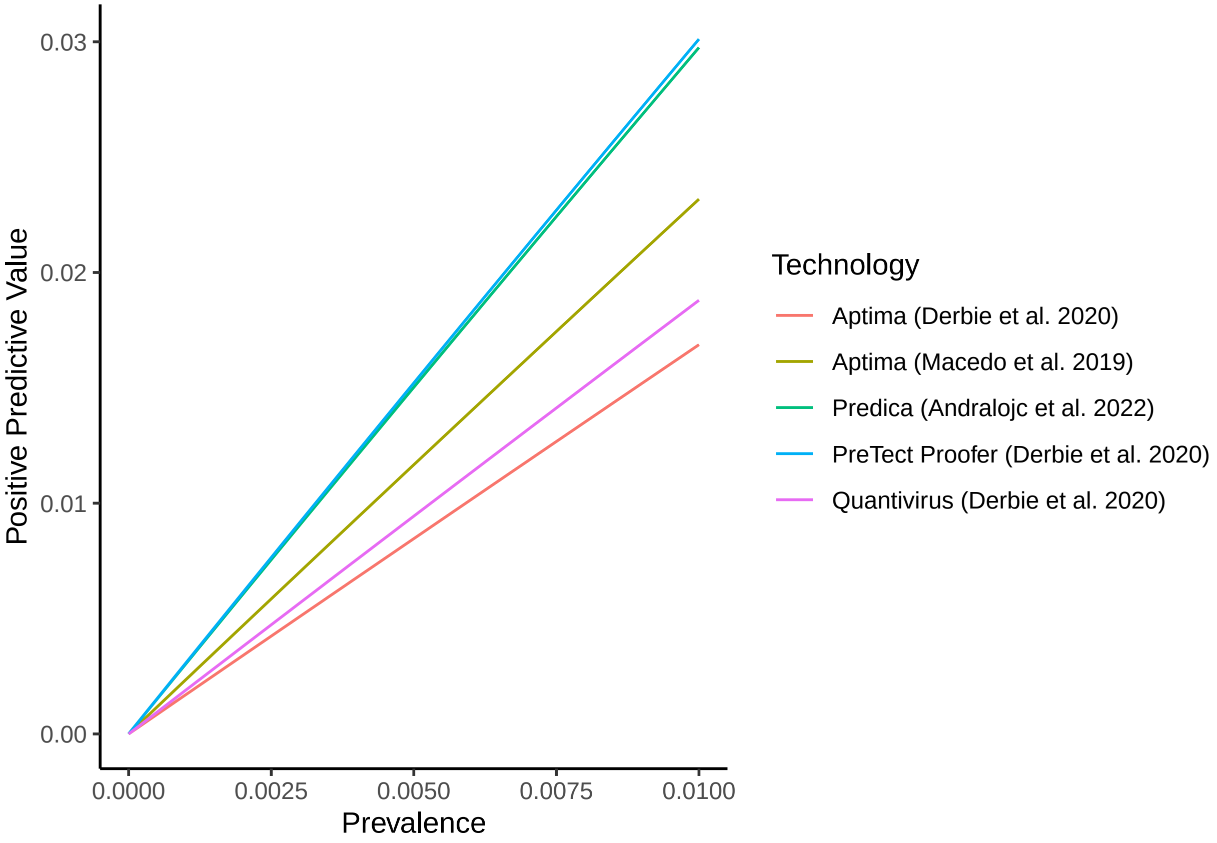

Figure 1: Positive Predictive Value as a function of prevalence of *HSIL* observations

4. Positive Predictive Value of the different models

Applying the formula of Eq. [3] we calculate Positive Predictive Values relative to the different HPV-RNAseq models we considered in this study.

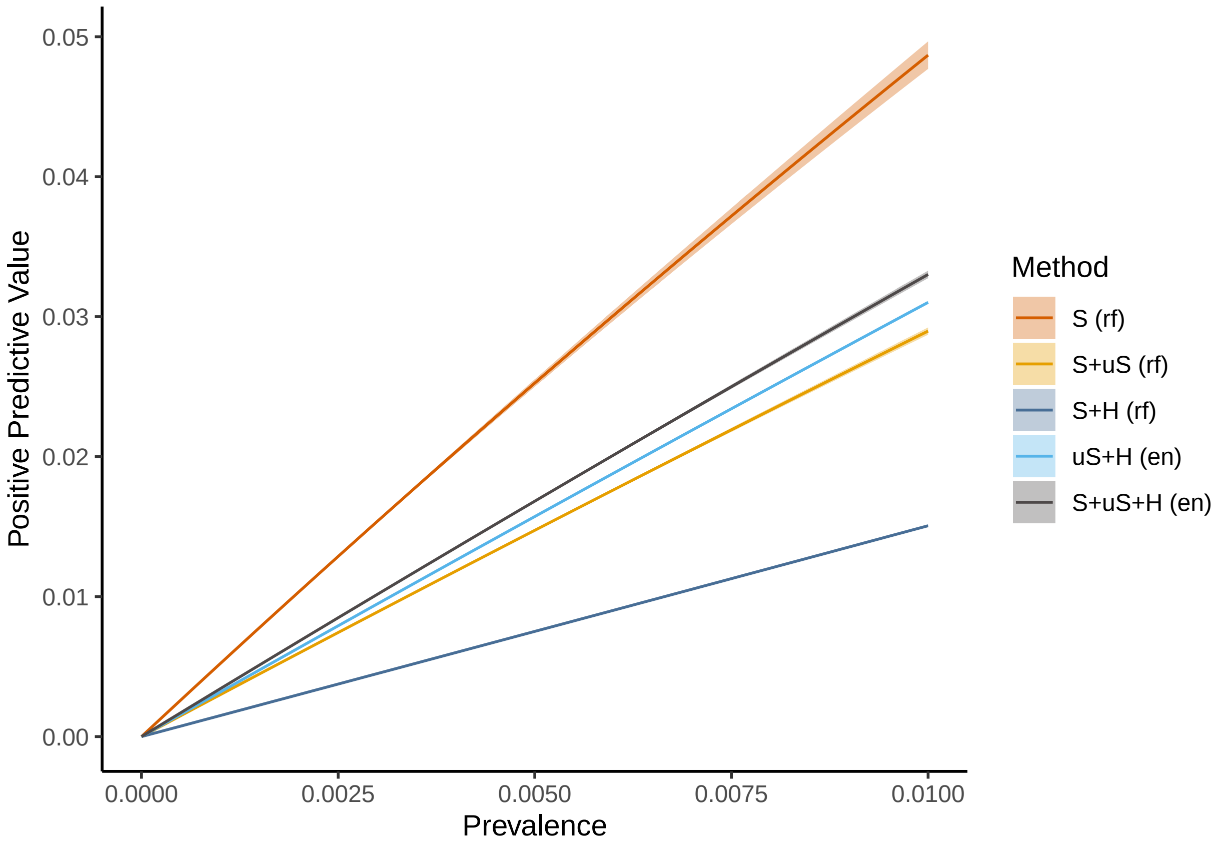

Figure 2: Positive Predictive Value relative to the different models as a function of prevalence of *HSIL* observations

5. All PPV estimates together

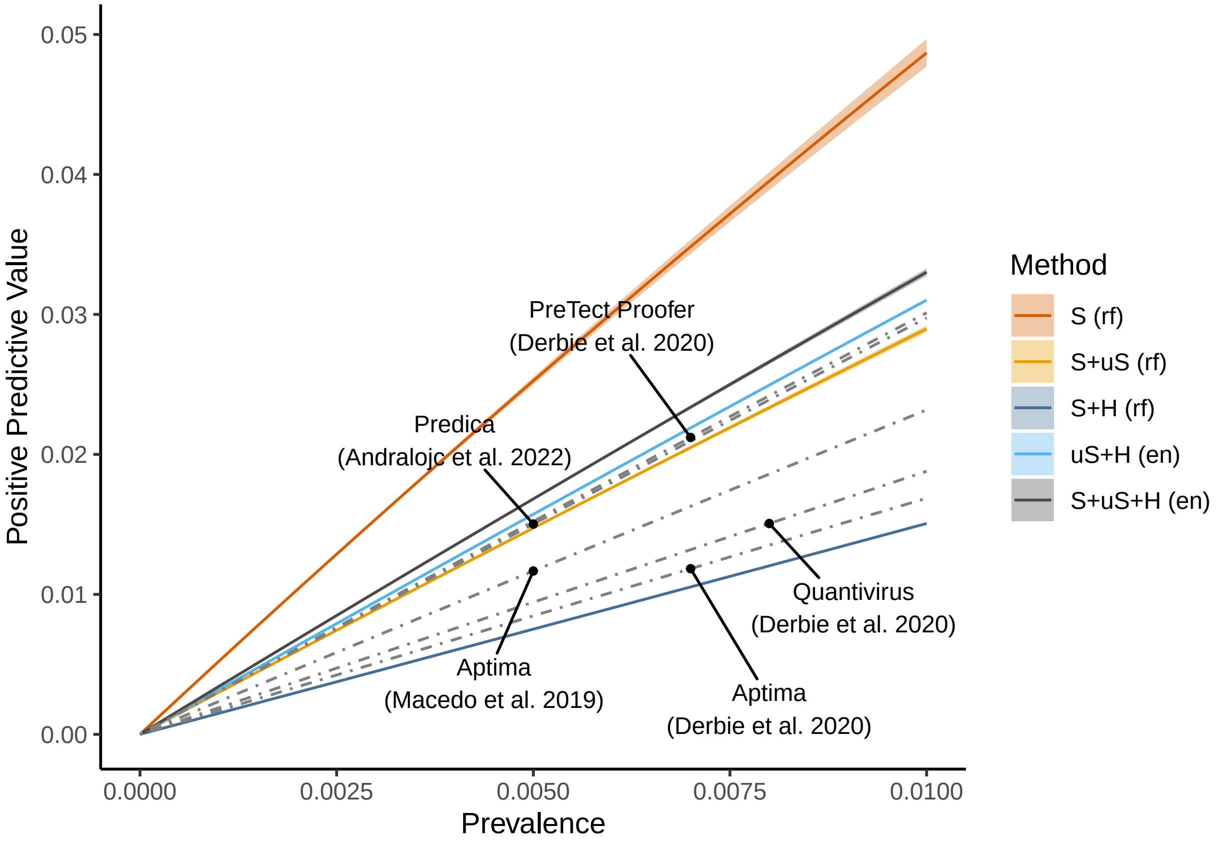

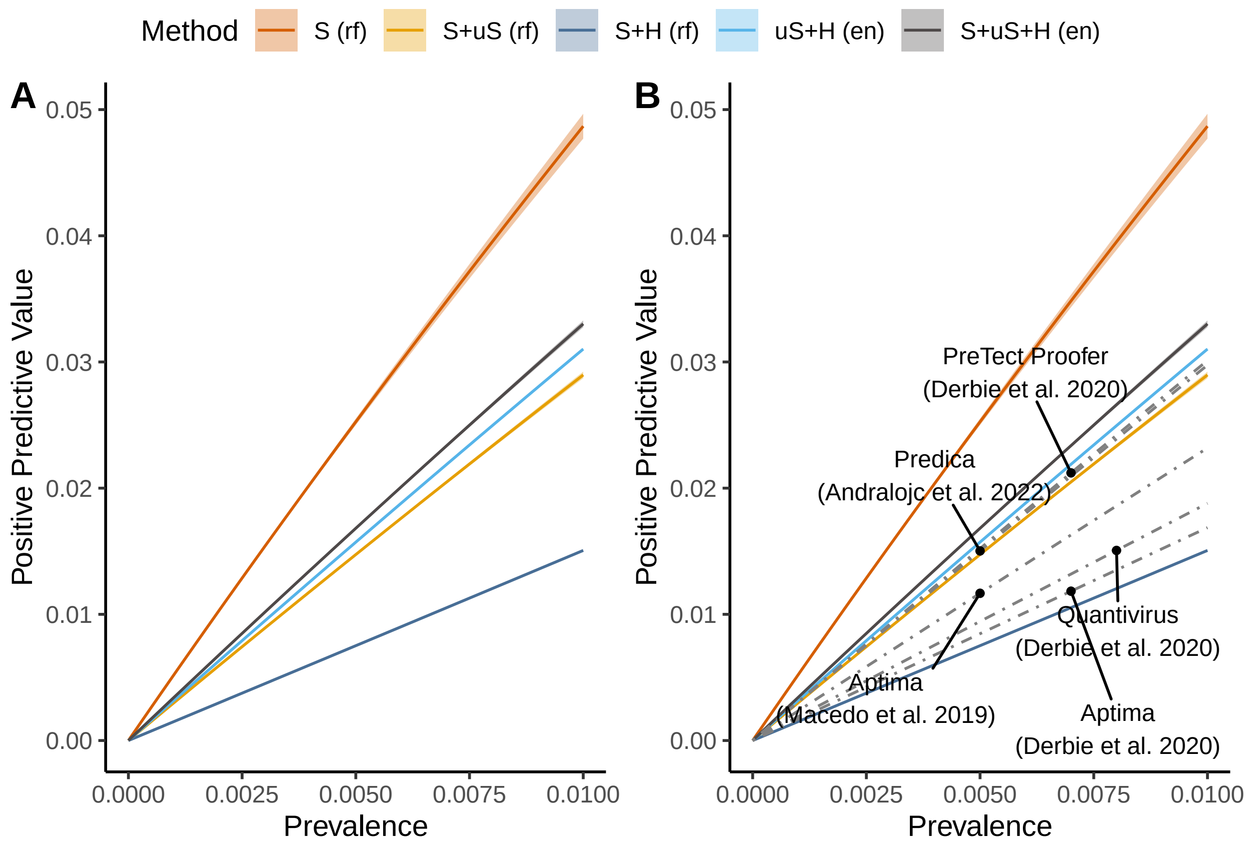
Method
S (rf)
S+uS (rf)
S+H (rf)
uS+H (en)
S+uS+H (en)
A
B
Positive Predictive Value
Prevalence
0.00
0.01
0.02
0.03
0.04
0.05
0.0000
0.0025
0.0050
0.0075
0.0100
PreTect Proofer
(Derbie et al. 2020)
Predica
(Andralojc et al. 2022)
Quantivirus
(Derbie et al. 2020)
Aptima
(Macedo et al. 2019)
Aptima
(Derbie et al. 2020)

```
## pdf
##   2
```
