# Supplementary material for: Molecular detection of hrHPV-induced high-grade squamous intraepithelial lesions of the cervix through a targeted RNA next generation sequencing assay
Source: Mol Med. 2025 May 30;31:215. doi: 10.1186/s10020-025-01238-x (PMC12125924; doi:10.1186/s10020-025-01238-x)
Supplement: Supplementary file 12 — Supplementary Material 12: SupplementaryData. [file 10020_2025_1238_MOESM12_ESM.docx]

**SUPPLEMENTARY DATA**

Molecular detection of hrHPV-induced high-grade squamous intraepithelial lesions of the cervix through a targeted RNA next generation sequencing assay

[**MATERIALS AND METHODS** 2](#_Toc193112996)

[**SUPPLEMENTARY FIGURES AND TABLES** 6](#_Toc193112997)

[Supplementary Figure 1: HPV-RNA-SEQ & Gynie workflows 7](#_Toc193112998)

[Supplementary Figure 2: HPVs detection through samples 8](#_Toc193112999)

[Supplementary Figure 3: Training and validation set composition 9](#_Toc193113000)

[Supplementary Figure 4: Samples distribution into training and validation sets. 9](#_Toc193113001)

[Supplementary Figure 5: Prediction scores of 2-class models 10](#_Toc193113002)

[Supplementary Figure 6: Prediction scores for the 5 best models 11](#_Toc193113003)

[Supplementary Figure 7: Elastic-net coefficients for transcripts models 12](#_Toc193113004)

[Supplementary Figure 8: Features predictive value through HPVs models 13](#_Toc193113005)

[Supplementary Figure 9: Elastic-net coefficients for HPVs models 14](#_Toc193113006)

[Supplementary Figure 10: Prediction of LSIL samples for the five best models. 15](#_Toc193113007)

[Supplementary Figure 11: Prediction scores of LSIL samples for the five best models. 16](#_Toc193113008)

[Supplementary Table 1: HPV-RNA-SEQ AmpliSeq custom panel (WG_WG00141). 17](#_Toc193113009)

[Supplementary Table 2: Sample quality control for RNA, cDNA, libraries, and sequencing data details. 17](#_Toc193113010)

[Supplementary Table 3: Read counts for sequenced samples. 17](#_Toc193113011)

[Supplementary Table 4: Features selection. 17](#_Toc193113012)

[Supplementary Table 5: Performance metrics of 27 explored models. 17](#_Toc193113013)

[Supplementary Table 6: Elastic net coefficients and variable importance across explored models. 17](#_Toc193113014)

[**SUPPLEMENTARY DATA** 17](#_Toc193113015)

[Supplementary Data 1: HPV detection threshold optimization. 17](#_Toc193113016)

[Supplementary Data 2: Exploratory analysis on HPV-RNA-SEQ data. 17](#_Toc193113017)

[Supplementary Data 3: Confusion matrices and samples scores generated for 27 explored models. 17](#_Toc193113018)

[Supplementary Data 4: Boxplots illustrating the importance of variables across the 27 explored models. 17](#_Toc193113019)

[Supplementary Data 5: Positive Predictive Value 17](#_Toc193113020)

[**APPENDIX 1: Exploratory analysis** 17](#_Toc193113021)

[**APPENDIX 2: Applying 2-class models for determining risk for LSIL samples** 17](#_Toc193113022)

[REFERENCES 18](#_Toc193113023)

# **MATERIALS AND METHODS**

**Clinical specimens**

This study included retrospectively collected HPV-positive cervical swabs samples **conserved in l**iquid-based cytology medium from 366 patients, aged from 25 to 65 years, referred for gynecologic consultation in the Centre Hospitalier Universitaire (CHU), Amiens, France. Samples consisted of left-over cervical swabs preserved à -80°C in PreservCyt Solution (Hologic, USA), a widely used transport medium compatible with RNA conservation (1).

**Clinical data**

All samples were HPV DNA positive. Available clinical data encompassed cytology outcomes, and, when accessible, histological data (Figure 1). Patients had virological results from HPV DNA Roche Cobas® (N=204/366) or Anyplex™ II HPV 28 (N=156/366). Six samples lacked virological data. HPV DNA Roche Cobas detects and genotypes HPV18 and HPV16, and indiscriminately detects 12 hrHPVs (31,33,35,39,45,51,52,56,58,59,66,68). Anyplex™ II HPV 28 detects and genotypes 19 hrHPVs (16, 18, 26, 31, 33, 35, 39, 45, 51, 52, 53, 56, 58, 59, 66, 68, 69, 73, 82) and 9 low risk HPVs (6,11,40,42,43,44,54,61,70). The cohort exclusively consisted of patients whose cytological results fell within the categories of NILM, LSIL, HSIL, and atypical squamous cells of undetermined significance (ASCUS). Histological data were available for 48% of the cohort (N=176), when a biopsy (N=152) and/or a conization (N=104) were performed for the patient. Seventy-eight patients had histological data from both a biopsy and a conization. All qualified samples underwent the HPV-RNA-SEQ workflow (Supplementary Figure 1).

**RNA extraction and characterization**

Cervical smears residuals were unfrozen, and two microliters of ZymoBIOMICS Spike-in Control II (Zymo Research) were added. Samples were centrifuged at 14 000 rpm 4°C for 7 minutes, the supernatant was removed, and the pellet was washed with 1 mL of phosphate-buffered saline. Samples were then centrifuged again at 14,000 rpm 4°C for 7 minutes, and the supernatant was removed. Three hundred microliters of TRIzol™ (Thermo Fisher) were added on the pellet and the mix was vortexed. RNA purification was done using Direct-zol™ RNA MicroPrep Kit (Zymo Research) protocol, including on-column DNAse treatment step. Elution volume was of 19µL. The concentration of RNA extracts was measured with a nano-spectrophotometer (Implen NP80). The integrity of RNA extracts was accessed by capillary electrophoresis on Qiaxcel using RNA QC v2.0 cartridge (QIaxcel RNA Quality Control Kit, KG, Qiagen). Samples having RNA concentration greater than 25ng/µL were diluted to 25ng/µL.

**Amplification and sequencing**

Reverse transcription was done using the kit qScript™ cDNA SuperMix (Quantabio) with random hexamers, and a final RNAse H treatment. The kit PowerUp™ SYBR™ Green Master Mix (Applied Biosystems) was used to quantify the housekeeping genes expression ACTB and GAPDH by qPCR and access sample quality. Samples with ACTB and GAPDH values below 33 and 34 respectively were considered adequate. Thirteen samples did not meet this quality requirement. Starting from cDNA, NGS libraries were then prepared using the AmpliSeq for Illumina Custom DNA Panel kit and the AmpliSeq custom panel WG_WG00141, with 21 cycles of amplification before adapter’s ligation. Each sample was barcoded individually. The HPV-RNA-SEQ AmpliSeq Custom Panel (WG_WG00141, Supplementary Table 1) includes in total 525 unique primers, targeting 750 sequences (30 human regions including oncogenes, tumor suppression genes, direct or indirect downstream effectors of HPV oncoproteins AKT1, BCL2, BRAF, CDH1, CDKN2A, CDKN2B, ERBB2, FOS, HRAS, KRAS, MET, MKI67, MYC, NOTCH1, PCNA, PTEN, RB1, STAT1, TERT, TOP2A, TP53, WNT1, and 22 to 34 regions per HPV type), as fully described in Pérot *et al*. (1). The authors categorized different HPV target regions, considering splice events, as follows: “HPV spliced junctions” is a set of target sequences, which are specific HPV splice events, involving a pair of SD and SA sites; “HPV unspliced junctions” is a set of target sequences that are specific HPV genomic regions spanning either SD or SA sites, in the absence of any splice event; “HPV genome” is a set of target sequences that are specific HPV genomic regions, away from any SD or SA sites. Only positive libraries were sequenced. Six libraries were negative. In total, 347 samples underwent sequencing in Illumina NextSeq2000 sequencers (PE 300 cycles). Total number of raw reads varied from 0 to 9.9 million reads per sample (mean: 4.54 million; median: 4.3 million). Comprehensive details on qPCR values, NGS libraries and sequence data output can be found in Supplementary Table 2.

**Sequencing data processing**

Sequence data were processed using the Gynie pipeline (Supplementary Figure 1). Trimming was done using AlienTrimmer (2) version v.2.1.201124ac. Reads were aligned to the reference sequences of the amplicons using STAR (3) version v2.7.11a in local alignment mode (parameter –alignEndsType EndToEnd), by only reporting uniquely mapped reads (–outFilterMultimapNmax 1) and turning off splicing alignment (–alignIntronMax 1). For reference sequences containing a splice junction, only reads mapping at the junction site and encompassing at least 10 bases before and 10 bases after the junction were kept. Read counts for each sequence and each sample are provided in Supplementary Table 3. HPV-RNA-SEQ data from samples were considered adequate if at least 2 housekeeping genes were detected, surpassing an arbitrary threshold of 800 counts each. Four samples failed to meet this quality-control step, leaving a total of 343 analyzable samples. The presence of a specific HPV genotype was determined by (1) calculating the ratio of reads mapping to HPV-specific amplicons to the total number of uniquely mapped reads in a sample, multiplied by 10^6^ (expressed as fragments per million, or FPM); Then (2) varying the detection threshold (in % FPM) and optimizing sensitivity and specificity having clinically validated Anyplex™ II HPV28 and COBAS HPV DNA tests as a reference. An HPV detection threshold of 0.05% FPM yielded the best sensitivity and specificity values between the two tests (Supplementary Data 1). A more detailed view on the identified genotypes is shown in Supplementary Figure 2. Eleven samples were negative for HPV transcriptional activity, with the following clinical outcomes: four were classified as Negative for Intraepithelial Lesion or Malignancy (NILM), three as Low-Grade Squamous Intraepithelial Lesion (LSIL), three as Atypical Squamous Cells of Undetermined Significance (ASCUS) (with histology confirming High-Grade Squamous Intraepithelial Lesion performed within 0-123 days after cytology), and one had no cytological results but histology confirmed HSIL after 69 days. Among these 11 samples negative by HPV-RNA-SEQ, the HPV DNA genotypes detected were as follows: seven samples infected with HPV16, two with HPV18, one with HPV33, one with an unspecified high-risk HPV genotype. More generally regarding genotype concordance with DNA tests, we observed: exact genotype matching (identical lists of HPV genotypes detected) in 25.8% of cases; in 55.5% of cases, HPV-RNA-SEQ detected all genotypes identified by the DNA assays plus at least one additional genotype; in 18.7% of cases, at least one genotype detected by the DNA assays was not detected by HPV-RNA-SEQ. These discordant cases underscore the apparent limitations of RNA-based assays in detecting transcriptionally silent or very low-level infections, which may still be identifiable through DNA-based methods. Nevertheless, whereas DNA-based assays may detect a broad range of HPV genotypes, including latent infections, HPV-RNA-SEQ is specifically optimized for detecting transcriptionally active, clinically relevant HPV infection that may drive cellular transformation. For example, dormant, or inactive HPV infections, while detectable at the DNA level, may exhibit little to no transcriptional activity and are less likely to contribute to progression toward precancerous lesions.

**Exploratory analysis**

Principal Coordinates Analysis (PCoA) was conducted to explore differences in expression between samples. PCoA was based on Bray-Curtis dissimilarity distance and computed using R software (v4.2.1) and ape package. Plots were generated to illustrate the distribution of gene expression (gene by gene) across the three cytological classes (NILM, LSIL, and HSIL), and a statistical test was performed to examine variance in gene expression levels. Additionally, a Spearman correlation test was conducted among all transcripts pairwise (Supplementary Data 2, Supplementary Appendix 1).

**Statistical analyses**

The statistical modeling included exclusively samples with NILM, LSIL or HSIL lesion outcome and was based on a mixed reference combining histology and cytology results, assuming hypothesis derived from empirical observations (4–6): histology was used as reference when available and performed either less than 6 months after cytology when discordance with cytology was in favor of progression, or less than 2 months after cytology when discordance with cytology was in favor of regression. Cytology was used as the reference otherwise. The proportion of samples for which cytological and histological data were combined was similar between the training (40.2%) and validation (42.2%) sets. Discrepancies between cytology and histology in the dataset can be interpreted as under/overdiagnosis of one of the methods, actual biological progression, or regression of lesions over the time interval between two tests, or even the coexistence of lesions at different grades within the same patient (5). Were excluded from the modeling: samples with histologically confirmed adenocarcinoma (n=2) or carcinoma (n=5), ambiguous samples (such as samples having discordant biopsy and conization results) (n=3), samples having ASCUS cytology (n=16, three of which were non infected), plus seven samples were excluded for technical reasons. A total of 302 samples (n=366-64) were finally used for the modeling. Linear (elastic net) and nonlinear (random forest) statistical methods were implemented to distinguish High Grade Squamous Intraepithelial Lesions (HSIL) from normal samples (NILM). In addition, 3-class models using random forest were tested to discriminate between HSIL, NILM and LSIL.

**Predictor variables**

The predictor’s variables were built from different sets of amplicons, expressed in Fragments Per Million (FPM; all Ampliseq sequences being of the same size): (S) “spliced”, included specific HPV splice events found on HPV trancripts; (uS) “Unspliced”, included specific HPV splice donor or acceptor sites in the absence of splice event; (H) “Human”, consisted of twenty-two human transcripts, including oncogenes. Their combined use was also explored: (S+uS) included HPV spliced and unspliced transcripts; (S+H) included HPV spliced transcripts and human transcripts; (uS+H) included unspliced HPV transcripts and human transcripts; (S+uS+H) included HPV spliced and unspliced transcripts plus human transcripts. Furthermore, to address whether HPV type information alone could be predictive of lesions, we used the HPV type information as predictors. In this case the predictor variables were: (P) (“presence of HPVs”), corresponding to the presence/absence of the different HPV genotypes; and (T) (“total HPV sequence count”), corresponding to the cumulative sequences from each HPV genotype.

**Training and validation sets**

The training set included samples coming from seven different sequencing batches (which are groups of samples processed together in the same sequencing run) and three reception batches. The validation set was defined as a completely independent set of samples, coming from one single reception batch (B1) and sequenced independently (221208_VH00537), to include potential technical variability and report results on a scenario close to real usage of the pipeline (Supplementary Figure 3). Details on sample sizes for training and testing sets are displayed in Supplementary Figure 4.

**Features selection**

The first step of feature selection was implemented for random forest (RF) models, using Boruta algorithm (7). A thousand RF models were fitted on the training datasets, computing the average importance for each, as well as the importance of some shadow variables. Only the transcripts presenting an average importance above that of those shadow variables were selected and then used for model training. For elastic-net models, feature selection is included in the model construction: un-informative transcripts will have an associated coefficient set to zero. See Supplementary Table 4 for more details on selected features and their importance.

**Prediction models tuning & training**

All models were tuned and trained using the caret package (8) and following a repeated 10-fold cross-validation procedure: training dataset was divided into 10 folds, each of them used alternatively as testing set. This procedure was then repeated 5 times with different set of folds. The mean performance across all folds and all repeats were then used to select the best set of parameters and a final model was then re-fitted using those parameters. For elastic-net models, best alpha and lambda couple was selected and for random forest only the mtry parameter was tuned, while ntree was set to 1000. RMSE metric was used to train elastic net models and kappa was used for random forest models. The decision threshold for classification was set to a default value of 0.5 to allow for better comparability of predicted scores between all models. The decision to not tune this value for each model individually using ROC curves was made after verifying that such adjusted thresholds did not significantly improve performances on validation set.

# **SUPPLEMENTARY FIGURES AND TABLES**

**
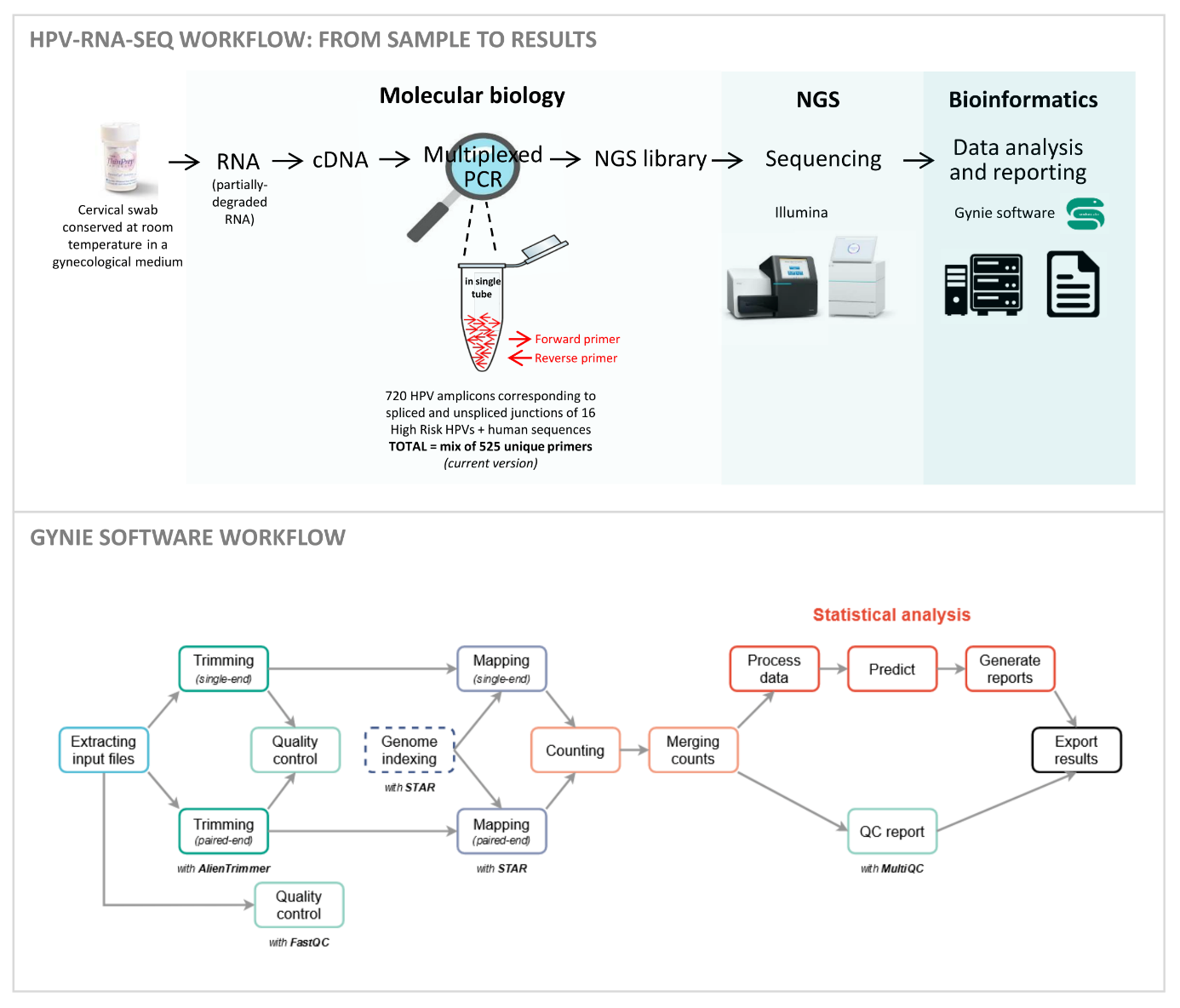
**

## Supplementary Figure 1: HPV-RNA-SEQ & Gynie workflows

Illustration of all steps and main tools used in Gynie workflow, from raw fastq files to readable prediction results and reports. The whole pipeline was developed using Snakemake, R and python scripts.


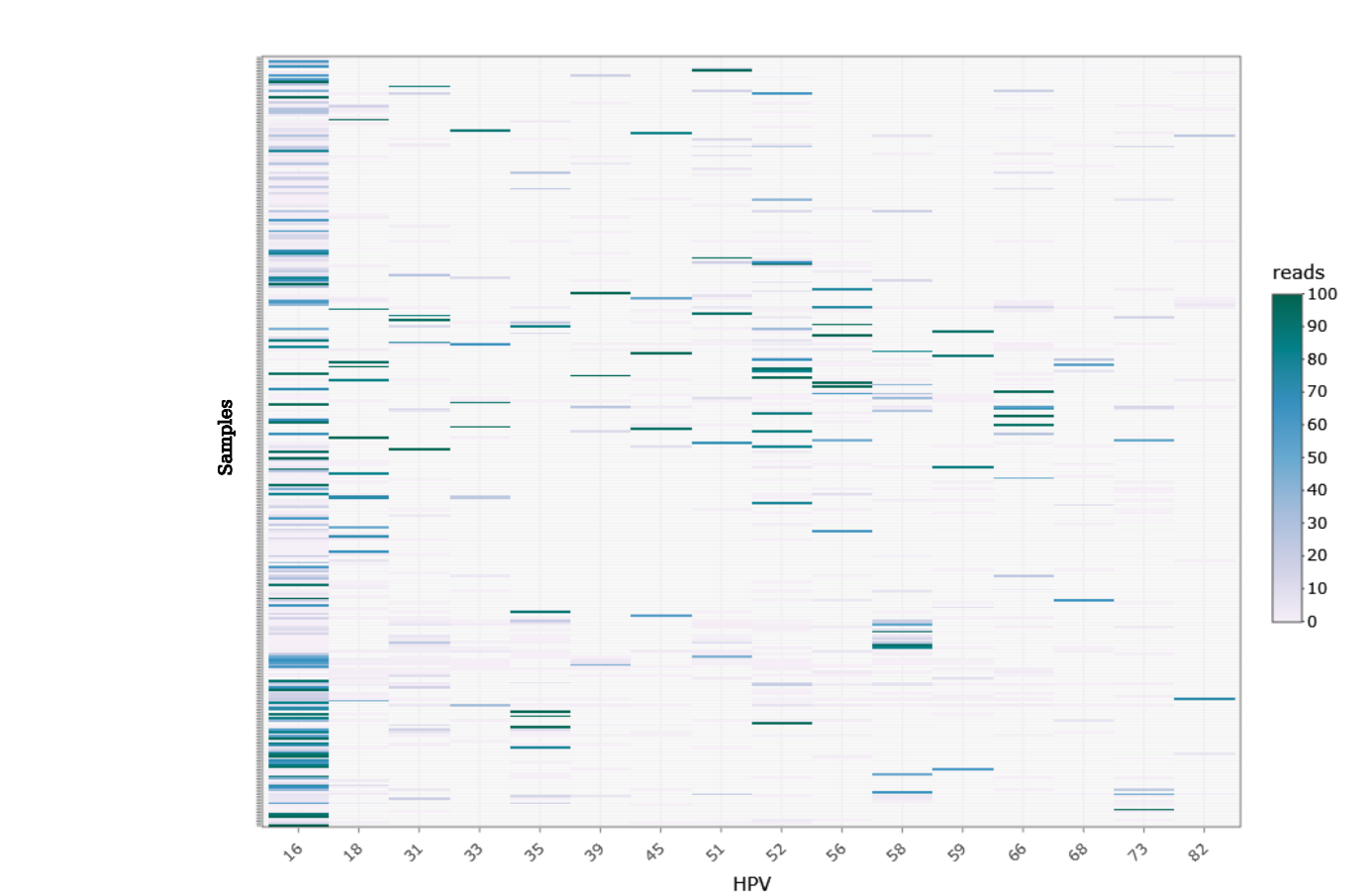


## **Supplementary Figure 2**: HPVs detection through samples

Heatmap representing percentage of reads captured by a given HPV for each sample. All transcripts assigned to an HPV were summed and divided by the total number of reads of each sample.

**
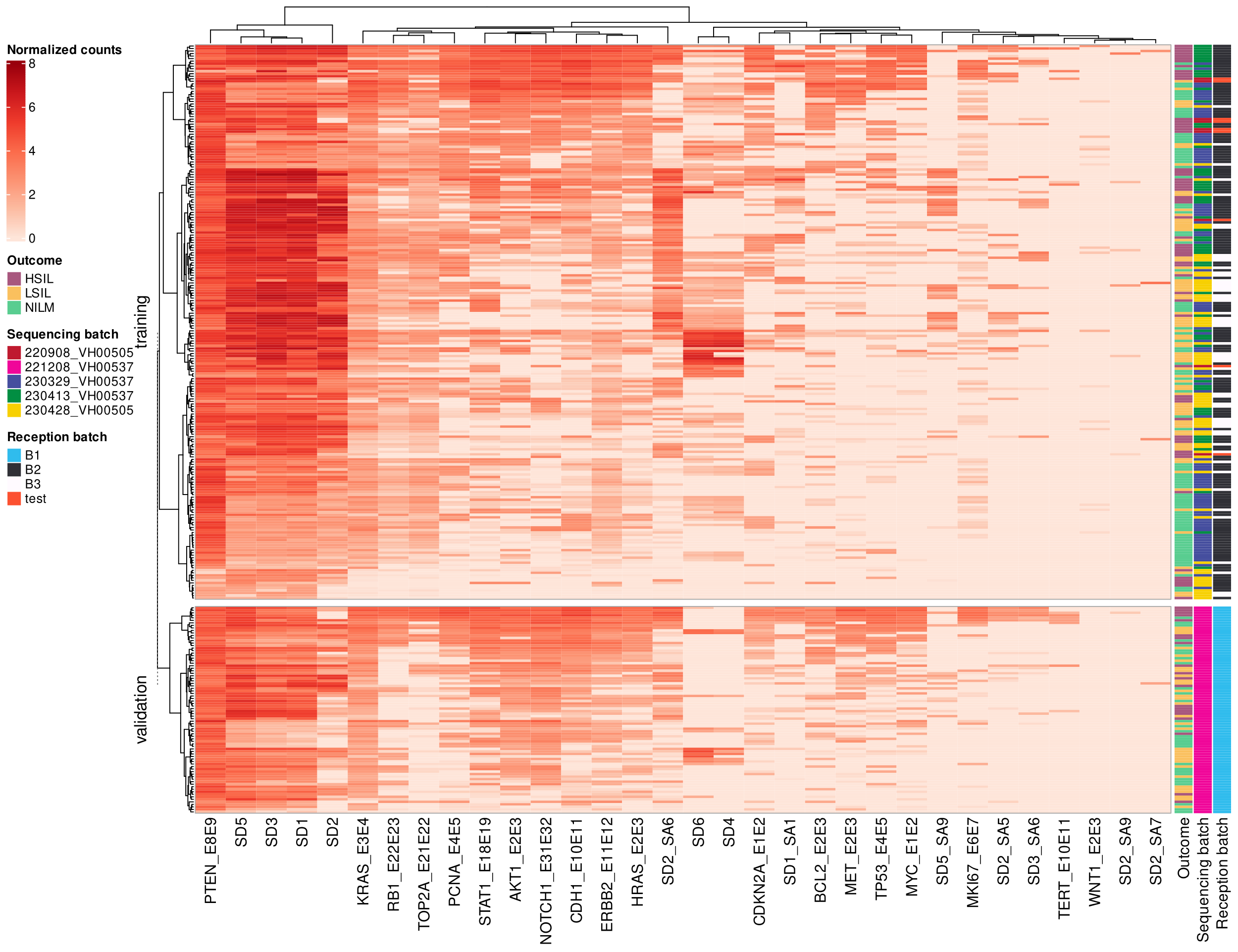
**

Supplementary Figure 3: Training and validation set composition
Heatmap representing the most relevant features counts for all samples, as well as their repartition into training and validation sets. Outcome (NILM, LSIL or HSIL), Sequencing and reception batches are also represented to illustrate each set’s composition.

**
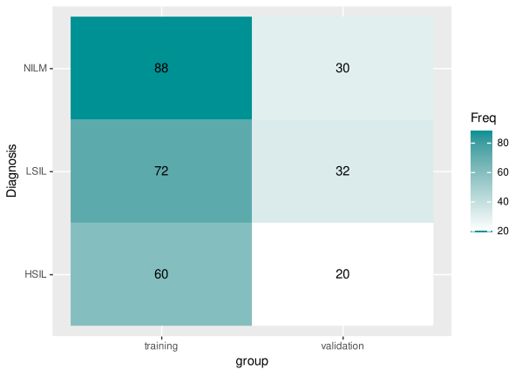
**

## **Supplementary Figure 4**: Samples distribution into training and validation sets.


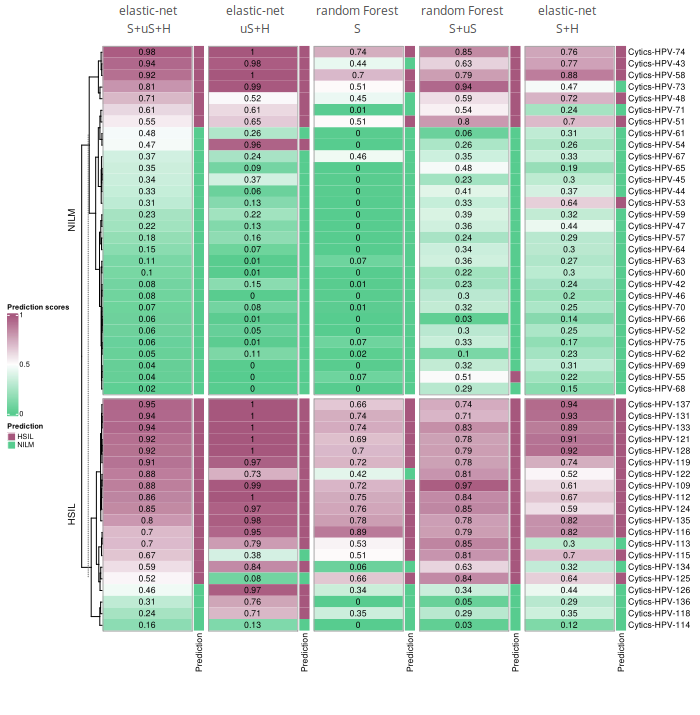


## Supplementary Figure 5: Prediction scores of 2-class models

Heatmap representing the outcome score computed by either random forest or elastic net, for the best five models. Prediction (NILM or HSIL) is made according to whether this score value is below or above 0.5 threshold.

**
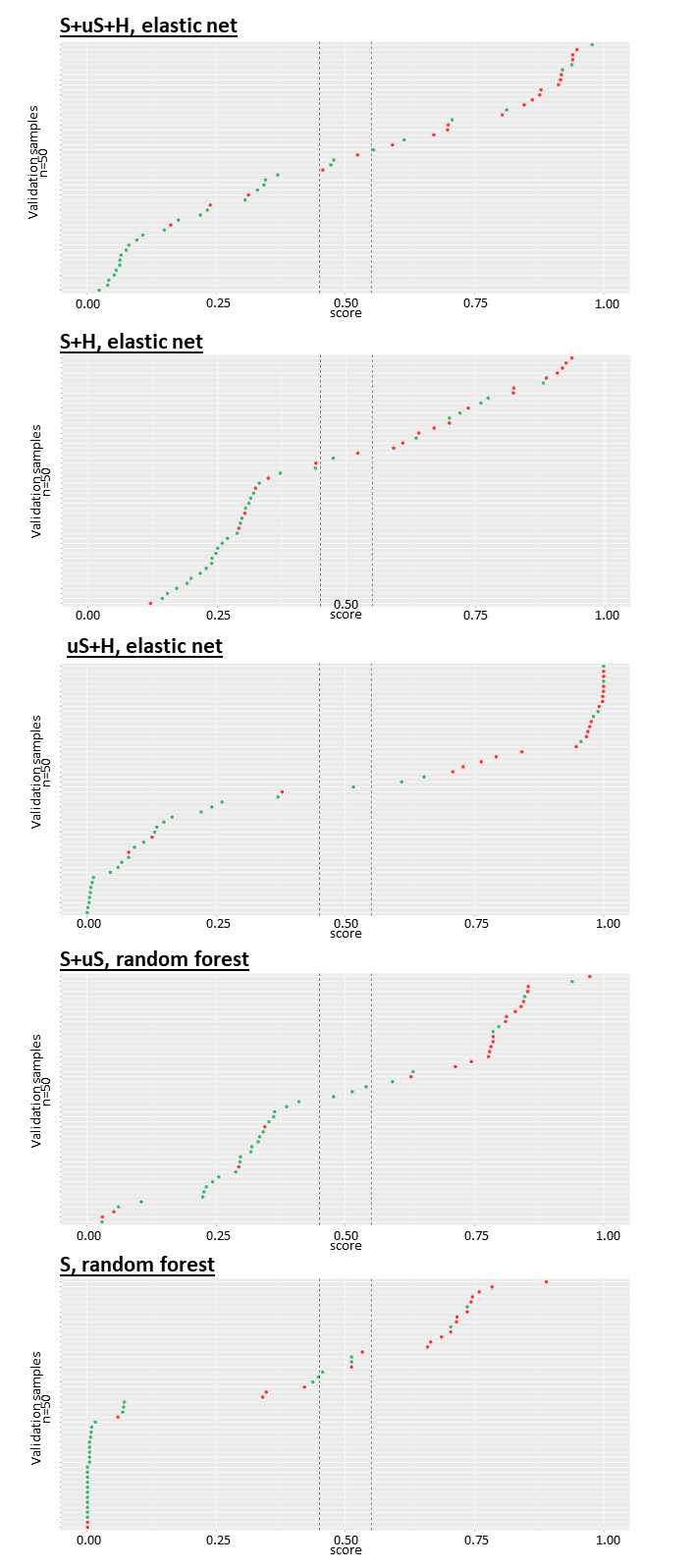
**

Supplementary Figure 6: Prediction scores for the 5 best models
Validation samples are ordered according to prediction score computed by each model. A score below the 0.5 threshold indicates that the sample was classified as NILM, and HSIL otherwise.


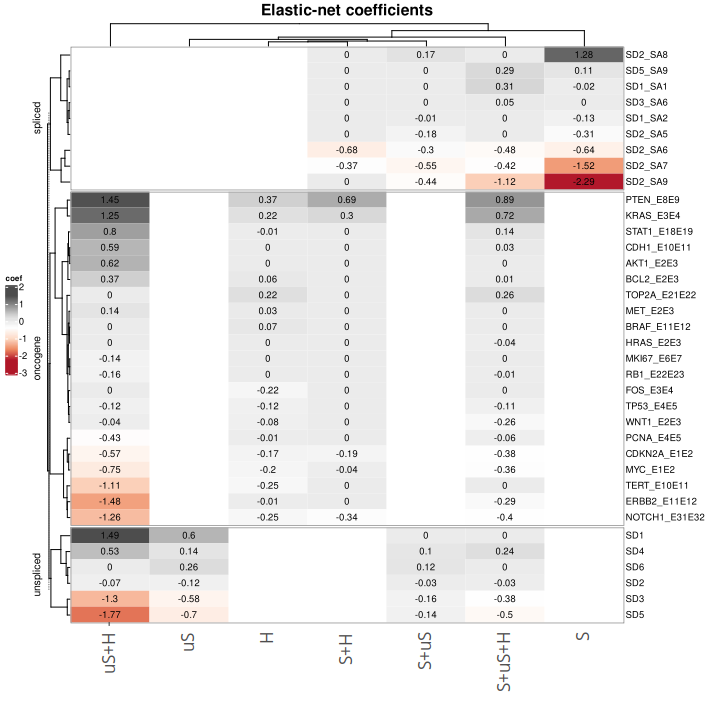


Supplementary Figure 7: Elastic-net coefficients for transcripts models
Heatmap representing the most relevant features selected by at least one of the 2-class elastic net models. Estimated coefficients are represented to highlight features contribution to the prediction.


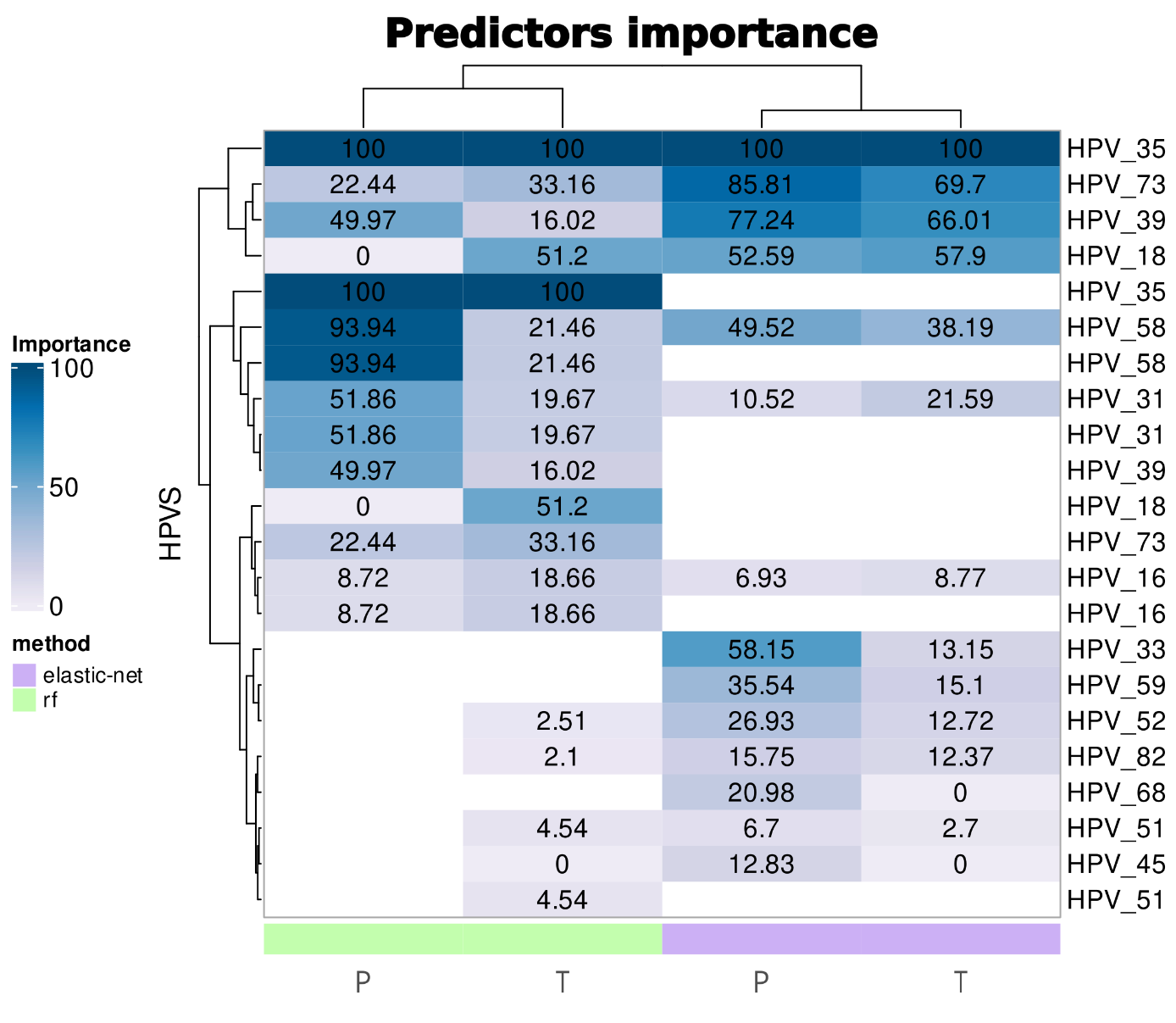


Supplementary Figure 8: Features predictive value through HPVs models

Importance (%) of each HPV use in predictive models (Presence or Total sum). Highly important HPVs are represented in dark blue and less important ones are in off-white. White HPVs were removed during feature selection. Methods (columns) and HPVs (rows) are ordered by hierarchical clustering method, according to Ward D2 criterion. Elastic net method is represented in purple and random forest in green.

**
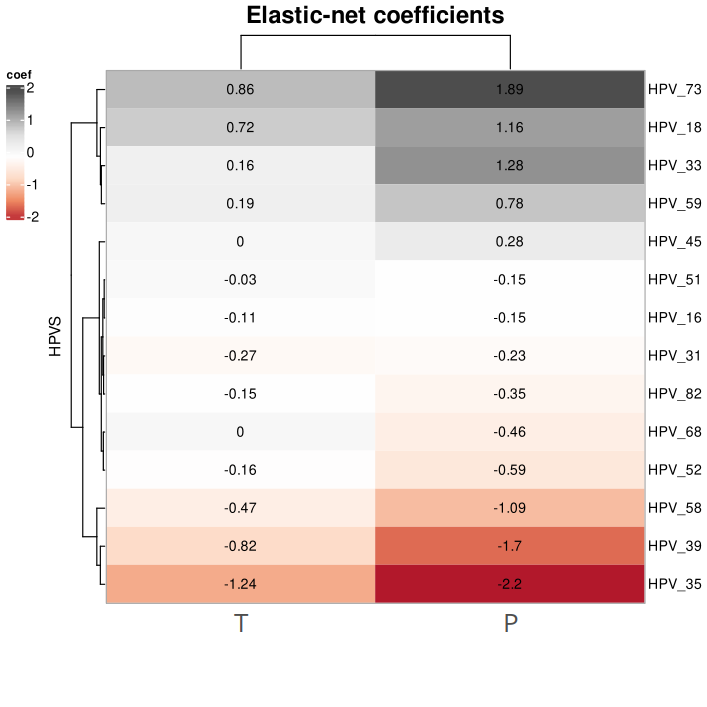
**

## **Supplementary Figure 9**: Elastic-net coefficients for HPVs models

Heatmap presenting the estimated coefficients for HPV use in predictive models (Presence or Total sum). Highly positive coefficients are represented in dark gray and highly negative coefficients are in dark red.

**
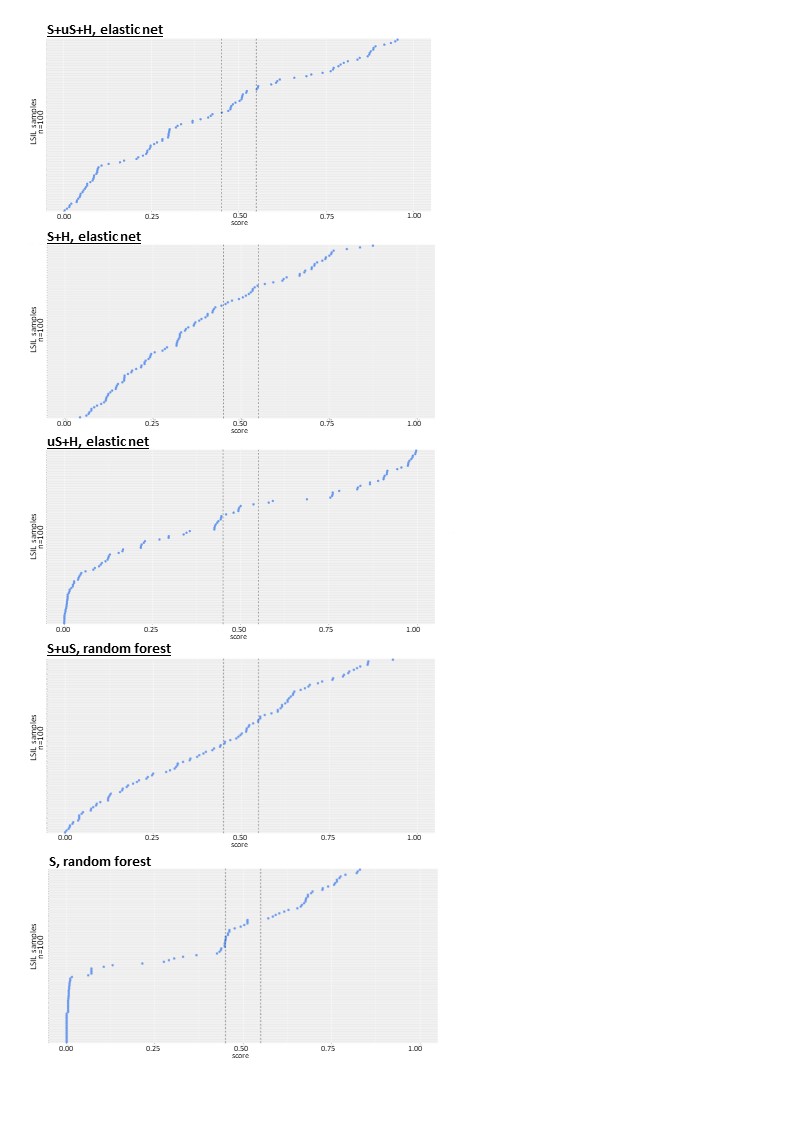
**

Supplementary Figure 10: Prediction of LSIL samples for the five best models.

LSIL samples are ordered according to prediction score computed by each 2-class model. A score below the 0.5 threshold indicates that the sample was classified as NILM, and HSIL otherwise.


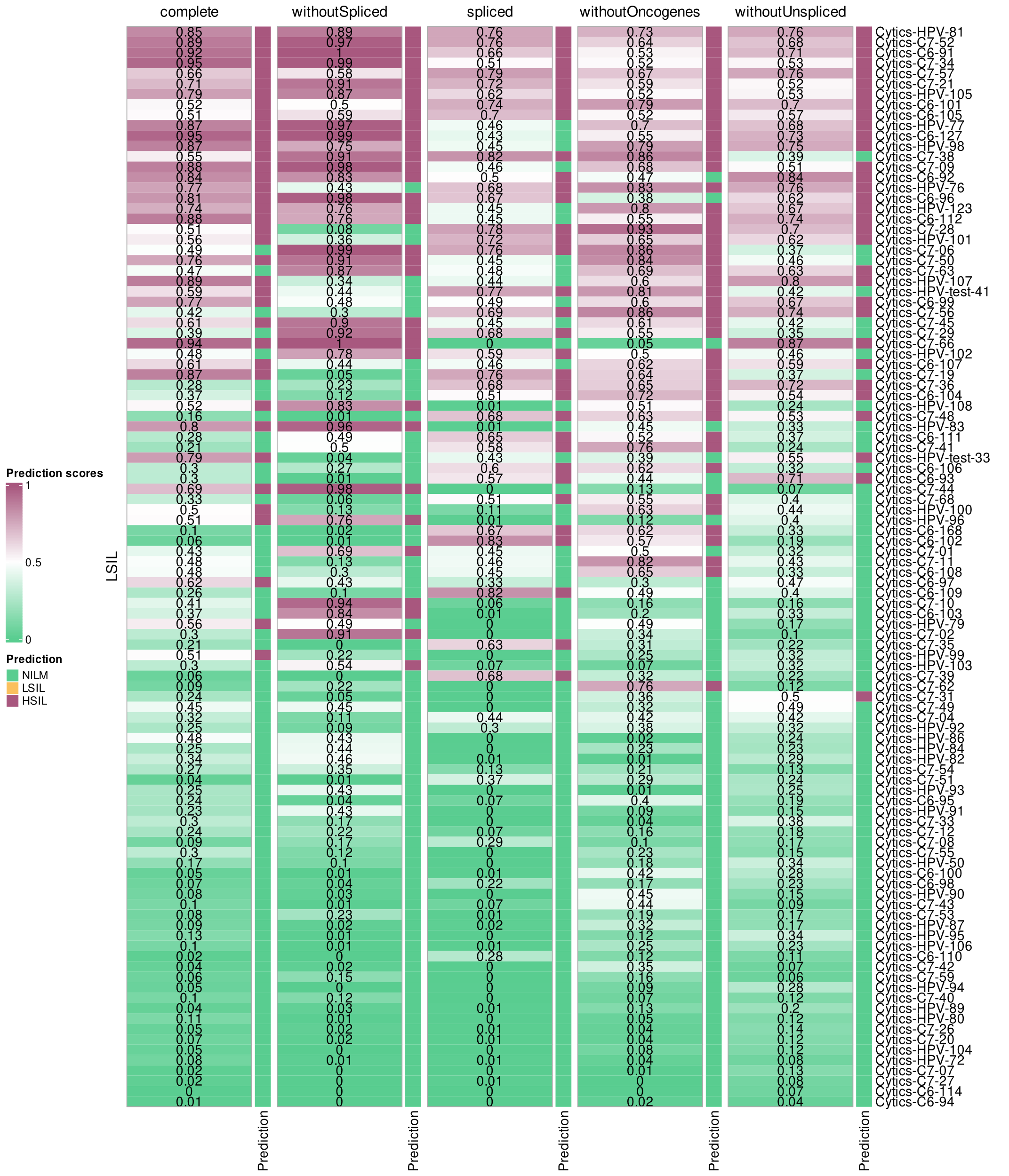


Supplementary Figure 11: Prediction scores of LSIL samples for the five best models.

Heatmap representing the outcome score of LSIL samples by each 2-class model. A score below the 0.5 threshold indicates that the sample was classified as NILM, and HSIL otherwise.

## Supplementary Table 1: HPV-RNA-SEQ AmpliSeq custom panel (WG_WG00141).

## Supplementary Table 2: Sample quality control for RNA, cDNA, libraries, and sequencing data details.

## Supplementary Table 3: Read counts for sequenced samples.

## Supplementary Table 4: Features selection.

## Supplementary Table 5: Performance metrics of 27 explored models.

## Supplementary Table 6: Elastic net coefficients and variable importance across explored models.

# **SUPPLEMENTARY DATA**

## Supplementary Data 1: HPV detection threshold optimization.

## Supplementary Data 2: Exploratory analysis on HPV-RNA-SEQ data.

The exploratory analysis on HPV-RNA-SEQ data included: (1) Principal Coordinates Analysis (PCoAs) plots produced to explore differences in expression between samples and complete interpretation for the models; (2) gene by gene boxplots, to illustrate the distribution of gene expression across the three cytological classes, and statistically test the variance in gene expression levels; and (3) a Spearman Correlation Test among all transcripts pairwise.

## Supplementary Data 3: Confusion matrices and samples scores generated for 27 explored models.

## Supplementary Data 4: Boxplots illustrating the importance of variables across the 27 explored models.

## Supplementary Data 5: Positive Predictive Value

# **APPENDIX 1: Exploratory analysis**

Plotting the gene expression profiles (Supplementary Data 2) revealed an early significant increase in expression of SD1, SD2, SD3, and SD5 from NILM to LSIL and no differences between LSIL and HSIL. In contrast, the spliced variants SD2_SA6 presented a progressive increase in expression from NILM to LSIL and HSIL, and SD1_SA1 showed a rather late increase, with no significant differences detectable between NILM and LSIL, but a significant increase in expression between LSIL and HSIL. Human genes showed variable expression patterns, with PTEN showing a decrease in expression from NILM to HSIL, CDKN2A and STAT1 presenting lower expression values in LSIL, and MYC, NOTCH1 and TP53 showing a late increase in expression with no significant differences detectable between NILM and LSIL, but a significant increase in expression between LSIL and HSIL.

# **APPENDIX 2: Applying 2-class models for determining risk for LSIL samples**

Analyzing the classification of LSIL samples using the five selected models and analyzing the expression profiles of the variables that contributed to these models could provide insight on the predictive value of the models. Thus, we applied the five selected 2-class models to classify LSIL samples (into NILM or HSIL category). This resulted in a heterogeneity of classification results, which in turn suggests a molecular heterogeneity of LSIL samples, as we observed a continuum of scores going from zero to nearly 1 (Supplementary Figures 10 & 11). To further investigate the predictive value of LSIL classification by the five selected models, we ran Principal Coordinate Analysis (PCOA) from all 302 samples. PCOAs conducted without unspliced variants (S and S+H) or without oncogenes (S and S+uS) showed little complexity with samples distributing along a gradient from LSIL to HSIL along a single axis (Supplementary data 2). PCOAs on the complete dataset, or without uS presented similar overall structure with significant information carried along axes 1 and 2, with axis 1 presenting a gradient from NILM to HSIL, and axis 2 differentiation LSIL from NILM and HSIL (Supplementary data 2). PCOA plots of the complete dataset, using S+uS predictors, can be found in the Supplementary data 2 as a representative example, where samples were classified among six categories: true negatives (NILM-NILM), false negatives (HSIL-NILM), and LSIL samples predicted as NILM (LSIL-NILM, false positives (NILM-HSIL), true positives (HSIL-HSIL), and LSIL samples predicted as HSIL (LSIL-HSIL). In both cases, samples predicted as normal (NILM) tended to present low coordinate values along axis 1. This included the center of three groups: true negatives (NILM-NILM), false negatives (HSIL-NILM), and LSIL samples predicted as NILM (LSIL-NILM). Conversely, high-grade lesions (HSIL), including false positives (NILM-HSIL), true positives (HSIL-HSIL), and LSIL samples predicted as HSIL (LSIL-HSIL) presented high coordinate values along axis 1. For the S+uS+H elastic net model (Supplementary data 2), axis 2 carries complementary information where LSIL-NILM, HSIL-NILM, and NILM-HSIL present high coordinate values along axis 2, (associated with 6 unspliced and 2 spliced HPV transcripts), whereas NILM-NILM and HSIL-HSIL present lower coordinate values along axis, associated with expression profiles of 19 human oncogenes. The combination of both axes results in a “semi-circle” pattern with a progression from normal (NILM) with low values on axes 1 and 2, towards LSIL-NILM, HSIL-NILM with higher values along axis 2, then LSIL-HSIL and NILM-HSIL with high values along axes 1 and 2, and finally HSIL-HSIL with high values along axis 1 and low values along axis 2.

REFERENCES

1. Pérot P, Biton A, Marchetta J, Pourcelot AG, Nazac A, Marret H, et al. Broad-Range Papillomavirus Transcriptome as a Biomarker of Papillomavirus-Associated Cervical High-Grade Cytology. The Journal of Molecular Diagnostics. 2019 Sep 1;21(5):768–81.

2. Criscuolo A, Brisse S. AlienTrimmer removes adapter oligonucleotides with high sensitivity in short-insert paired-end reads. Commentary on Turner (2014) Assessment of insert sizes and adapter content in FASTQ data from NexteraXT libraries. Front Genet. 2014;5:130.

3. Dobin A, Davis CA, Schlesinger F, Drenkow J, Zaleski C, Jha S, et al. STAR: ultrafast universal RNA-seq aligner. Bioinformatics. 2013 Jan 1;29(1):15–21.

4. Schlecht NF, Platt RW, Duarte-Franco E, Costa MC, Sobrinho JP, Prado JCM, et al. Human Papillomavirus Infection and Time to Progression and Regression of Cervical Intraepithelial Neoplasia. JNCI: Journal of the National Cancer Institute. 2003 Sep 3;95(17):1336–43.

5. Bruno MT, Cassaro N, Mazza G, Guaita A, Boemi S. Spontaneous regression of cervical intraepithelial neoplasia 3 in women with a biopsy—cone interval of greater than 11 weeks. BMC Cancer. 2022 Oct 18;22(1):1072.

6. Ciavattini A, Clemente N, Tsiroglou D, Sopracordevole F, Serri M, Delli Carpini G, et al. Follow up in women with biopsy diagnosis of cervical low-grade squamous intraepithelial lesion (LSIL): how long should it be? Arch Gynecol Obstet. 2017 Apr 1;295(4):997–1003.

7. Kursa MB, Jankowski A, Rudnicki WR. Boruta – A System for Feature Selection. Fundamenta Informaticae. 2010 Jan 1;101(4):271–85.

8. Kuhn M. Building Predictive Models in R Using the caret Package. Journal of Statistical Software. 2008 Nov 10;28:1–26.
